# Highly Branched Sulfated Glycopolymers as Mucin Mimetics

**DOI:** 10.1021/jacs.5c08232

**Published:** 2025-08-28

**Authors:** Melina I. Feldhof, Rebecca Schlatterer, Friederike Strahl, Jonathan Garthe, Sylvain Prévost, Stephan Schmidt, Matthias Karg, Bizan N. Balzer, Laura Hartmann

**Affiliations:** † Department of Organic Chemistry and Macromolecular Chemistry, Heinrich-Heine-University Düsseldorf, Universitätsstraße 1, 40225 Düsseldorf, Germany; ‡ Institute of Physical Chemistry, Albertstr. 21, 79104 Freiburg, Germany; § Institute of Physical Chemistry I, Heinrich-Heine-University Düsseldorf, Universitätsstraße 1, 40225 Düsseldorf, Germany; ∥ Instrument Responsible D11, Institut Max von Laue − Paul Langevin (ILL), 71, Avenue Des Martyrs − CS 20156, 38042 Grenoble, Cedex 9, France; ⊥ Cluster of Excellence livMatS @ FIT-Freiburg Center for Interactive Materials and Bioinspired Technologies, 9174 University of Freiburg, Georges-Köhler-Allee 105, 79110 Freiburg, Germany; # Physical Chemistry of Functional Polymers, Martin Luther University Halle-Wittenberg, Von-Danckelmann-Platz 4, 06120 Halle (Saale), Germany; ∇ Freiburg Materials Research Center (FMF), University of Freiburg, Stefan-Meier-Str. 21, 79104 Freiburg, Germany; ○ Institute for Macromolecular Chemistry, University of Freiburg, Stefan-Meier-Str. 31, D-79104 Freiburg im Breisga., Germany

## Abstract

Mucins are highly complex glycoproteins that form protective and lubricating barriers around epithelial surfaces, e.g., in the respiratory tract, to protect against pathogens. The isolation and purification of natural mucins without compromising their structure and thus their properties remain challenging. Glycopolymers as mucin mimetics have shown great potential in biomedical research, for example, in mucosal barrier enhancement and respiratory disease treatment, or in improving surface lubrication and adhesion properties. Here, we introduce double-brushed mucin mimetic glycopolymers, replicating for the first time a structural design that more closely imitates key architectural features of natural mucins. By combining solid-phase synthesis of sequence-defined glycooligomers and their attachment onto polyactive ester scaffolds, we enable access to a library of linear, brushed, and double-brushed glycopolymers with controlled variations of structural parameters, such as overall chain length, number, and length of branches, as well as number of carbohydrates and degree of sulfation. By using light and neutron scattering as well as atomic force microscopy-based single-molecule force spectroscopy and imaging, we can demonstrate that the double-brushed architecture is responsible for successfully mimicking critical mucin properties, such as their adhesion to hydrophilic surfaces and an extended conformation, properties that are not achieved with single-brushed or linear analogues. Thus, our findings show that double-brushed sulfated glycopolymers effectively replicate key characteristics of natural mucins, advancing their potential as mucin models, as well as for use in biomedical applications.

## Graphical abstract

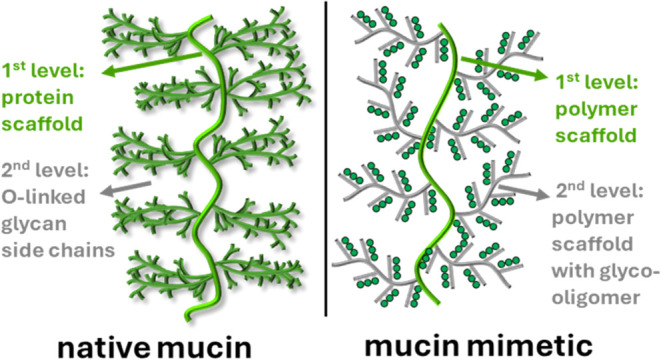

## Introduction

The glycocalyx is a carbohydrate-dense layer on endothelial cells composed of glycoconjugates, such as glycolipids and proteoglycans, known to be involved in various biological functions and processes, such as pathogen adhesion, cellular communication, immune responses, and for its protective function.^1,2^ In particular, mucins, high molecular-weight *O*-linked glycoproteins, form protective hydrogels on epithelial surfaces, shielding cells, e.g., against attachment of pathogens.^3–7^ Mucin molecules are also highly important in the lubrication of various tissues to reduce friction, e.g., in the lung, via hydration lubrication, and as a sacrificial layer. For this, mucin hydration properties are crucial, as water molecules are coordinated by mucins and undergo exchange in response to shear forces, facilitating smooth movement. The hydration lubrication of mucins is enhanced by their high degree of glycosylation, carrying negative charges from sialic acid and sulfated saccharides.^4–6,8,9^ This high degree of glycosylation is achieved by a brush structure with a protein core presenting a high density of branched oligosaccharide side chains. Influenced by these structural features, mucins build up strongly adhering sacrificial layers on underlying substrates or tissue, which are integral to their protective properties, reducing wear. The sacrificial layer mechanism involves the ability of mucins to dissipate energy upon force application, preventing damage to underlying substrates or tissue.^5,9–12^ Thus, mucin hydration, lubrication and protective properties are also closely tied to their adhesion characteristics, with mucin physisorption mediated via van der Waals forces, hydrophobic- and electrostatic interactions and hydrogen bonds.^4,9,11^ Since the isolation, study and potential application of natural mucins is limited by the high dispersity of mucins from different sources and challenges in isolating purified samples or synthesis of natural mucins,^13^ synthetic mimetics of mucins have been developed, e.g., based on anionically charged polymers, glycopolymers and hydrogels.^14–18^ Synthetic glycopolymer mimetics have been designed to replicate key features of mucins, such as their negative net charge, molecular weight, presence of hydrophobic segments, and disulfide cross-linking.^4,9,11^ They have been successfully used as a versatile class of adhesive polymers and model systems to provide insights into the functional role of mucins, e.g., in pathogen adhesion.^19,20^ Based on the brush topology of natural mucins, also mucin mimetic polymers have been developed in various brush-type structures, including branching, dendrimers, or hydrogel formation.^14,21–25^ However, when looking more closely at the natural mucin structure, we find that this exhibits two levels of branching: First, the oligosaccharide side chains themselves are branched structures with monosaccharides as branching points. Second, these side chains are then positioned in a brush-type structure on the protein core (Figure 1). Mucin mimetic glycopolymers so far have included only one level of branching, neglecting the branching within the oligosaccharide side chains. Here, we introduce double-brushed glycopolymers by combining the solid-phase synthesis of sequence-defined glycooligomers. We mimic the oligosaccharide motifs with polyactive ester scaffolds for the conjugation of these glycooligomers to achieve the second level of branching. This results in what we call double-brushed or brush^2^ glycopolymers (Figure 1). Based on the topology, we characterize these glycopolymer mimetics as mucin mimetics; however, they also contain features such as higher sulfation and charge density that can be attributed to sulfated glycosaminoglycans (sGAGs) or sGAG-conjugates such as proteoglycans. This is indeed one of the advantages of these mimetic systems, where we can create non-natural hybrids and, from the controlled variation of structural parameters, gain knowledge that also supports the fundamental understanding of the natural blueprints.

**Figure 1:**
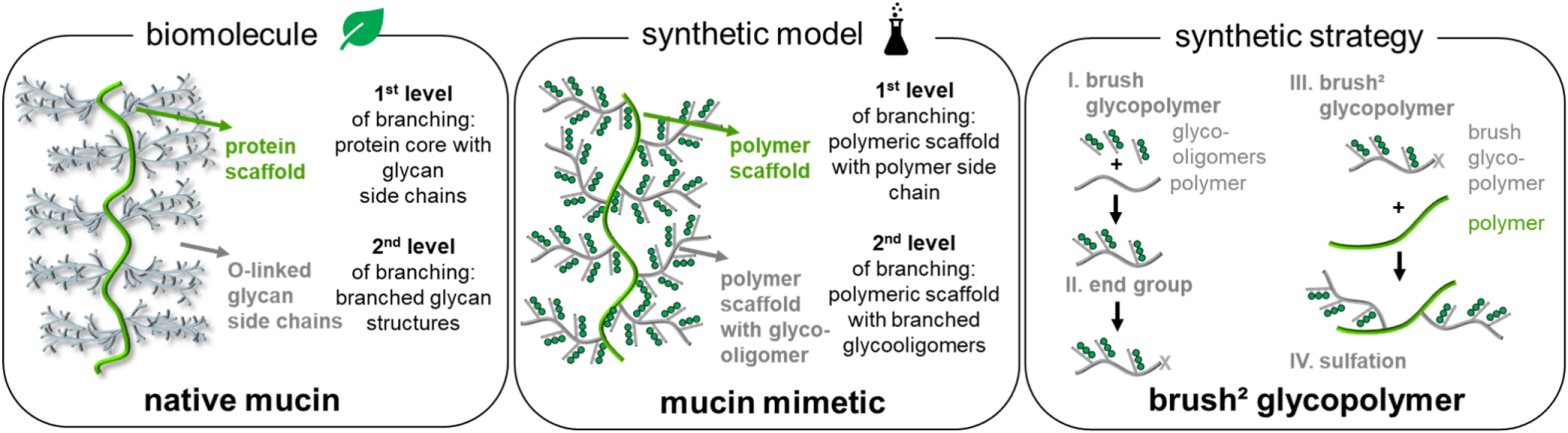
Schematic representation comparing native mucins and mucin mimetics based on the presented double-brushed glycopolymers and the synthetic strategy to derive two levels of branching.

The preparation of brush glycopolymers can be achieved mainly via three strategies: (a) a *grafting-to* approach, where a presynthesized polymer with a reactive end group is attached to the reactive side chain of another polymer, (b) a *grafting-from* approach, where a polymer chain is growing from a macroinitiator, usually another polymer with several initiator units in the backbone, and (c) a *grafting-through* approach, where macromonomers, polymer chains with, e.g., vinyl end groups are polymerized to obtain a brushed polymer.^26,27^ Glycopolymers synthesized via postmodification reactions, such as *grafting-to*, provide a flexible method to customize their properties for applications such as selective interactions with lectins, cellular receptors, or other biomolecules.^28^ These reactions involve adding specific functional groups to preformed polymers, enabling precise control over the glycopolymer structure and properties.^29,30^ Additionally, postmodification allows for the introduction of various carbohydrate residues, adjustment of ligand density, and optimization of the spatial arrangement of functional groups, all of which influence interactions with biological targets.^31^ Well-established strategies to perform postpolymerization use click chemistry, such as azid-alkyne, thiol–ene, or maleimide, isocyanate-amin, epoxide-alcohol, and transesterification reactions.^32,33^ Especially, the transesterification of an active ester polymer, such as poly*N*-hydroxysuccinimide (pNHS) and polypentafluorophenyl (pPFP), is very efficient without competing side reactions and can be performed under neutral conditions, at low temperatures, without the requirement for inert reaction conditions or addition of catalysts.^34,35^ Here, we combine sequence-defined, glycan-functionalized oligomers via solid-phase synthesis and controlled polyactive ester scaffolds to mimic the double-brushed, hydrophilic glycopolymer part of native mucins, key in the mucosal barrier for biolubrication and protection against pathogens.^7,36–38^ While our current focus is on the hydrophilic region, these findings lay the groundwork for including additional functional features and toward future applications, e.g., in wound healing, mucosal barrier enhancement, and respiratory disease treatments. Furthermore, we will include the use of our mucin mimetics in the development of glycocalyx mimetics to extend on our previous work, systematically exploring the structural features of glycoconjugates and their effect on dynamic glycan-mediated attachment.^39,40^

## Results and Discussion

### Synthesis Strategy for Double-Brushed (Brush^2^) Mucin Mimetics

Our synthetic strategy follows a three-step (**A**–**C**) procedure (Figure 2). Specifically, first sequence-defined glycooligoamidoamines **O1** and **O2** are synthesized via solid-phase polymer synthesis (SPPoS). In short, in SPPoS, tailor-made building blocks representing artificial fluorenylmethoxycarbonyl protecting group (Fmoc) protected amino acids or highly defined synthetic building blocks are assembled on a solid support, in particular, a TentaGel resin, through stepwise addition reactions, giving access to monodisperse, sequence-defined scaffolds. By the selection of the appropriate building block, various functionalities can be individually designed. We have previously introduced the building blocks EDS^41^ (ethylene glycol-diamine-succinic acid, 1-(9*H*-fluoren-9-yl)-3,14-dioxo-2,7,10-trioxa-4,13-diazaheptadecan-17-oic acid), to tune length and hydrophilicity, and TDS^42^ (triple-bond diethylenetriamine-succinic acid, 1-(fluorenyl)-3,11-dioxo-7-(pent-4-ynoyl)-2-oxa-4,7,10-triazatetra-decan-14-oic acid) to provide a functional side-chain alkin-functionality for further functionalization. Mannose-azide ligands can then be attached via a CuAAC followed by de-*O*-acetylation under Zemplén conditions.^43^ Importantly, glycooligomers need to carry a free terminal amine group for later conjugation onto the active ester polymer scaffolds. To accomplish this, the *N*-terminal Fmoc protecting group of the terminal building block is cleaved before the glycooligomer is retrieved from the resin. In total, two glycooligomers, **O1** and **O2**, with controlled variation of length and the number of mannose units, are obtained in high purity and analyzed via ^1^H-NMR, RP-HPLC-MS, MALDI, and HR-ESI (Figure 2A, and Supporting Information). In the second step (Figure 2B), poly­(pentafluorophenyl acrylate) active esters p­(PFPA) scaffolds are synthesized via reversible addition–fragmentation chain-transfer (RAFT), employing benzylsulfanylthiocarbonylsulfany propionic acid (BSTPA) as a RAFT agent, following established protocols.^25,44,45^ Overall, three p­(PFPA)­s (**P1**–**P3**), varying in chain length, are obtained (Figure 2B) with degrees of polymerization of 29 (7.2 kDa), 57 (13.8 kDa), and 235 (56.2 kDa), respectively, showing narrow dispersities (*Đ* = 1.26–1.37) (Supporting Information). These active ester polymers are additionally quenched with ethanolamine (**P4**–**P6**) to simplify the analysis and both the p­(PFPA) (**P1–P3**) and the quenched p­(HEAA) polymers (**P4**–**P6**) are analyzed using ^1^H-NMR, ^19^F-NMR, and SEC (see Supporting Information).

**Figure 2:**
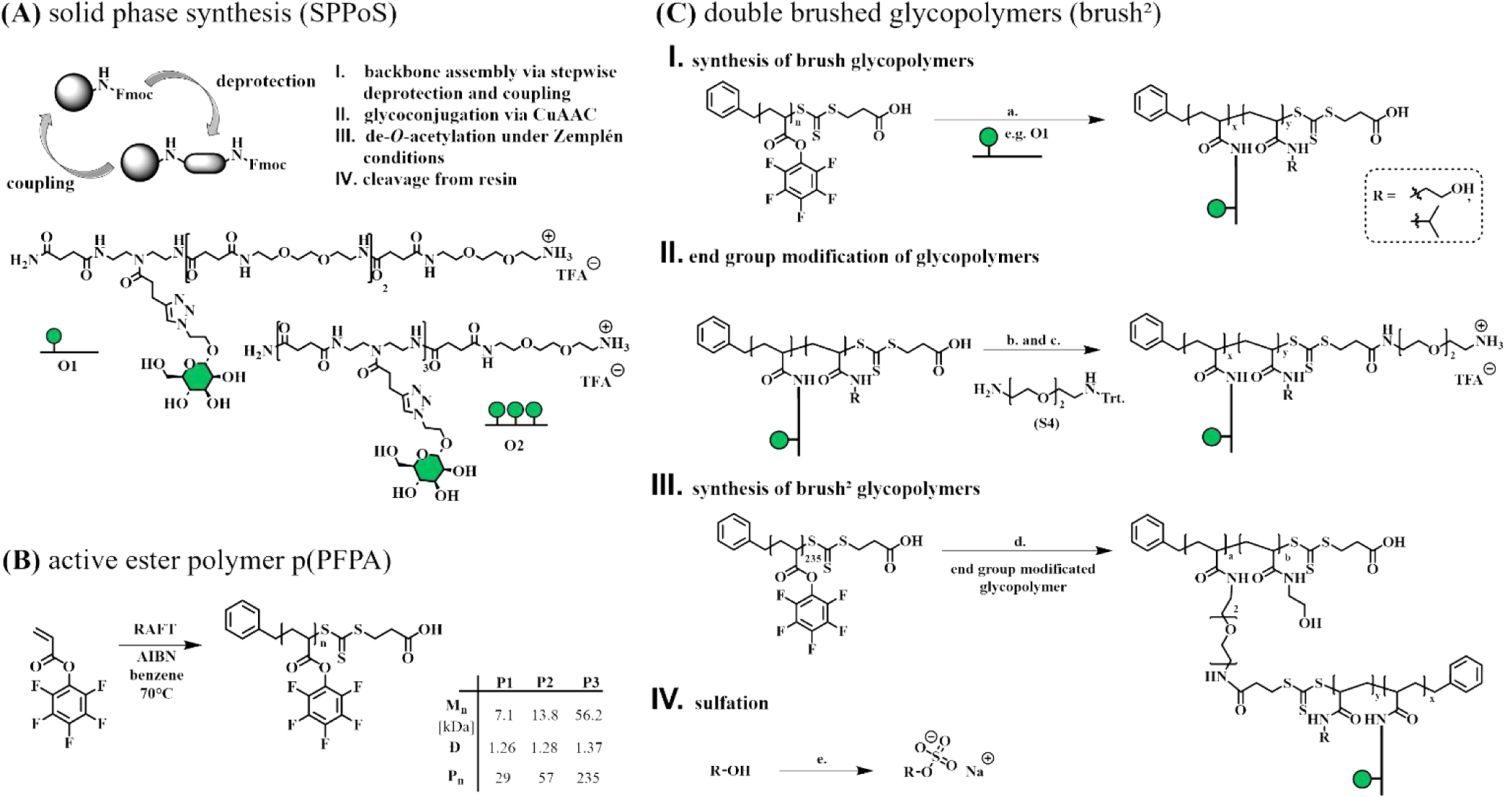
Polymer and oligoamidoamide synthesis for double-brushed glycopolymers. (A) Schematic of Fmoc peptide coupling via solid-phase synthesis for monovalent- (**O1**) and trivalent- oligoamidoamide (**O2**). (B) RAFT polymerization of p­(PFPA) with 29 (**P1**), 57 (**P2**), and 235 (**P3**) repetition units. (C) Synthetic route for double-brushed glycopolymers: I. synthesis of brush glycopolymers – a. p­(PFPA) **P1** or **P2** in DMF, 40 °C, pH > 8 with NEt_3_, oligoamidoamide **O1** or **O2** incorporated at 15% ratio, stirred 24 h followed by quenching with excess ethanolamine or isopropylamine, stirred 24 h at 40 °C, precipitated in cold acetone or diethyl ether, purified by dialysis. II. end group modification of glycopolymers – b. glycopolymer in DMF, terminal carboxylic acid preactivated with EDC*NHS chemistry, linker (**S4**) added, stirred 24 h at room temperature, purified by dialysis – c. dissolved in DMF, diluted with DCM, Trt. protecting group cleaved with 95% TFA and 5% TIPS for 1 h, amine-functionalized polymer precipitated in diethyl ether. III. synthesis of brush^2^ glycopolymers – d. product from II. in DMF, swelled 1h at pH > 8 (NEt_3_). p­(PFPA) **P3** (number of repeat units = 235) in DMF, 40 °C, pH > 8 with NEt_3_, swollen product from II. added at 5% ratio, stirred 24 h, followed by quenching with excess ethanolamine, and stirred 24 h at 40 °C. Brush^2^ glycopolymer precipitated in cold acetone, purified by dialysis (MWCO 50 kDa). VI. sulfation – e. 40 eq TMA*SO_3_ per OH-group in DMF, stirred 24 h at 70 °C, aqueous sodium acetate solution (10 wt %) added at 0 °C, dialyzed (cutoff: 50 kDa).

In the third step of our synthetic strategy, we utilize postpolymeric reactions to create highly brushed glycopolymers with controlled variations in the degree and length of branching, carbohydrate functionalization, and negative charges. The used synthesis route of the double-brushed polymers (brush^2^) is built up chronologically in four steps **I**–**IV**, which is shown as an example in Figure 2C. Step **I.** The synthesized **O1** and **O2** are incorporated in a 15% ratio in the p­(PFPA) derivatives **P1** and **P2**, while all other PFP functionalities are quenched with ethanolamine or in a second sample with isopropylamine. Thereby, p­(*N*-2-hydroxyethyl acrylamide) and p­(*N*-isopropylacrylamide) main chain polymers with glycooligomer side chains are derived. P­(NIPAM) is a thermoresponsive polymer that undergoes reversible phase transitions at physiological temperatures. In our mucin mimetics, this enables modulation of the polymer conformation, analogous to temperature-dependent changes in viscosity and interactions also observed with native mucins, and allows us to study this feature in more detail in future studies.^4^ The brush glycopolymers produced in this step then serve as side chains to synthesize the targeted double-brushed (brush^2^) structures. In order to use the brushed polymers as side chains on another polyactive ester scaffold and derive double-brushed glycopolymers, they have to be modified at the terminal carboxylic acid (step **II**). For this purpose, a linker molecule (**S4**), based on 2,2′-(ethylenedioxy)­bis­(ethylamine), is employed, which is triphenylmethyl (Trt) protected on one side and can be conjugated to the carboxylic acid of the polymer brush via EDC*NHS chemistry. In step **III**, the synthesized amine functional brushed glycopolymer is consequently incorporated at a 5% ratio on the higher molecular weight p­(PFPA) (**P3**) precursor, followed by quenching unreacted PFP functionalities with ethanolamine. The derived brush^2^ glycopolymers are isolated by precipitation in diethyl ether, purified by dialysis, and characterized by ^1^H-NMR and ^19^F-NMR. To mimic the negative charges of natural mucin polymers, brush^2^ polymers **B1**–**B8** are then globally sulfated (step **IV**) following previously established protocols^46^ and the sulfation is proven by characteristic proton shifts through ^1^H-NMR (see Supporting Information for further details on the synthesis and analytical data). All synthesized structures, listed in Table 1, follow uniform nomenclature. The capitalized letter describes the quencher or the main part of the glycopolymer side chain backbone (E = ethanolamine, I = isopropylamine). The exponent (top right) indicates whether a structure is present as a brush glycopolymer (no designation) or as a double-brushed glycopolymer (brush^2^). The lower index (right) indicates the length of the glycopolymer side chains in brush^2^ glycopolymers or the general polymer length in brushed or linear structures, the lower index (left) indicates the number of sugar units in relation to the entire polymer, and sulfation is marked by the addition of an asterisk (*) at the top left position.

**Table 1:**
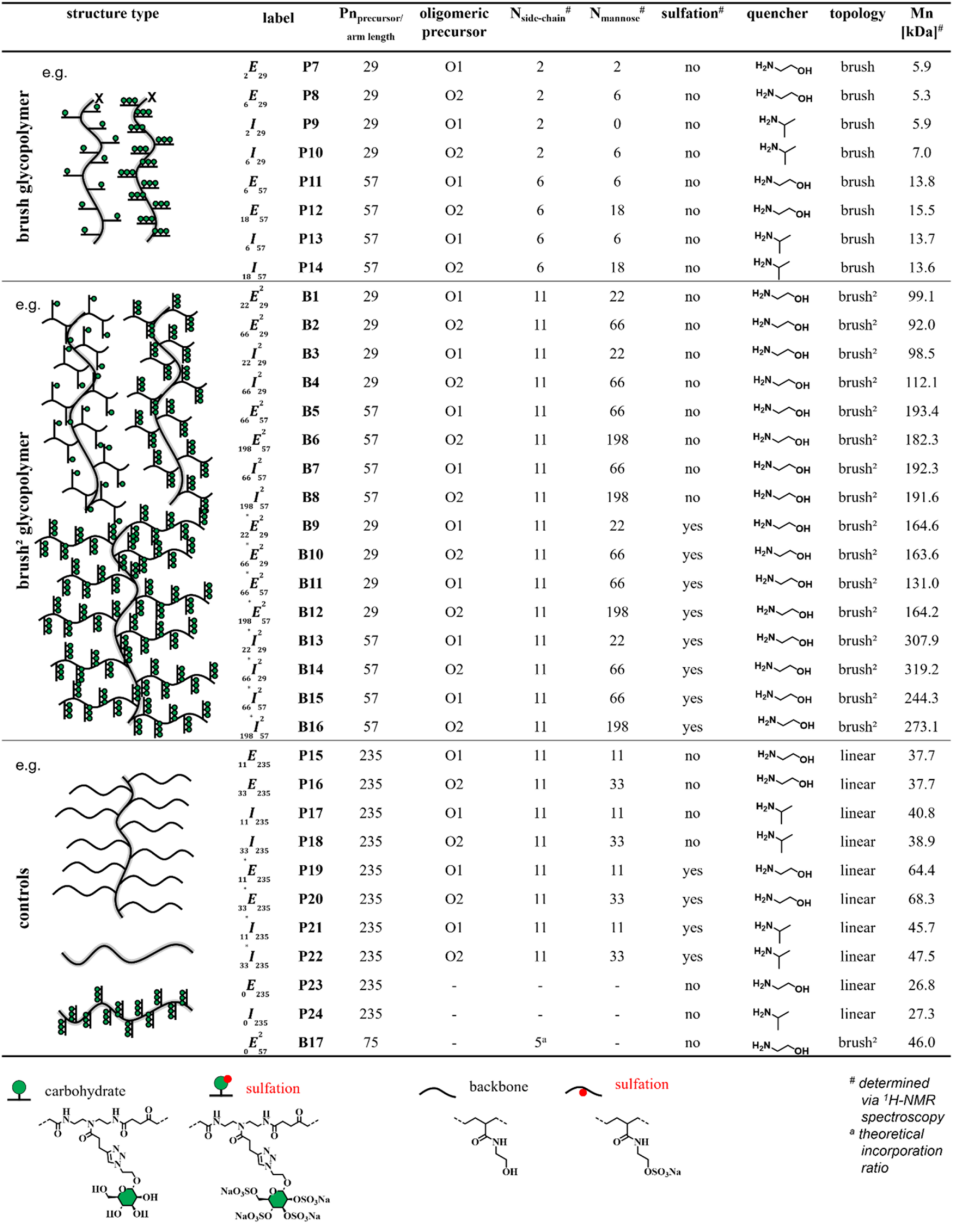
Summary of All Synthesized Linear and Brush Glycopolymers (P7–P24) and Brush^2^ Glycopolymers (B1–B17)

^1^H NMR is also used to calculate and confirm the incorporation efficiency of the functionalities in the linear, brush, and brush^2^ polymers, as well as the overall number of carbohydrates per polymer and the molecular weights (Table 1). The obtained brush glycopolymers (**P7**–**P14**) are isolated, purified via dialysis, and analyzed via ^1^H-NMR, whereby the incorporation ratio of the corresponding oligoamidoamine is determined based on the correlation between triazole protons of the oligoamidoamine and the aromatic protons of the RAFT initiator. The successful conversion of all PFP-active functionalities is confirmed by ^19^F-NMR (see Supporting Information). The integration ratio of the brush^2^ structures is calculated as follows: protons assigned to the triazole unit in the glycooligomers are put in relation to the aromatic protons assigned to the RAFT initiator. The number of mannose units is calculated from the results of the incorporation ratio of the glycopolymer. Figure 3 exemplary showcases the analysis strategy of structure **B5** by ^1^H-NMR that allows one to follow the individual reaction steps. By comparing the ^1^H-NMR of **O1** with the product from step **I**, **O1** is successfully incorporated with six repeat units. Subsequently, the Trt-protected linker (**S4**) is conjugated, which can be qualitatively confirmed by the increase in signal width in the aromatic region (highlighted in pink here). After subsequent Trt cleavage with 95% TFA, the brush glycopolymer bears a terminal amine for further conjugation. The incorporation of **P11**, a side-chain brush glycopolymer with one mannose molecule per oligoamidoamine, necessitates that the triazole signal in brush polymer **B5** exhibits a corresponding proton count. When the integral sums of the aromatic compounds in both the side chain glycopolymers and the brush^2^ end are compared, the consistency of the proton number is verified.

**Figure 3:**
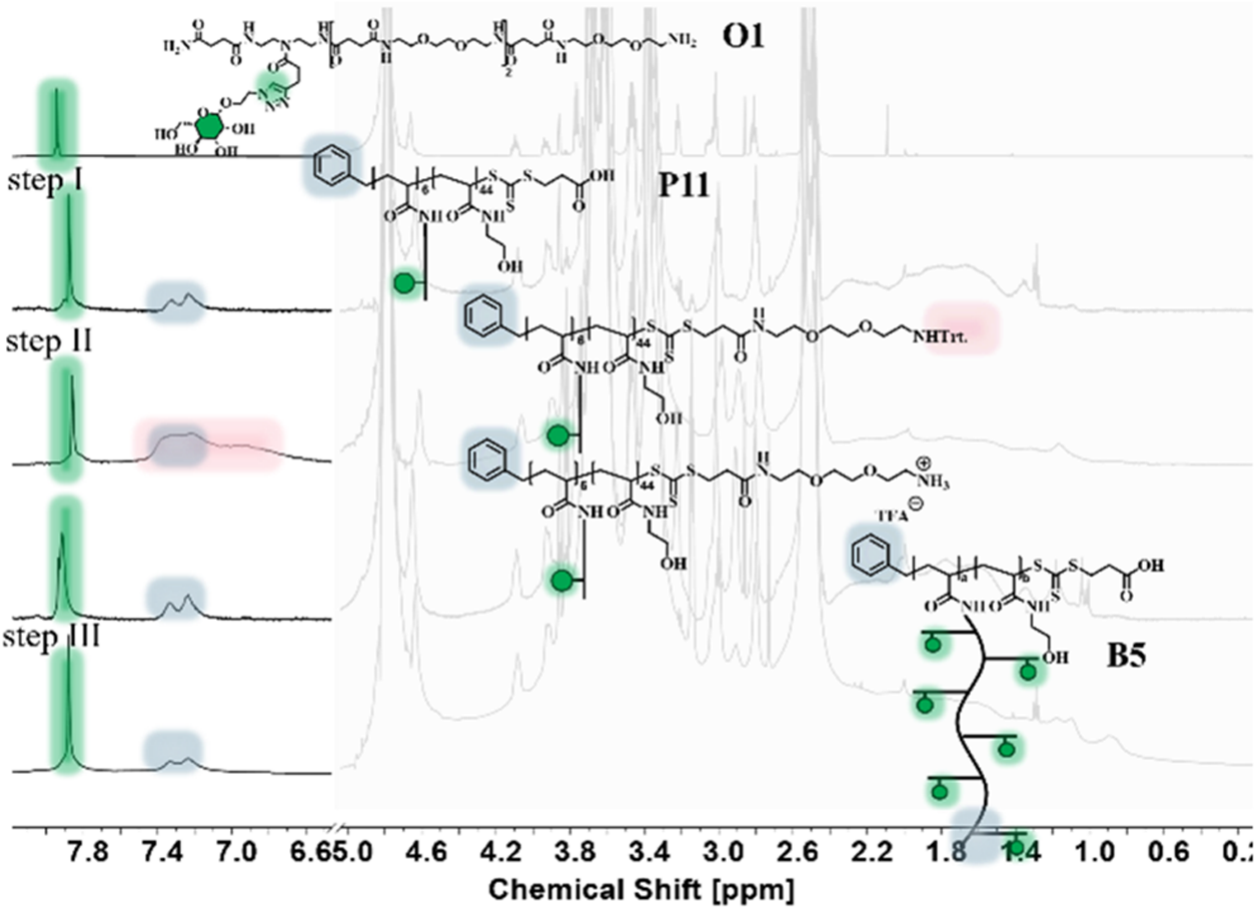
Comparison of the individual synthesis steps from oligoamidoamine to brush^2^. The characteristic triazole protons are marked in green, the aromatic protons of the RAFT initiator in blue, and the Trt protecting group in red. The backbone and main side chain signals are grayed out, as they only make a limited contribution to explaining the conversion step. These spectra are shown here only as examples and focus on the characteristic peaks to demonstrate the synthesis route. A more detailed analysis of the ^1^H-NMR spectra is shown in the Supporting Information.

To determine the efficiency of the sulfation reaction in obtaining sulfated brush^2^ glycopolymers via ^1^H-NMR, the chemical shift of the triazole proton before and after sulfation is considered (Supporting Information). Based on the sulfate groups, the change of the electrochemical environment (shielding) has the greatest impact on the chemical shift, toward a lower field, of the triazole protons and the anomeric center of mannose. Table 1 presents the final structures obtained, along with their respective yields. For comparison, nonglycosylated homopolymers—linear and brushed—are synthesized using exclusively ethanolamine or isopropylamine to functionalize the PFPA precursor. Additionally, single-brush glycopolymers with glycooligomer side chains presenting one or three mannose units are obtained by using the same high molecular weight PFPA precursor as for the brush^2^ glycopolymers (Table 1).

### Properties of Highly Branched Sulfated Glycopolymers as Mucin Mimetics

After the successful establishment of the synthetic protocol to derive a series of linear, single-brushed, brush^2^, and globally sulfated glycopolymers, we investigated their ability to mimic key features of natural mucins, such as hydration, conformation, and adhesion. Mucins have shown characteristic sacrificial layer formation on different substrates and so-called hydration lubrication. Although the hydrophobic folded regions of mucins are essential for the sacrificial layer formation, in particular on hydrophobic substrates,^12^ these domains are not within the scope of this study. We focus on mimicking the hydration, conformation, and adhesion properties of the glycosylated domains of mucins, which are crucial for the adhesion and thus sacrificial layer formation.^11,12^

Dynamic light scattering (DLS) and small-angle neutron scattering (SANS) experiments are carried out to obtain insights into the conformation and the hydration state of brush^2^ glycopolymers. From DLS, the hydrodynamic diameters of all brush^2^ (**B1**–**B17**) and control structures (**P15**–**P24**) are revealed, ranging from 66 to 184 nm (see Supporting Information for detailed information). These first experiments show that brush^2^ structures with longer brushed glycopolymer side chains consistently exhibit larger hydrodynamic diameters in comparison to those with shorter brushed glycopolymer side chains. This is independent of the type of quencher introduced during synthesis (ethanolamine or isopropylamine) and overall carbohydrate content. This supports the expectation that larger brushed glycopolymer side chains lead to increased hydrodynamic diameters and can even induce extended bottlebrush rather than random coil conformations.^47^ The brush^2^ structures with sulfation exhibit approximately similar size ranges for their hydrodynamic diameter as brushed heparan sulfate derivatives reported in the literature.^48^ For glycopolymers with a p­(NIPAM) backbone, we can also study the potential LCST behavior. Therefore, all samples containing p­(NIPAM) backbones are measured at 20 °C (below LCST) and 40 °C (above LCST), respectively. Glycopolymers exhibit larger hydrodynamic diameters above the LCST (40 °C). This increase is mainly due to the aggregation of polymer chains, driven by enhanced hydrophobic interactions and the transition from a solvated to a collapsed state. Such behavior is well-known for thermoresponsive polymers and is affected by their molecular architecture and environmental conditions.^49–51^ To gain further insights, selected polymers are additionally analyzed by SANS. Detailed information on the experimental conditions can be found in the Supporting Information. To further investigate the conformation differences in the polymer structures of differently brushed glycopolymers, experiments are conducted with a brush^2^ glycopolymer (**B6**) and a linear control glycopolymers (**P15**). These measurements allow us to resolve the individual polymer strands due to the chosen *q* range (Figure 4).

**Figure 4:**
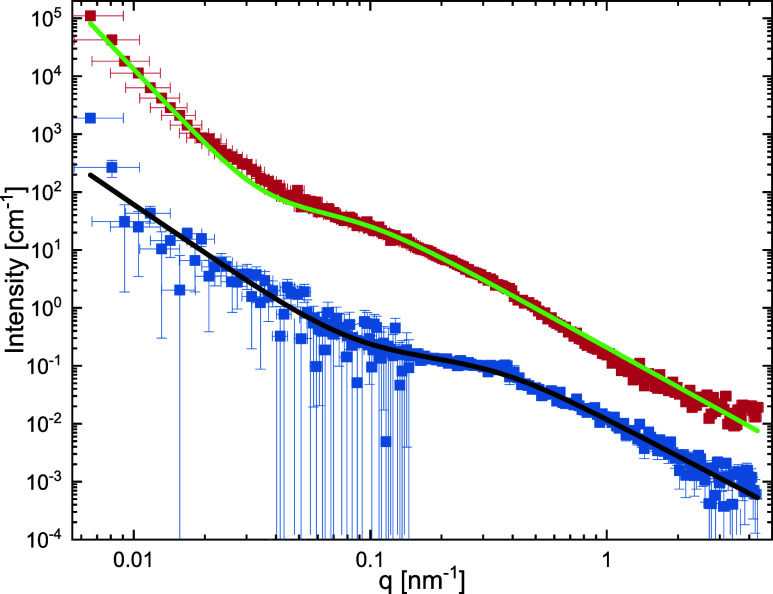
Radially averaged scattering profiles obtained from SANS measurements performed in D_2_O with 5 mg/mL at 25 °C. Red squares correspond to data recorded from sample **B6**, while blue squares correspond to sample **P15**. The profile of **B6** is offset vertically by multiplication by a factor of 10 for the sake of clarity. Solid lines correspond to fits to the data. The incoherent background scattering was subtracted.

Both scattering profiles can be described by three contributions: a *q*-independent incoherent background, a sharp increase in scattering intensities toward low *q*, which can be described by a power law, and a polymer form factor, which is accounted for by a generalized Gaussian coil model.^52^ The form factor is based on the random walk description of a polymer, as first described by Flory.^53,54^ This model considers the radius of gyration *R*_g_ of the polymer coil, the (excluded volume parameter) solvent parameter *v*, and the forward scattering of the neutrons. The compositions of the fits are shown in detail in the Supporting Information. The fits to the data, shown as solid lines in Figure 4, describe the experimental data well. Some small deviations arise for branched sample **B6** in the low *q* region. These deviations could arise from the occurrence of larger particle aggregates present in the sample lying just outside the *q* range. Both fits can be described with similar solvent parameters of *ν* = 0.44 (**B6**) and *ν* = 0.47 (**P15**), respectively. This parameter suggests that the interactions between polymer and solvent are slightly unfavorable. The steep increase below *q* = 0.05 nm^–1^ can be described by a power law, wherein the exponent is correlated to the solvent parameter *ν* as *n* = *1/ν* ≈ 2.2 for polymer coils.^55^ The coils have a radius of gyration *R*_g_ of 4.6 (**P15**) and 17.7 nm (**B6**), respectively. The increase in size with increasing molecular weight is in good agreement with theoretical calculations following the theory of Mark–Houwink-Kunh-Sakurada.^56^ Following this theory, the size of the polymer depends on the molecular weight through the equation *R*_g_ ∼ *M*_W_ *^ν^*. Thus, the DLS and SANS findings demonstrate size differences between conventional brushed and our newly introduced brush^2^ glycopolymers. For mucins, their high level of hydration behavior is attributed to their hydrophilic carbohydrate components.^36^ We find similar properties for our mucin mimetics, as we observe that the measured radii of gyration are considerably smaller than the radii of hydration, consistent with previous literature on mucin (e.g., MUC5AC: *R*_g_ = 187 nm, *R*_H_ > 1 μm).^57,58^ This indicates that these novel brush^2^ structures mimic natural mucins with respect to their conformational and hydration behavior.

Natural mucins form strongly adhering sacrificial layers on cellular substrates. The sacrificial layer mechanism depends on mucin molecules rapidly detaching and reattaching, linking their lubrication behavior to their adhesion characteristics.^5,8,9,59^ Also, their lubrication and protective properties are closely linked to their adhesion. We investigate in the following the adhesion properties of our mucin mimetics using selected samples via AFM-based single-molecule force spectroscopy (SMFS). Specifically, we examine the influence of mannose and sulfated mannose on the desorption (deadhesion) force of the here presented 2-hydroxyethyl acrylate (HEAA)-based glycopolymers. Using SMFS to investigate polymer interaction with solid substrates in liquid environment is well established,^60–67^ and has been successfully applied also for natural mucins.^12^

Single polymers are covalently attached to an AFM cantilever tip (see Supporting Information), using a functionalization protocol that has been established and successfully applied in multiple studies.^68–73^ The functionalized tip is then approached to a bare, freshly cleaved mica surface in water. After the polymer is brought into contact with the mica (dwell time: 1.0 s), the cantilever is retracted (usually at a velocity of 1.0 μm s^–1^) until the polymer desorbs from the surface (Figure 5a). We measure desorption forces at a defined pulling velocity, which allows us to compare different polymers, as previously done in SMFS experiments.^65,74,75^ Furthermore, we apply strict criteria to isolate single-molecule desorption events in force–extension curves. This includes the selection of characteristic force–extension curves, showing clear single-molecule desorption motifs.^76,77^ We observe single-molecule desorption events, such as plateaus of constant force (Figures 5b–d and S111) or desorption events represented by peaks, wherein the polymer is stretched between the AFM cantilever tip and the mica surface until detachment (Figures 5e–h and S111).^64,74,77,78^ We further performed control measurements (Figure S111) with purely poly­(ethylene glycol) (PEG) functionalized cantilevers to differentiate between force responses resulting from the investigated glycopolymers and from the PEG-linker system. It has been shown by Kienle et al.^65^ that plateaus are the result of surface-mobile polymers, independent of the polymer architecture, whereas peaks are due to surface-immobile polymers, which depend on the polymer architecture, in particular on the side chain structure.

**Figure 5:**
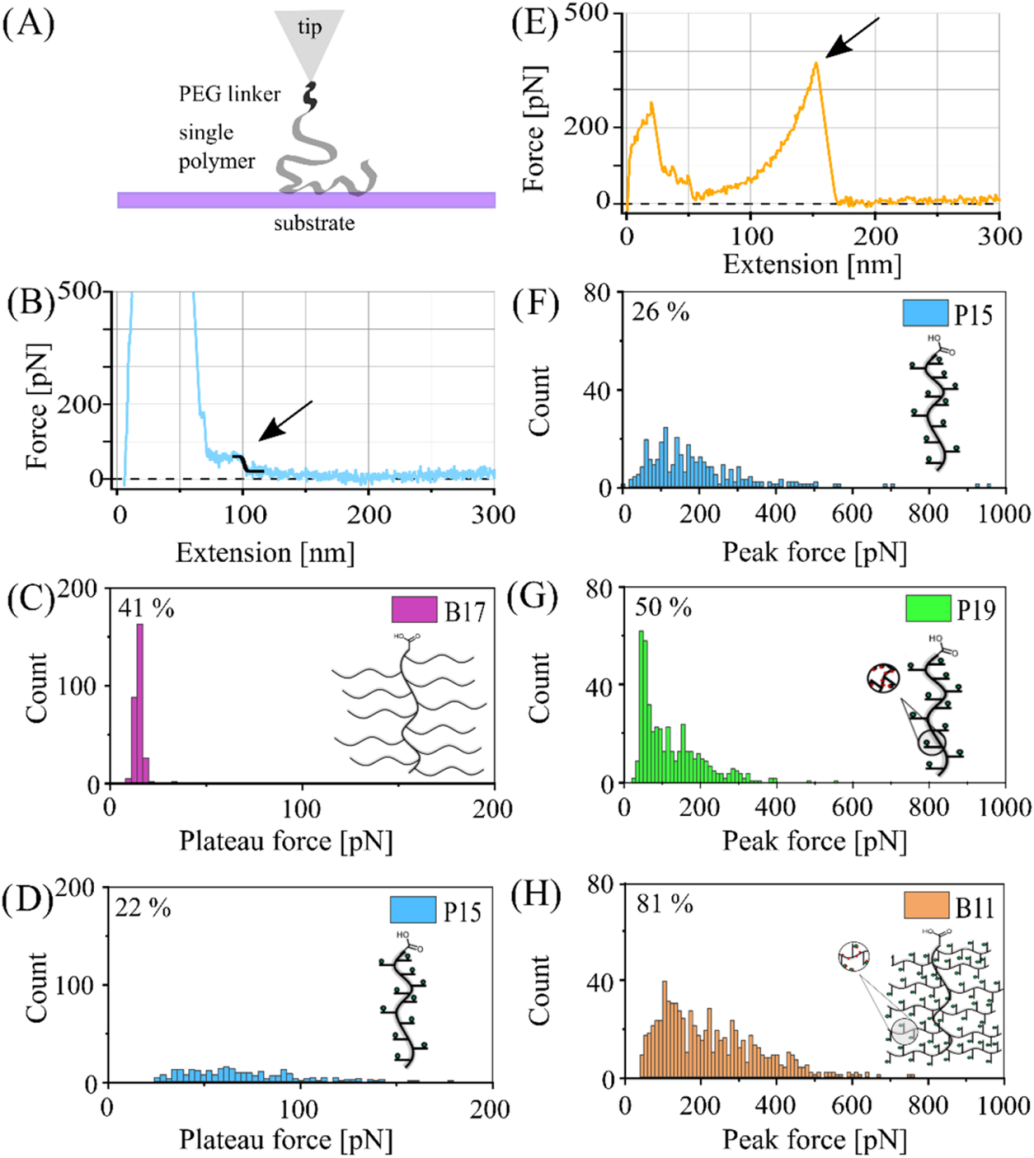
AFM-based single-molecule desorption. (A) Scheme of the covalent attachment of a single polymer to an AFM cantilever tip via EDC*NHS chemistry using poly­(ethylene glycol) (PEG) linkers and exemplary force–extension curves with (B) a plateau of constant force (for structure **P15**) or (E) with a force peak (for structure **B11**) as desorption motif, measured in water. The desorption motifs are indicated with arrows. Usually, approach and retraction velocities of 1.0 μm s^–1^, a dwell time toward the surface of 1.0 s, and a sampling rate of 5 kHz are taken. Adhesion peaks at small extensions (at the beginning of the retraction process) are due to the unspecific adhesion of the whole cantilever tip and the underlying substrate. (C,D) Plateau forces of **B17** (brush^2^, no carbohydrates, nonsulfated -41% of all obtained curves show single-molecule interaction events, 289/700 curves) and **P15** (linear, carbohydrates, nonsulfated -22% plateaus, 258/1200 curves). (F–H) Peak forces of **P15** (linear, carbohydrates, nonsulfated -26% peaks, 314/1200 curves), **P19** (linear, carbohydrates, sulfation -50% peaks, 447/900 curves), and **B11** (brush^2^, carbohydrates, sulfated -81% peaks, 731/900 curves).

Negative control brush^2^ polymer **B17**, without any mannose, shows exclusively plateaus of constant force as the desorption motif with a desorption force of only (15 ± 3) pN (Figure 5c). By contrast, brush polymer **P15** reveals a mix of plateaus and peaks as a desorption motif, with the plateaus exhibiting higher forces of around 100 pN (Figure 5d). The presence of plateaus is a sign of a high polymer mobility and dispersion forces acting between the polymer and mica. The occurrence of peaks is observed for all tested glycopolymer structures **P15**, **P19**, and **B11**. Their force–extension curves mainly exhibit peaks with peak forces up to 800 pN (Figure 5f–h). They all contain either mannose (nonsulfated glycopolymers e.g., **P15**) or statistically sulfated mannose (sulfated glycopolymers, e.g., **P19** and **B11**). This might lead to a decrease in mobility and a higher desorption force due to an increase of specific directional bonds. In particular, the polymer–substrate interaction is strengthened by the high density of hydroxyl in the mannose units, which increases the number of potential interaction sites and enables multivalent binding.^66^ This density can be varied and controlled through brush architecture and the introduction of carbohydrate motifs. When compared to brush^2^ polymer **B17** with no mannose, the potential hydrogen bond density (normalized based on the backbone length) increases slightly for brush glycopolymers **P15** and **P19**, by a number of $\rho_{P15,P19}=0.6\frac{\mathrm{H‐bonds}}{\mathrm{nm}}$ , reaching its maximum increase by $\rho_{B11}=3.6\frac{\mathrm{H‐bonds}}{\mathrm{nm}}$ for the brush^2^ glycopolymer **B11**. This increase of potential interaction sites reasonably explains not only the increase of the desorption force but also the increase of the interaction frequency for brush^2^ glycopolymer **B11** in comparison to the other glycopolymers (Figure 5h). Furthermore, in water, a hydration layer is present on mica, giving rise to a short-range repulsive force.^79^ This hydration force opposes adsorption. However, the energetic costs of removing this hydration layer from mica can be compensated by favorable polymer–substrate interactions such as H-bonding and entropy gain by release of the hydration layer,^60,80^ which is also feasible for the glycopolymers based on the presence of hydroxyl groups. Additionally, the hydroxyl groups in mannose are neutral but polar, leading to the possibility of dipole-charge interaction with the negatively charged mica. This is further facilitated by the HEAA backbone, adding a large polyamide domain with additional H-bonding sites to overcome hydration barriers for adsorption.^81^ For comparison, experiments at increased ionic strength in buffer (10 mM HEPES, 50 mM NaCl, pH 7.0) with **P15** and **B11** on mica showed no desorption events (Figure S112), suggesting blocking of H-bonding by HEPES and hydrated salt ions.^82^

Furthermore, by comparing brush glycopolymer **P15** and its sulfated counterpart **P19**, we can deduce the influences of the sulfates on the desorption behavior. Both polymers have the same structure, but **P19** contains statistically sulfated mannose, making it polyanionic. In general, mica in water is also polyanionic, therefore one would expect dominant ionic repulsion between sulfated structures and mica, unless multivalent cations such as Mg^2+^ or Ca^2+^were present.^73,83^ However, previous studies have shown that negatively charged carbohydrates^60^ or proteins^84^ can also adsorb on mica in the absence of multivalent cations. This could be explained by solvent water molecules promoting hydrogen bonds between the sulfates and mica, i.e., a water-mediated attractive interaction between like-charged motifs.^85–87^ In addition, local charge heterogeneity^88^ (neutral vs negatively charged portions) facilitates the formation of hydrogen bonds. This is similar to mucins, where a multivalent interaction of the polyanionic, glycosylated chain (e.g., via chelate formation, hydrogen bonding, or electrostatic interaction) has been observed on steel.^11^ Furthermore, positive counterions (Na^+^) could be present in our polymer solutions, a remnant from the synthesis process, where the sulfurtrioxide trimethylamine complex (TMA*SO_3_) for sulfation is quenched with sodium acetate. Such ions partially screen the sulfates, reducing long-range electrostatic repulsion. As the nonsulfated **P15** and sulfated **P19** reveal the same range of desorption forces, it is likely that their adhesion on a hydrophilic surface, such as mica, can be mainly attributed to the mannose units, while attractive and repulsive contributions of the sulfate groups might largely compensate each other.

To gain further insight into the interaction between the glycopolymers and the surface, selected structures are investigated in more detail with respect to their mica surface adsorbed conformations, in contrast to our previous investigations using DLS and SANS, which were performed in solution. The conformations of the presented polymers are influenced by their interaction with the mica surface, which, in turn, depends on the brush structure. Figures 6 and S113 show exemplary images of the polymers **B11**, **B17**, and **P19** analyzed by AFM-based imaging on mica (deposited in solution, dried, and imaged in air), showing compact conformations for brush glycopolymer **P19** and extended conformations for brush^2^ polymer **B11** (see Supporting Information, Figure S113 for more images). The AFM images further support an increase of polymer–substrate interactions for brush^2^ glycopolymers, corresponding to the increase in hydrogen bond density (see above). Brush^2^ polymer **B17** (Figure 6a), without any mannose, shows a high degree of self-aggregation, whereas the glycopolymers **P19** and **B11** (Figure 6b,c), containing mannose, form smaller or, in the case of **B11**, even linear aggregates, preferring mica substrate interaction over aggregation. We observe linear chains reaching several hundred nm for brush^2^ glycopolymer **B11**, which cannot correspond to a single molecule based on the molecular weight but is attributed to aggregation, where one molecule is connected to the next one forming a linear chain, possibly due to intermolecular hydrogen bonds. Such highly extended chain conformations on mica have been observed before for the linear, polyanionic polysaccharide hyaluronan.^89–93^ Here, the linear conformation might result from both the branched geometry of **B11** and the electrostatic repulsion of the sulfate groups, leading to both a rather extended chain conformation and to a larger interaction area with the mica substrate.

**Figure 6:**
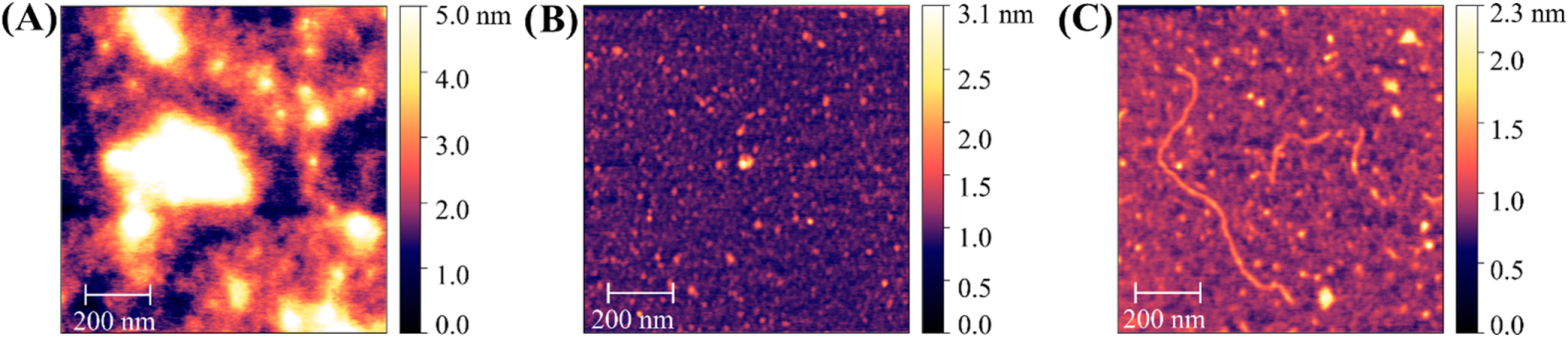
AFM topography images (size: 1 μm^2^) revealing different conformations of (A) brush^2^ glycopolymer **B17** without mannose, (B) brush glycopolymer **P19** with sulfated mannose, and (C) brush^2^ glycopolymer **B11** with sulfated mannose. All polymers are deposited from a 0.1 μg mL^–1^ polymer solution in water and then dried under vacuum. Images are obtained in air.

AFM topography images in Figures 6c and S113 support the SMFS data, where the brush^2^ polymer **B11** shows an extended and flat conformation on mica (deposited in solution, dried, and imaged in air). Thus, the contact area of **B11** is increased, which could also increase adhesion.^65,67^ This increase in contact area is most probably due to mannose: both **B11** and **B17** share the same branching topology and both are sulfated; however, **B17** does not carry any mannose and shows a coiled conformation (Figure 6a). Hence, mannose seems to increase not only the contact area but also the ability for multivalent binding.

## Conclusions

We introduce the synthesis of double-brushed glycopolymers as mucin mimetics by combining the solid-phase synthesis of sequence-defined glycooligomers and their conjugation onto polyactive ester scaffolds. First, we demonstrate the straightforward access to a library of linear, brushed, and double-brushed (or brush^2^) glycopolymers with controlled variations of structural parameters, such as branching, their brushed glycopolymer side chain length and number, carbohydrate content, and sulfation. Subsequently, selected structures are analyzed for their physicochemical properties, focusing on features that are characteristic of the unique behavior and function of natural mucins, such as their hydrodynamic size and adhesion properties. Specifically, we use DLS, SANS, AFM-based SMFS, and imaging to confirm that indeed brush^2^ glycopolymers successfully mimic key interaction parameters of mucins. We show that glycopolymer adhesion increases with branching sugar content, as it allows for multivalent binding due to hydrogen bonds, similar to the glycoprotein mucin, where multivalent interactions of the polyanionic, glycosylated chain are observed. AFM imaging reveals a highly linear conformation of sulfated brush^2^ glycopolymers, which we attribute to the combination of both the double-brush architecture as well as the presence of sulfated carbohydrate groups, as neither one of the nonmannose brush^2^ polymers of the simple brush glycopolymers shows a similar behavior. Indeed, such highly extended chain conformations are typical features of natural mucins. Thus, in summary, we have succeeded in synthesizing double-brushed glycopolymers as new mucin mimetics with mucin-specific characteristics. Future studies will extend the synthetic strategy to include further features of natural mucins, such as hydrophobic regions. We will also study additional biophysical properties such as their ability to retain water and act as a lubricant, and will explore potential applications of brush^2^ glycopolymers, e.g., in wound healing, mucosal barrier enhancement, and respiratory disease treatments, and to build increasingly complex glycocalyx models to study pathogen attachment.

## Supplementary Material



## Notes

Materials and methods; synthetic protocols; analysis of compounds via NMR; RP-HPLC; MALDI-Tof-MS; HR-ESI-MS; H_2_O-SEC-MALS; THF-SEC; elemental analysis; SANS; DLS; and AFM (PDF)
