# Supplementary material for: Highly Branched Sulfated Glycopolymers as Mucin Mimetics
Source: J Am Chem Soc. 2025 Aug 28;147(36):32698–709. doi: 10.1021/jacs.5c08232 (PMC12426936; doi:10.1021/jacs.5c08232)
Supplement: Supplementary file 1 [file ja5c08232_si_001.pdf]

Supporting Information

Highly branched sulfated glycopolymers as mucin mimetics

Melina I. Feldhof,†[a] Rebecca Schlatterer,†[b] Friederike Strahl,[b] Jonathan Garthe,[c] Sylvain Prévost,[d] Stephan Schmidt,[e] Matthias Karg,[c, f] Bizan N. Balzer,[b,e,g] Laura Hartmann*[g,h]*

[a] Department of Organic Chemistry and Macromolecular Chemistry, Heinrich-Heine-University Düsseldorf, Universitätsstraße 1, 40225 Düsseldorf, Germany
[b] Institute of Physical Chemistry, Albertstr. 21, 79104 Freiburg, Germany
[c] Institute of Physical Chemistry I, Heinrich-Heine-University Düsseldorf, Universitätsstraße 1, 40225 Düsseldorf, Germany
[d] Instrument responsible D11, Institute Max von Laue – Paul Langevin (ILL), 71, avenue des Martyrs – CS 20156, 38042 Grenoble cedex 9, France
[e] Cluster of Excellence livMatS @ FIT-Freiburg Center for Interactive Materials and Bioinspired Technologies, University of Freiburg, Georges-Köhler-Allee 105, 79110 Freiburg, Germany
[f] Physical Chemistry of Functional Polymers, Martin Luther University Halle-Wittenberg, Von-Danckelmann-Platz 4, 06120 Halle (Saale), Germany
[g] Freiburg Materials Research Center (FMF), University of Freiburg, Stefan-Meier-Str. 21, 79104 Freiburg, Germany
[h] Institute for Macromolecular Chemistry, University of Freiburg, Stefan-Meier-Str. 31, D-79104 Freiburg i.Br., Germany

†First authorship contributed equally
*Corresponding authors: Dr. Bizan N. Balzer, E-Mail: bizan.balzer@physchem.uni-freiburg.de; Prof. Dr. Laura Hartmann, E-mail: laura.hartmann@makro.uni-freiburg.de

Table of Contents

1. Experimental Parts S2
   1.1 Materials S2
   1.2 Instrumentation S2
   1.3 General Methods S4
2. Synthesis and analytical data S9
   2.1 Synthesis of PFPA (***S1***) and the bridge structure (***S2***) S9
   2.2 Synthesis of p(PFPA) and p(HEAA) for analytics S14
   2.3 Oligoamidoamide synthesis of **O1** and **O2** S25
   2.4 Synthesis of side chain glycopolymers **P7-P14** S33
   2.5 Synthesis of brush²-glycopolymers **B1-B16** S41
   2.6 Synthesis of reference structures **P15**–**P24** and **B17** S57
   2.7 DLS measurement S68
   2.8 SANS measurement S69
   2.9 AFM measurement S70
3. Literature S74

1. Experimental Parts

1.1 Materials

All chemicals and solvents were used without further purification and were purchased from commercial sources. Type I water, purified by Barnstead™ MicroPure™ ThermoFisher SCIENTIFIC ultrapure water system, was used unless otherwise mentioned.

Acros Organics: piperidine 99%, copper(II) sulfate 98%; *Biosolve Chemicals*: *N*,*N*-dimethylformamide (DMF) > 99.8%, for peptide synthesis; *Carbosynth*: benzotriazol-1-yloxytripyrrolidinophosphonium hexafluorophosphate (PyBOP); *Carl Roth*: *N*,*N*-diisopropylethylamine (DIPEA) ≥ 99%, *N*,*N*-dimethylformamide (DMF) > 99.8%, chloroform ≥ 99.9%, hexane ≥ 95%, for synthesis, triethylamine ≥ 99.5%; *Fisher Scientific*: 2,2′-(ethylenedioxy)bis(ethylamine) 97 +%, trifluoroacetic acid ≥ 99.5%, acetonitrile ≥ 99.9%, diethyl ether ≥ 99.8%, with BHT, acetone ≥ 99.8%, toluene ≥ 99.8%; *Merck*: acryloyl chloride 97%, benzene ≥ 99.8%, deuterium oxide ≥ 99.9%, sodium diethyldithiocarbamate ≥ 97%; *Nanocs*: triethoxysilane-polyethylene glycole (PEG)-methoxy 5 kDa; *RAPP Polymere*: TentaGel® S RAM resin (loading: 0.26 mmol/g); *Sigma Aldrich*: 2-(2-carboxyethylsulfanylthiocarbonylsulfanyl)propionic acid 95%, azobisisobutyronitrile 98%, dichloromethane ≥ 99.9%, ethanolamine ≥ 99%, sodium sulfate ≥ 99.0%, triisopropyl silane (TIPS) 98%, *TCIchemicals*: pentafluorophenol > 98%, trityl chloride > 98%; *VWR*: ethanol ≥ 99.9%.

1.2 Instrumentation

Nuclear Magnetic Resonance Spectroscopy (NMR):

^{1}H-NMR and ^{19}F-NMR spectra were measured with a Bruker Avance III 600 (600 MHz) at room temperature. ^{13}C-NMR spectra were measured with a Bruker Avance III 300 (300 MHz) at room temperature. Chemical shifts were reported in delta (δ) expressed in parts per million (ppm) for all NMR spectra. D_2O and $CDCl_3$ serve as the deuterated solvents for the spectra. Multiplicities were abbreviated as the following: singlet (s), doublet (d), triplet (t), quartet (q), multiplet (m). The assignment of carbohydrate signals on NMR followed the chronological enumeration of protons atoms within a monosaccharide starting from the reducing end.

Reversed Phase High Pressure Liquid Chromatography Mass Spectrometry (RP-HPLC-MS): RP-HPLC-MS measurements were performed on Agilent Technologies 1260 Infinity series coupled with Agilent quadrupole mass spectrometer with an Electrospray Ionization (ESI) source operating in a m/z range of 200 to 2000. Spectra were measured with A: 95% H_2O, 5% ACN, 0.1% formic acid and B: 5% H_2O, 95% ACN, 0.1% formic acid. Separation was performed at 25 °C using an Agilent MZ-Aqua Perfect C_{18} 3 µm (50 x 3.0 mm) column with a linear solvent gradient starting at 100% A, ending at 50% B in 17 min at a flow rate of 0.4 mL/min. Indicated purities were determined by integration of

the UV-signal detected by a wavelength detector set to 214 nm with the OpenLab ChemStation software for LC/MS from Agilent Technologies.

Matrix-assisted laser desorption/ionization with time-of-flight mass spectrometer (MALDI-TOF-MS): MALDI-TOF-MS spectra was performed using a Bruker Daltonics UltfelXtreme device. 2,5 dihydroxybenzoic acid (DHB) was the used matrix.

High-resolution-electrospray-ionisation mass-spectrometry (HR-ESI): HR-ESI-MS were performed on UHR-QTOF maXis 4G from Bruker Daltonics.

Dynamic light scattering (DLS): DLS measurements were performed at a Malvern HPPS 3.3 instrument (Malvern Panalytical, Kassel, Germany) equipped with a 633 nm He/Ne-laser with the detector set in backscattering configuration (171°). Protein-polymer conjugates (1-0.25 mg/mL) in 1 cm polystyrene cuvettes were analyzed at temperatures of 20°C or 40°C. For evaluation the autocorrection function, the decay constant and first cumulant were determined by exponential fits the and the hydrodynamic radii were calculated by the Stokes-Einstein relation.

Size Exclusion Chromatography─Multi-Angle Light Scattering (H_2O-SEC-MALS): SEC analysis was measured on an Agilent 1200 series HPLC system and three aqueous SEC columns provided by Polymer Standards Service (PSS). The columns were two Suprema Lux analytical columns (8 mm diameter and 5 µm particle size) and one precolumn (50 mm, 2 × 160 Å of 300 mm and 1000 Å of 300 mm). The eluents were buffer systems: (I) MilliQ water mixed with 30% acetonitrile, 50 mM, NaH_2PO_4, 150 mM NaCl, and 250 ppm NaN_3 with a pH = 7.0 (via addition of 50 mL of 3 molar aqueous sodium hydroxide solution) filtered with an inline 0.1 µm membrane and running at 0.8 mL per min, (II) PBS buffer, 5% NaN_3 at pH 7.4 with filtered with an inline 0.1 µm membrane and running at 1.0 mL per min. Multi-angle light scattering is recorded via miniDAWN TREOS and differential refractive index spectra with Optilab rEX both supplied by Wyatt Technologies EU. Data analysis was committed with Astra 5 software and a d*n*/d*c* value of 0.156 for each polymer.

Tetrahydrofuran-Size Exclusion Chromatography (THF-SEC): THF-SEC measurements were carried out with a Viscotek VE 3580 RI detector and a SYKAM S 3250 UV/Vis detector equipped with a polystyrene column (300 × 8.0 mm, 5 µm) and a polyacryl column (300 × 8.0 mm, 5 µm). A S5200 (SYKAM) sample injector as an auto sampler was utilized. THF was used as a solvent and toluene as a reference. The measurements were carried out with an injection volume of 100 µL and a flow rate of 1 mL/min. The molecular weights were determined with the Chromatographica (hs GmbH) software.

Elementar analysis: The ratios of carbon, hydrogen, nitrogen, and sulfur were determined using a Vario Micro Cube provided by Analysensysteme GmbH.

Small-Angle Neutron Scattering (SANS): SANS data were measured on D22 [DOI:10.5291/ILL-DATA.9-10-1845] at the Institut Laue-Langevin – The European Neutron Source, Grenoble, France. The front detector was positioned at 1.4 m from the sample at an angle of 20°, the rear detector was at 17.6 m, the beam was collimated at 17.6 m and the sample aperture was 7×10 (h×v) mm^2. One wavelength λ (relative full width at half maximum 10 %) was used: selecting λ =6.0 Å with a source aperture of 40×55 mm^2 (direct beam standard-deviation on detector 0.90 mrad) allowed covering a continuous q-range of 0.0024–0.66 $Å^{-1}$, where q is the magnitude of the wavevector ($q = (4\pi/\lambda) \times \sin(\Theta/2)$, Θ being the scattering angle) (flux of 1.67 MHz). Samples were kept in quartz cuvettes (type 100-QS and 110-QS, Hellma GmbH, Müllheim, Germany) of 1 mm pathway, on a thermalized rack with recorded temperature. Data were reduced with the program Grasp V.10.26h[1] normalizing with monitor, subtracting the contribution from the empty cell, taking into account noise from the measurement with a sintered $^{10}B_4C$ piece at the sample position, and using for transmission the intensity transmitted by a semi-transparent beam stop. Parallax from the detector and from the sample attenuation were corrected for, and a flat field was used. Absolute scale was obtained from the measurement of the direct beam with a calibrated attenuator.

1.3 General Methods

Standard protocols for solid phase synthesis: TentaGel resin was utilized in the synthesis process of both oligoamidoamines and the building block assembly, which involved iterative repetition of deprotection and conjugation steps. The backbone sequences were finished by Fmoc deprotection of the terminal amine enabling the conjugation to the active ester polymers. The required methods are described in the following section and can performed either by manual or automated synthesis (CS136XT, CS Bio). The given equivalents are related to 0.1 mmol scale.

Swelling protocol: Before the first cleavage of the N-Fmoc protecting group, the resin was swollen twice with approx. 5 mL DCM and then reconditioned 10 times to DMF.

Fmoc-Deprotection: *N*-Fmoc protecting groups of the functionalized TentaGel resin or the immobilized building blocks were removed by incubating approx. 5 ml 25% piperidine solution in DMF, shaken for 15 minutes and washed five times with DMF. The deprotection step was repeated and the resin was then washed with ten times with DMF.

Building block assembly: The two *N*-Fmoc-protected building blocks used here, **EDS** and **TDS**, were assembled on the solid support of the sequence-defined oligoamidoamines. After the respective Fmoc deprotection of the N-terminus, the next building block was conjugated using amide coupling methods. For this purpose, a solution of the **TDS** or **EDS** building block (5.0 eq.), PyBOP (4.9 eq.) and DIPEA (10 eq.) was dissolved in 2 mL DMF and added to the reaction vessel. The reaction mixture was shaken for 60 minutes at room temperature (20 °C) and then the resin was washed 10 times with 5 mL DMF each time.

Copper(I)-mediated azide-alkyne cycloaddition (CuAAC): After complete assembly of the sequence, the azide-functionalized mannose was conjugated to the alkyne-functional groups of the **TDS** building block of the macromolecule. For this purpose, copper(I)-mediated azide-alkyne cycloaddition (CuAAC) was performed by subsequent addition of N-methyloxyamine solution (2.5 eq. per alkyne group, solution in 100 µl DMF), copper(II) sulfate (2.5 eq. per alkyne group, solution in 100 µl water) and sodium ascorbate (2.0 eq. per alkyne group, solution in 100 µl water). The reaction vessel was covered with aluminum foil to protect it from light and shaken at room temperature for 18 h. The resin was then washed with the following wash cycle and repeated until it had regained its original yellow color: 5 ml of solution was used each time, 3 x 23 mM DDC solution (in DMF/water, *v/v*, 1:1, sodium diethyldithiocarbamate), 5 x DMF, 3 x water, 2 x DMF, 3 x DCM, 2 x DMF.

Deacetylation: The glycooligomers underwent deacetylation using Zemplén conditions.[2] Initially, the resin was washed five times with methanol. Subsequently, 5 mL of a 0.2 M NaOMe solution in MeOH was drawn into the reactor. The resin was agitated for 30 minutes before discarding the solution. Following this, the resin was washed twice with MeOH. Then, an additional 5 mL of the 0.2 M NaOMe was pulled into the reactor. After another 30 minutes, the solution was again discarded, and the resin was washed five times with MeOH.

***Cleavage reaction*:** The synthesized macromolecules were cleaved from the resin by adding 2 ml of TFA/TIPS/DCM (95/2.5/2.5) solution to the reaction vessel and shaking for 30 minutes, precipitated in cold diethyl ether and freeze-dried.

Synthesis of brush glycopolymers and linear reference structures:The corresponding p(PFPA) (***P1-3***) was dissolved in DMF (50 mg/mL). The amount of the corresponding oligoamidoamine was dissolved in 500 µL DMF and added to the active ester polymer solution. Then 20 eq. triethylamine (based on the amine) was added and the reaction and stirred at 40 °C for approx. 18 h, before 20 eq. ethanolamine or isopropylamine (based on the active ester repeating unit) was added and stirred at 40 °C for a further 18 h. The fully quenched polymer was then precipitated in cold acetone (for ethanolamine conversion) or cold diethyl ether (for isopropylamine conversion) for 2 h at -20 °C, centrifuged, purified by dialysis (cut off: 1 kDa) and lyophilized. In the reference samples, the functionalization with **O1** or **O2** was not required, whereby the active ester polymer was reacted directly with the 20 eq. ethanolamine or isoproplamine over 18 h at 40 °C.

Attachment of the bridge molecule to glycopolymers: The bridge molecule was attached via EDC-NHS chemistry. The corresponding arm polymer (***P7-P14***) was dissolved in 700 µL DMF and pre-activated with 10 eq. EDC*HCL (dissolved in 100 µL DMF/water (*v/v* 1:1, 15 minutes at room temperature) before 10 eq. NHS (dissolved in 100 µL DMF, 15 minutes at room temperature, 20 °C) forms the corresponding active ester. Subsequently, 10 eq. of the bridging molecule (dissolved in

100 µL DMF) were added and the mixture was stirred for 18 h at room temperature before the polymer was purified and lyophilized by dialysis (cut off: 1 kDa).

Cleavage of the protecting group of the conjugated bridge molecule: In order to cleave off the triphenylmethyl protecting group, the corresponding polymer was dissolved in 200 µL DMF and suspended with 1 mL DCM. This was followed by 10 eq. triisopropylsilane and 2 mL trifluoroacetic acid were added and the suspension was stirred for one hour at room temperature (20 °C), whereby the suspension dissolved after ten minutes. The polymer was precipitated in diethyl ether, centrifuged and lyophilized.

Attachment of the amine-activated glycopolymers to polymer scaffold to gain brush² structures: The corresponding amount of p(PFPA) ***P3*** was dissolved in 200 µL DMF. The appropriate amine preactivated arm polymer was dissolved in 800 µL DMF and set to alkaline (pH~10) with 200 eq. triethylamine and allowed to swell for one hour before it was added to the active ester polymer and the reaction was stirred for 18 h at 40 °C. Subsequently, 80 eq. ethanolamine was added and stirred for another 18 h at 40 °C before the brush² polymer was precipitated in cold acetone, centrifuged off and purified and lyophilized via dialysis (cut off: 50 kDa).

Standard protocol for global sulfation: The corresponding brush-like glycopolymer were dissolved in 5-7 mL dry DMF before 40 Eq. TMA*SO_3 per OH-group were added. The mixture was stirred for 24h at 70 °C. After cooling to room temperature, the mixture was added in an aqueous sodium acetate solution (10wt.%) at 0 °C. The DMF/water mixture was evaporated under reduced pressure before dialysis (cut off: 50 kDa) and followed by lyophilization.

Cantilever functionalization for AFM-Based Single Molecule Force Spectroscopy: The corresponding structures were covalently attached to Si_3N_4 AFM cantilevers tips (MLCT-BIO-DC, Bruker Nano). After removal from the gel pad box, the cantilever chips were stored in toluene (≥ 99,8 %, Thermo Fisher Scientific,) for 15 min to dissolve any remaining contaminants. Then, they were dried and placed in the evacuated reaction chamber of a plasma system (Tetra 30 LF PC, Diener electronic) at 0.1 mbar. There, they were exposed to oxygen plasma for 4 min at a power of 20 % (120 W) and then quickly transferred from the plasma chamber and directly incubated in a 50:1 solution of 1.25 mg mL^{-1} triethoxysilane-PEG-methoxy (5 kDa, Nanocs) solved in dry toluene (≥ 99,5 %, ≤ 30 ppm water, Th. Geyer) and 1.25 mg mL^{-1} triethoxysilane-PEG-amine dissolved in dry chloroform (≥ 99,9 %, Carl Roth) at 60°C for 1 hour. Afterwards, they were rinsed twice in toluene twice in ethanol (≥ 99,9 %, VWR), and once in N,N-Dimethylmethanamid (DMF, ≥ 99,8 %, Carl Roth). Then, the cantilever chips were incubated in the respective polymer solution at 4°C over night.

Solutions of polymers were prepared by dissolving the polymers in 100 µL DMF and 100 µL ultrapure water if they contained charges, otherwise they were dissolved in 200 µL DMF. As they all

contain a unique terminal carboxylic end group, coupling to the amine-functionalized cantilever tip was carried out by EDC/NHS coupling. Thus, N-(3-Dimethylaminopropyl)-N′-ethyl-carbodiimid-hydrochlorid was dissolved in 50 µL DMF and 50 µL ultrapure water and then added to the polymer solution and stirred for 10 min. Next, N-Hydroxysuccinimide was dissolved in 50 µL DMF and then added to the polymer solution, already containing EDC*HCL, and stirred for another 10 min. Then, the polymer solution was diluted to obtain a concentration of 0.05-0.20 mg mL^{-1}, depending on the respective structure (see Table 2). The next day, the functionalized cantilever chips were rinsed in ultrapure water and mounted to the AFM for measurements in ultrapure water.

AFM-Based Force Spectroscopy: Force spectroscopy measurements were performed with a MFP3D (Asylum Research, an Oxford Instruments company) using a closed fluid cell at approx. 22°C. All measurements were performed on freshly cleaved mica (muscovite, diameter 10–12 mm, Plano). Mica was glued onto the AFM fluid cell using the high-resolution replicating compound 101RF (Microset Products). After immersing a functionalized AFM cantilever chip into solution, an equilibration time of 20 to 30 mins was used, after which the cantilever deflection remained stable. For calibration, the inverse optical lever sensitivity (invOLS) was determined by indentation into the underlying hard surface and averaging of five values at the beginning of any experiment. The force constant was obtained by the thermal noise method,[3] see Table 1.

A trigger point of up to 1.8 nN (50 nm deflection) was used and the dwell time was set to 1.0 s. Approach and retraction velocities were usually kept constant at 1.0 µm s^{-1} with a sampling rate of 5 kHz. Force curves were obtained by contacting the underlying surface in a grid-like fashion (force map). Therefore, force maps with 10 by 10 points on an area of 10 µm by 10 µm were taken, resulting in 100 force curves per force map. Thus, the AFM cantilever tip contacted the surface once within a square-like area of 1 µm^2.

Table 1. Inverse optical lever sensitivity (invOLS) and force constant k obtained from the cantilever calibration using thermal noise method.[3] c_m indicates the mass concentration used for the cantilever functionalization described in the experimental section.

**Sample**	**c_m / mg mL^{-1}**	**invOLS / nm V^{-1}**	***k* / pN nm^{-1}**
**2**	0.20	420	8
**3**	0.10	140	66
**4**	0.05	253	12
**6**	0.05	434	26

Control experiments with PEG: A trigger force of up to 1.8 nN (50 nm deflection) and a dwell time of 1.0 s were applied. The approach and retraction velocities were usually kept constant at 1.0 µm s^{-1} with a sampling rate of 5 kHz, taking force maps (10 by 10 points, 10 µm by 10 µm).

AFM Data Evaluation: Self-programmed procedures based on Igor Pro (Version 8, Wavemetric) were used to evaluate the force-extension curves with respect to desorption peaks. The desorption forces and extensions of plateaus of constant force were determined by fitting a sigmoidal function to the end of the plateaus.

The desorption peak evaluation was done by selecting the force and position of every last peak, wherein each peak corresponded to the breakage of an interaction of a single molecule or a portion thereof with the underlying surface. Absolute force values were subject to the uncertainty of cantilever spring constant calibration. The absolute force error amounted to around 10 %, while the relative uncertainties are about 2 %, when one and the same cantilever is used, as argued previously by *Pirzer et al.*[4] The desorption position error resulted from the standard deviation of the respective desorption position distribution.

AFM-Based Imaging: AFM images were acquired by a Cypher ES (Asylum Research, an Oxford Instruments company) at ca. 22°C on mica (muscovite, diameter 10–12 mm, Plano). The images were obtained using Scout 70 RAu (NuNano) cantilevers (nominal cantilever tip radius: 5 nm) with a resonance frequency of 70 kHz. AC mode imaging in air was performed with the blueDrive (photothermal excitation). A scan rate of 2.44 Hz and a scan angle of 0° (i.e., parallel to cantilever axis) were chosen. The mica surface was cleaved with a transparent adhesive tape until a flat surface was obtained; then, 100 µL of a solution containing the respective polymer in ultrapure water with a concentration of 0.1 µg mL^{-1} was applied and immediately placed in a desiccator to dry under vacuum for at least 2.5 h at ca. 22°C.

The acquired height images were analyzed with the Gwyddion Free SPM analysis software (ver. 2.59).[5] AFM topography images were evaluated by applying a mean plane subtraction, row alignment (medium of difference) and a correction of horizontal scars. The minimum values were shifted to zero. Wherever useful (e.g., for particularly large globules), masking was used prior to image correction to prevent correction artifacts. Note that each AFM images provide local information about the respective sample.

2. Synthesis and analytical data

Building blocks **EDS** (**e**thylene glycol **d**iamine **s**uccinyl, 1-(9H-fluoren-9-yl)-3,14-dioxo-2,7,10-trioxa-4,13-diazaheptadecan-17-oic acid), **TDS** (**t**riple **b**ond diethylenetriamine **s**uccinyl, 1-(fluorenyl)-3,11-dioxo-7-(pent-4-ynoyl)-2-oxa-4,7,10-triazatetra-decan-14-oic acid) and **α-D-mannose azid** were synthesized as previously reported.[6-8] p(PFPA) with Pn ~ 98 and Đ = 1.28 were synthesized as previously reported.[9]

2.1 Synthesis of PFPA (*S1*) and the bridge structure (*S2*)

S1 – PFPA

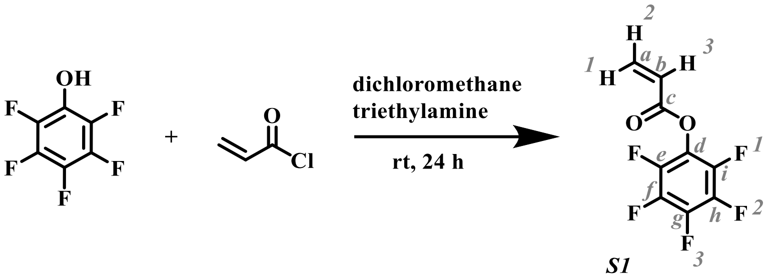

S1

The synthesis of pentafluorophenyl acrylate was carried out based on Graisuwan et al.[10] 10.38 g (56.40 mmol) pentafluorophenol was dissolved in 70 mL DCM, mixed with 9 mL triethylamine and cooled to 0 °C. Then 5.7 mL (70.53 mmol) acryloyl chloride was slowly added and kept for 24 h at room temperature. The reaction suspension was filtered, and the filtrate was washed twice with aqueous hydrochloric acid solution (pH 2). The organic phase was then washed twice with saturated sodium bicarbonate solution and twice more with distilled water. The organic phase was dried over sodium sulfate, filtered and the solvent removed on a rotary evaporator. A clear, light yellow, liquid product was obtained, which was purified by flash chromatography using a 120 g silica column and hexane as running agent. The product is a colorless solution.

Yield: 9.9 g (41.58 mmol; 74%)

^{1}H-NMR (600 MHz, Chloroform-*d*) δ (ppm) 6.75 – 6.69 (dt, *J* = 17.32, 0.91 Hz, 1H, *H1*), 6.41 – 6.31 (ddd, *J* = 17.32, 10.62, 0.97 Hz, 1H, *H3*), 6.21 – 6.15 (dt, *J* = 10.59, 0.92 Hz, 1H, *H2*).

^{13}C-NMR (300 MHz, Chloroform-*d*) δ (ppm) 161.83 (s, *Cc*), 143.00 (m, *Ci*), 141.37 (m, *Ce*), 139.75 (m, *Cg*), 138.01 (m, *Cf*), 136.37 (m, *Ch*), 135.64 (s, *Cb*), 125.50 (s, *Ca*), 125.09 (m, *Cd*).

^{19}F-NMR (564 MHz, Chloroform-*d*) δ (ppm) -152.50 – -152.63 (m, *F1*), -157.9 – -1157.99 (m, *F3*), -162.27 – -162.40 (m, *F2*).

LC-MS m/z calculated for $C_9H_3F_5O_2$ [M + ACN + H]$^+$ 280.03, found 280.00, [M + 2ACN + H]$^+$ 321.06 found 320.0, determined relative purity: > 95%

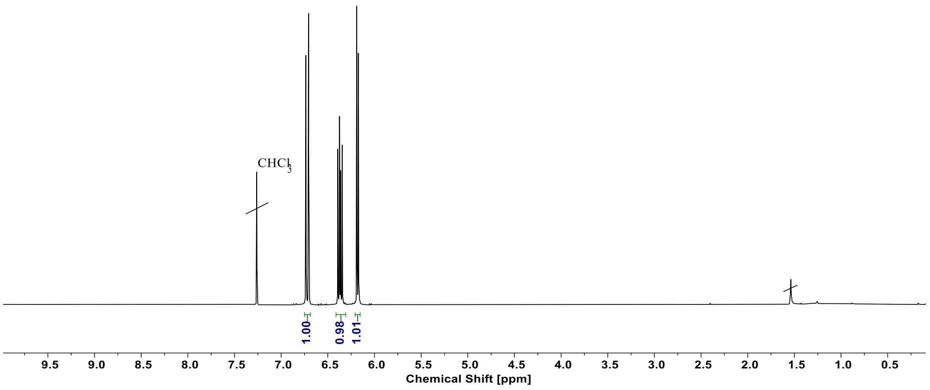

*Figure S1: ^{1}H-NMR spectra of PFPA (**S1**) (600 MHz, $CDCl_3$).*

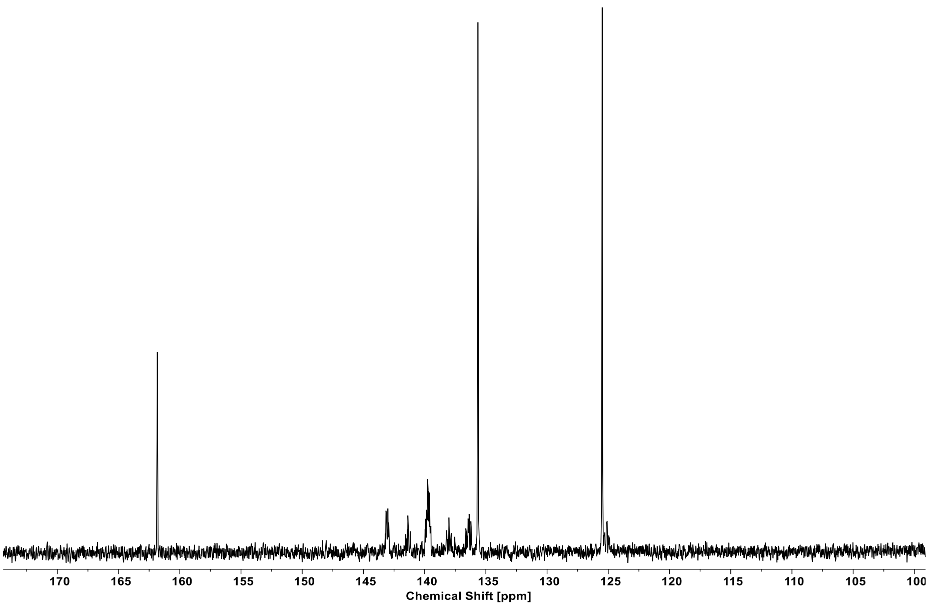

*Figure S2: ^{13}C-NMR spectra of PFPA (**S1**) (300 MHz, $CDCl_3$).*

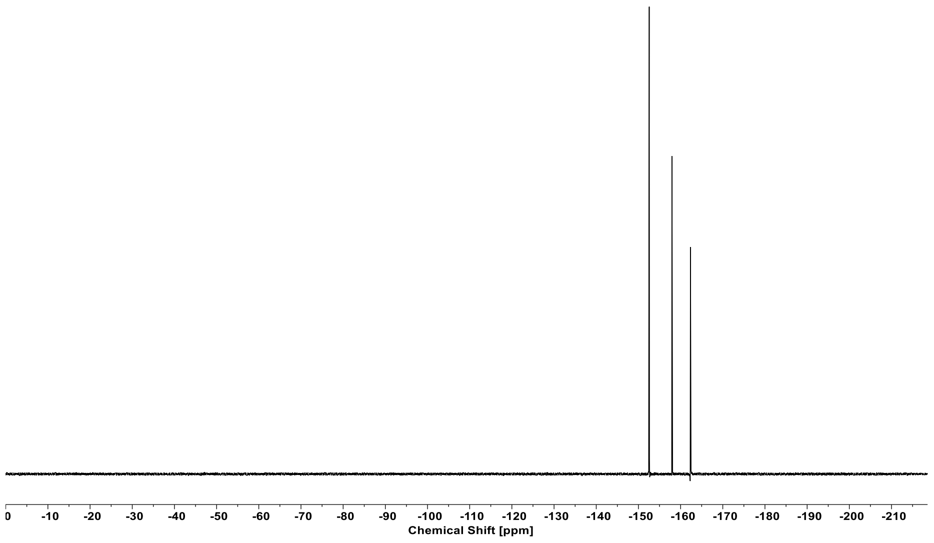

*Figure S3: ^{19}F-NMR spectra of PFPA (**S1**) (600 MHz,$CDCl_3$).*

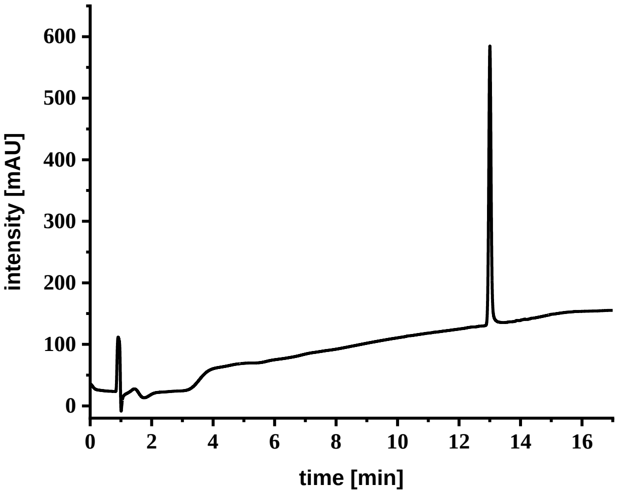

*Figure S4:RP-HPLC- chromatogram of PFPA (**S1**) (gradient of 5 to 95 vol% acetonitrile/water with 0.1 vol% formic acid, run time: 17 min).*

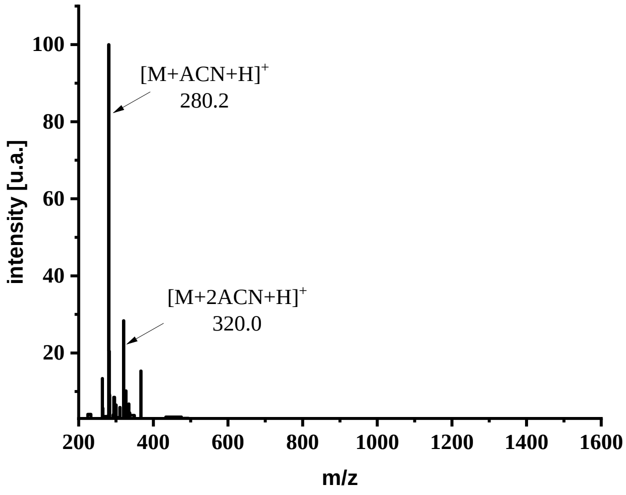

*Figure S5: ESI-MS chromatogram of PFPA (**S1**) at t = 13.00 min (gradient of 5 to 95 vol.% acetonitrile/water with 0.1 vol.% formic acid, run time: 17 min).*

S2 – bridge molecule

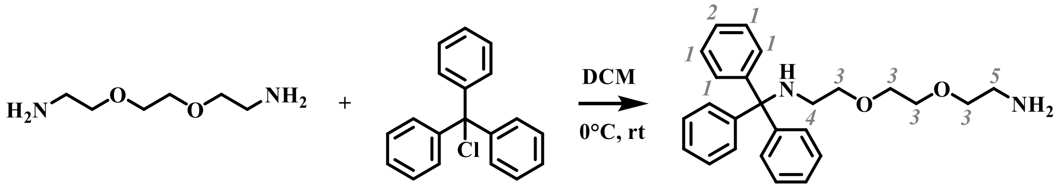

29.2 g (200.00 mmol) of 2,2′-(ethylenedioxy)bis(ethylamine) was dissolved in 500 mL of DCM and the solution was cooled to 0 °C in an ice bath. 13.94 g (50 mmol) of trityl chloride was dissolved in 300 mL of DCM and added over a period of about one hour and allowed to warm to room temperature overnight with stirring. The volume of solvent was halved on the rotary evaporator and washed three times with 300 mL of saturated sodium hydrogen carbonate solution each time. The organic phase was dried over magnesium sulphate and the solvent was removed under reduced pressure, leaving the product as a colorless syrup.

Yield: 18.18 g (46.55 mmol; 93%)

^{1}H-NMR (600 MHz, Chloroform-*d*): δ (ppm) 7.50 - 7.45 (dt, ^3J = 8.62 Hz, ^4J = 1.50 Hz, 6H, *H1*), 7.28 – 7.23 (m, 6H, *H1*), 7.19 – 7.15 (tt, ^3J= 7.34 Hz, ^4J= 1.30 Hz, 3H, *H2*), 3.62-3.46 (m, 8H, *H3*), 2.87 – 2.77 (t, ^3J = 5.20 Hz, 2H, *H4*), 2.38 – 2.33 (t, ^3J= 5.40 Hz, 2H, *H5*).

LC-MS m/z calculated for $C_{25}H_{30}N_2O_2$ $[M + Na]^+$ 413.23, found 413.2, $[M +Na]^+$, determined relative purity: 94%

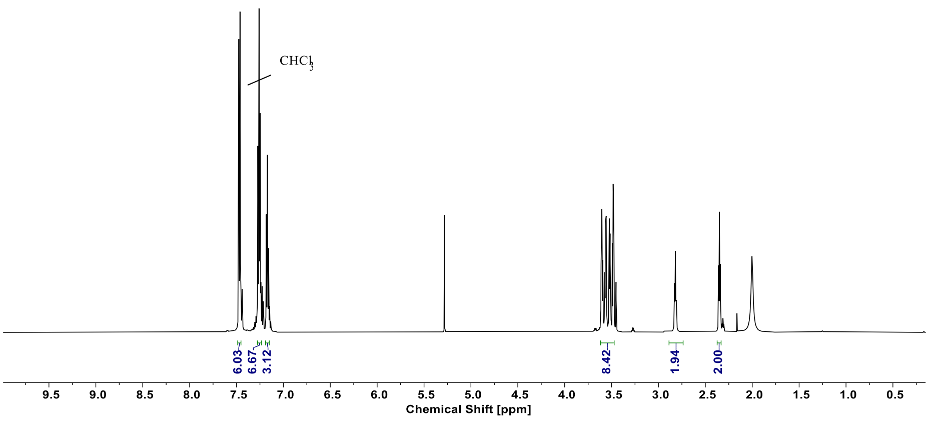

*Figure S6: ^{1}H-NMR spectra of bridge (**S2**) (600 MHz, CDCl3).*

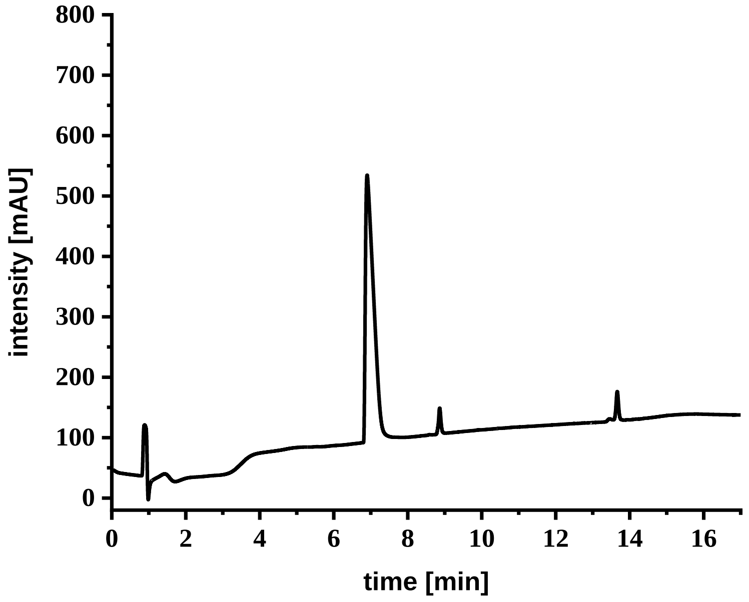

*Figure S7: RP-HPLC- chromatogram of bridge structure (**S2**) (gradient of 5 to 95 vol% acetonitrile/water with 0.1 vol% formic acid, run time: 17 min).*

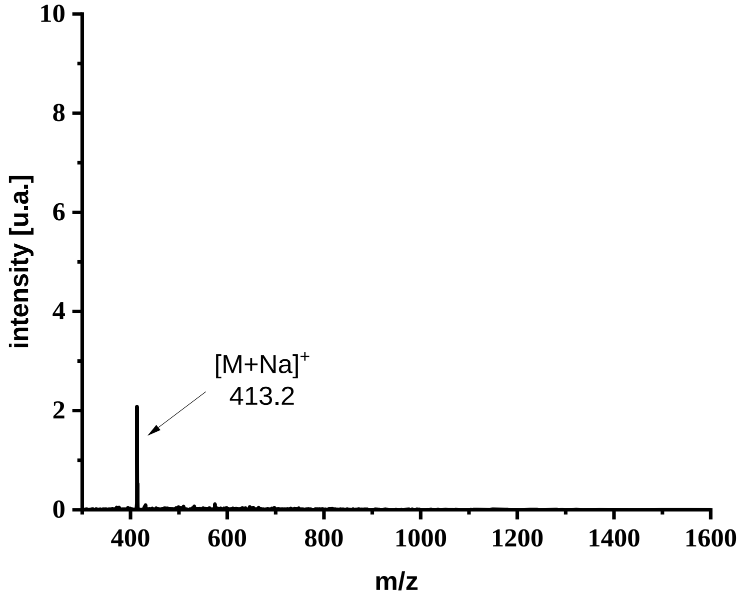

*Figure S8: ESI-MS chromatogram of PFPA (**S1**) at t = 6.89 min (gradient of 5 to 95 vol.% acetonitrile/water with 0.1 vol.% formic acid, run time: 17 min).*

2.2 Synthesis of p(PFPA) and p(HEAA) for analytics

P1-3 – p(PFPA)

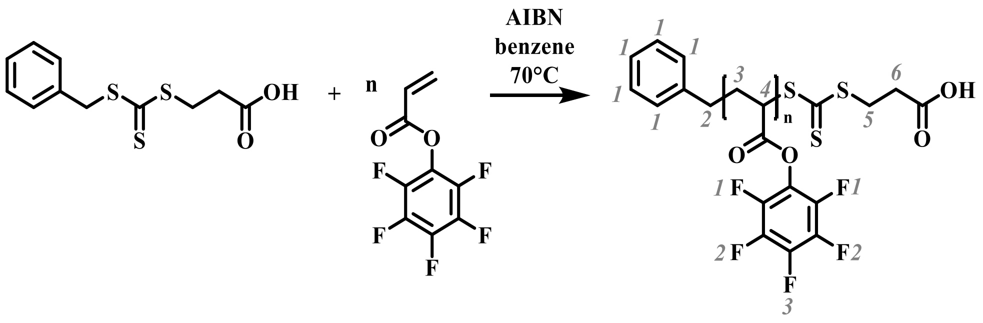

According to Fadi Shamout[11] synthesis procedure, **A** mg (**B** mmol) of azobisisobutyronitrile (AIBN) and **C** mg (**D** mmol) of 2-(2-carboxyethylsulfanylthiocarbonylsulfanyl)propionic acid (RAFT reagent) were dissolved in 8 mL of benzene and cooled in an ice bath. **E** mL of the monomer pentafluorophenyl acrylate (***S1***) (**F** mmol) was added and everything was rinsed under argon for 30 min. The reaction solution was heated to 70 °C for 24 hours. The polymerization was stopped by cooling with liquid nitrogen and under the influence of oxygen. The product was precipitated in 30 mL cold hexane and was centrifuged. The yellowish solid obtained was dissolved again with 2 mL benzene, precipitated a second time in cold hexane, washed twice more with 30 mL hexane, centrifuged and the polymer was dried under nitrogen.

Table 1: Summary of RAFT polymerization parameters for the synthesis of p(PFPA) P1-3.

polymer (*P*)	AIBN		RAFT reagent		monomer		Yield	
	A [mg]	B [mmol]	C [mg]	D [mmol]	E [mL]	F [mmol]	[mg]	[%]
P1	32.8	0.2	272.4	1	5.1	35	7.45	89
P2	23.0	0.14	190.7	0.7	5.1	35	7.61	91
P3	5.8	0.04	47.7	0.2	5.1	35	7.05	85

P1 – p(PFPA)

^{1}H-NMR (600 MHz, Chloroform-*d*) δ (ppm) 7.24 – 7.12 (d_{br}, J = 7.2 Hz, 5H, *H1*), 3.63 (s_{br}, 2H, *H5*), 3.23 – 0.58 (*H2 - H4, H6*). Pn ~ 46

^{19}F-NMR (564 MHz, Chloroform-*d*) δ (ppm) -153.15 (s, *F1*), -156.73 (s, *F3*), -162.20 (s, *F2*).

GPC (THF, toluene standard) *UV detector:* M_n = 6033 g/mol, M_w = 7602 g/mol, Đ = 1.26, *RI detector:* M_n = 5722 g/mol, M_w = 7185 g/mol, Đ = 1.26.

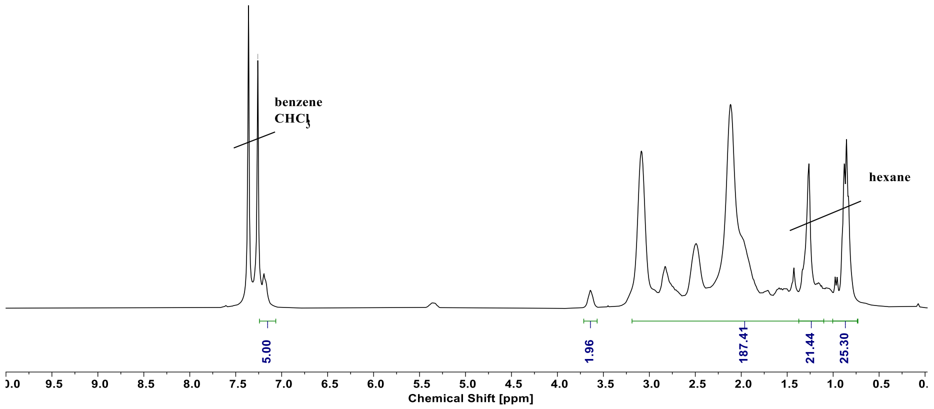

*Figure S9: ^{1}H-NMR spectra of p(PFPA) (**P1**) (600 MHz, $CDCl_3$).*

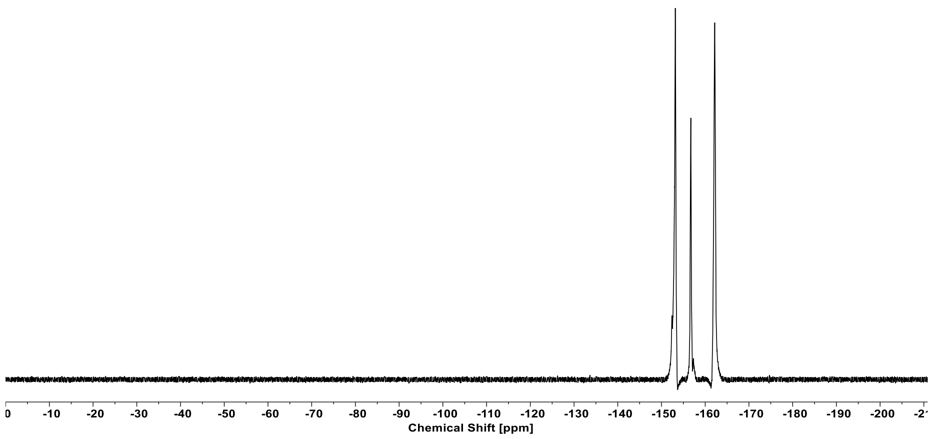

*Figure S10. ^{19}F-NMR spectra of p(PFPA) (**P1**) (600 MHz, $CDCl_3$).*

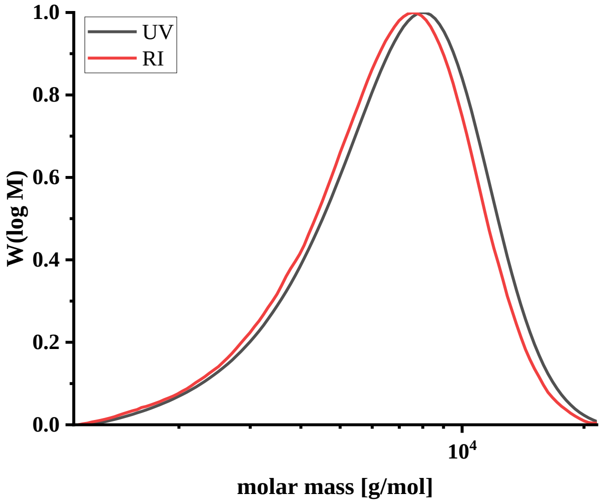

*Figure S11: GPC measurement of p(PFPA) **P1**, eluent: THF.*

P2 – p(PFPA)

^{1}H-NMR (600 MHz, Chloroform-*d*) δ (ppm) 7.20 – 7.11 (d_{br}, J = 7.7 Hz, 5H, *H1*), 3.24 – 1.20 (*H2 – H6*). Pn ~ 63

^{19}F-NMR (564 MHz, Chloroform-*d*) δ (ppm) -153.18 (s, *F1*), -156.70 (s, *F3*), -162.16 (s, *F2*).

GPC (THF, toluene standard) *UV detector:* M_n = 7372 g/mol, M_w = 9537 g/mol, Đ = 1.29, *RI detector:* M_n = 7079 g/mol, M_w = 9071 g/mol, Đ = 1.28.

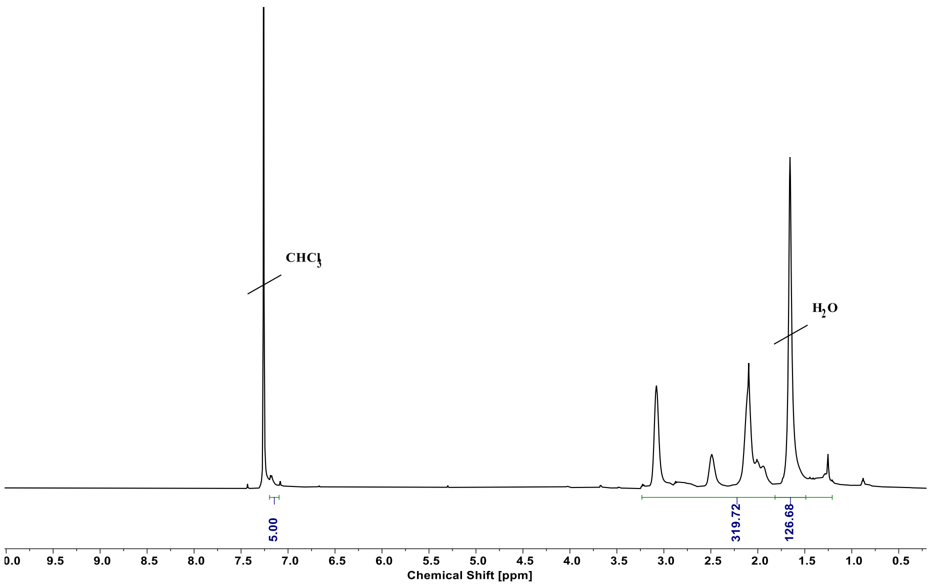

*Figure S12: ¹H-NMR spectra of p(PFPA) (**P2**) (600 MHz, $CDCl_3$).*

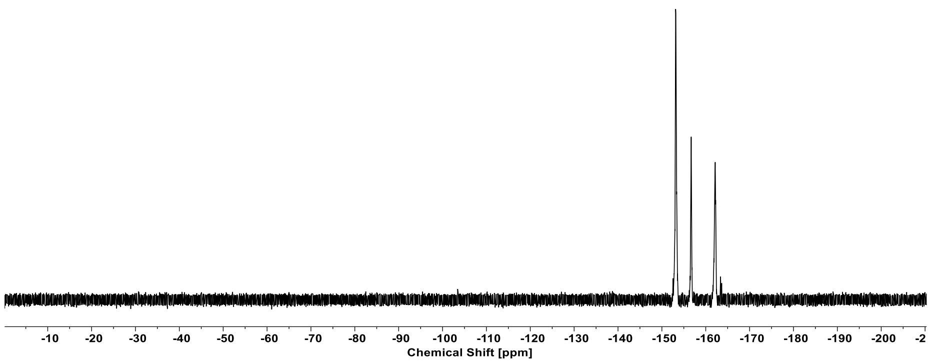

*Figure S13: ¹⁹F-NMR spectra of p(PFPA) (**P2**) (600 MHz, $CDCl_3$).*

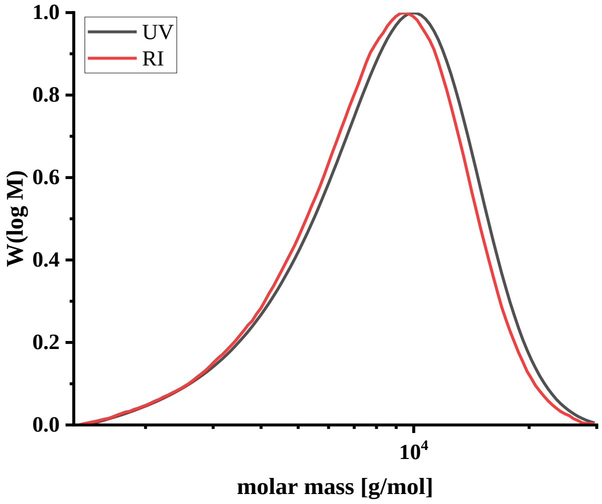

Figure S14: GPC measurement of p(PFPA) ***P2****, eluent: THF.*

P3 – p(PFPA)

^{1}H-NMR (600 MHz, Chloroform-*d*) δ (ppm) 7.21 – 7.13 (d_{br}, J = 6.9 Hz, 5H, *H1*), 3.31 – 0.78 (*H2 – H6*). Pn ~ 202

^{19}F-NMR (564 MHz, Chloroform-*d*) δ (ppm) -153.18 (s, *F1*), -156.70 (s, *F3*), -162.16 (s, *F2*).

GPC (THF, toluene standard) *UV detector:* M_n = 23488 g/mol, M_w = 32464 g/mol, Đ = 1.38, *RI detector:* M_n = 22542 g/mol, M_w = 30793 g/mol, Đ = 1.37.

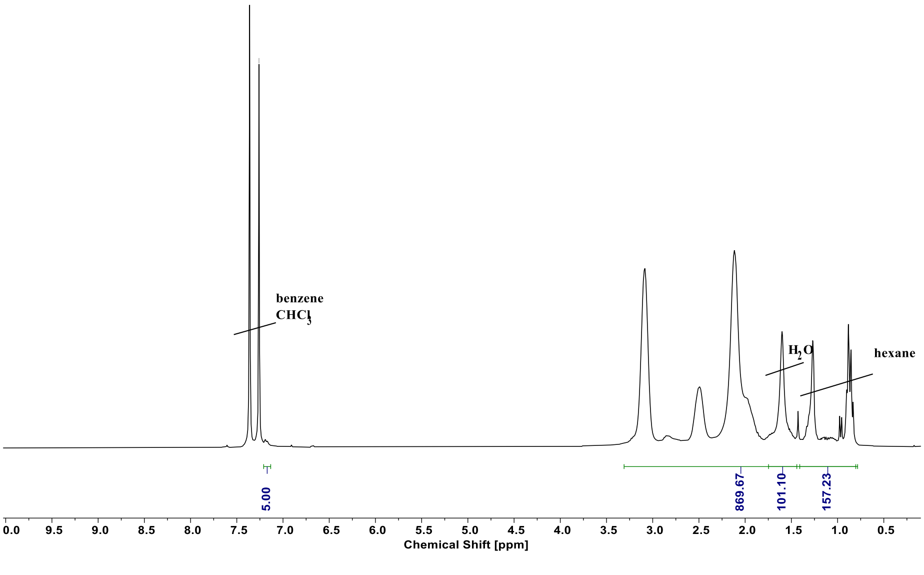

*Figure S15: ^{1}H-NMR spectra of p(PFPA) (**P3**) (600 MHz, $CDCl_3$).*

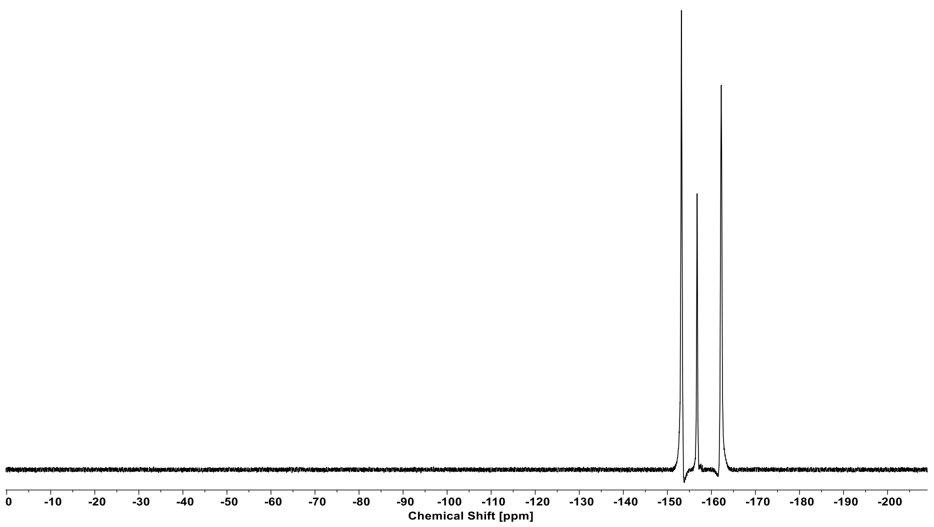

*Figure S16: ^{19}F-NMR spectra of p(PFPA) (**P3**) (600 MHz, $CDCl_3$).*

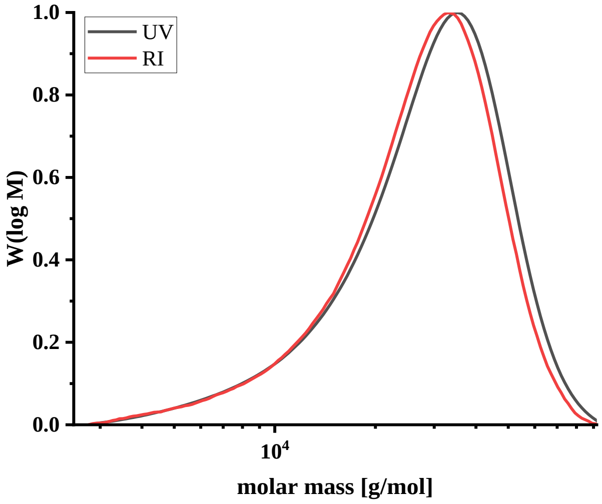

*Figure S17: GPC measurement of p(PFPA) **P3**, eluent: THF.*

P4-6 – p(HEAA)

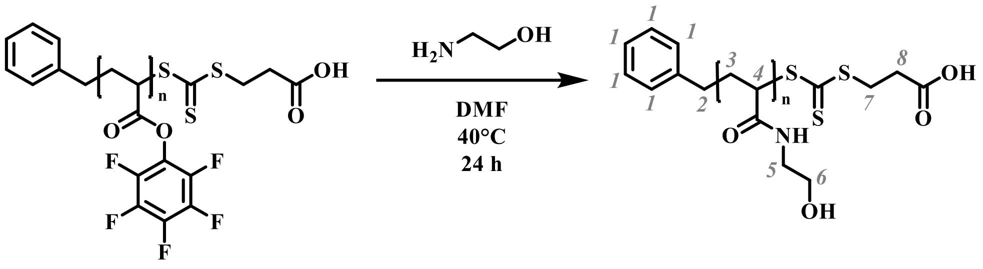

100 mg (0.42 mmol) of p(PFPA) (***P4-6***) was dissolved in 1 mL DMF, 291 µL (2.1 mmol, 5 eq. relative to the amine) triethylamine was added and mixed with 507 µL (8.4 mmol, 20 eq. relative to the repeating unit) ethanolamine and the reaction solution was stirred for 24 h at 40 °C in a sand bath. The product was then removed in cold acetone, centrifuged and purified by dialysis (cut off: 1 kDa) over three days and dried by lyophilization.

Yield: ***P4***: 31.18 mg, ***P5:*** 29.47 mg, **P6:** 36.67 mg

P4 – p(HEAA)

^{1}H-NMR (600 MHz, D_2O) δ (ppm) 7.32 – 6.99 (m, 5H, *H1*), 3.86 – 2.92 (m, *H5*, *H6*), 2.55 – 2.41 (m, 2H, *H7*), 2.26 – 1.16 (m, *H2*, *H3*, *H4*, *H8*). Pn ~ 29

^{19}F-NMR (564 MHz, D_2O) δ (ppm) No signals determined.

GPC (10 mM PBS, pH = 7.4, 298,15 K) *RI detector:* M_n = 4472 g/mol, M_w = 7256 g/mol, Đ = 1.48, *UV detector:* M_n = 4875 g/mol, M_w = 7196 g/mol, Đ = 1.62.

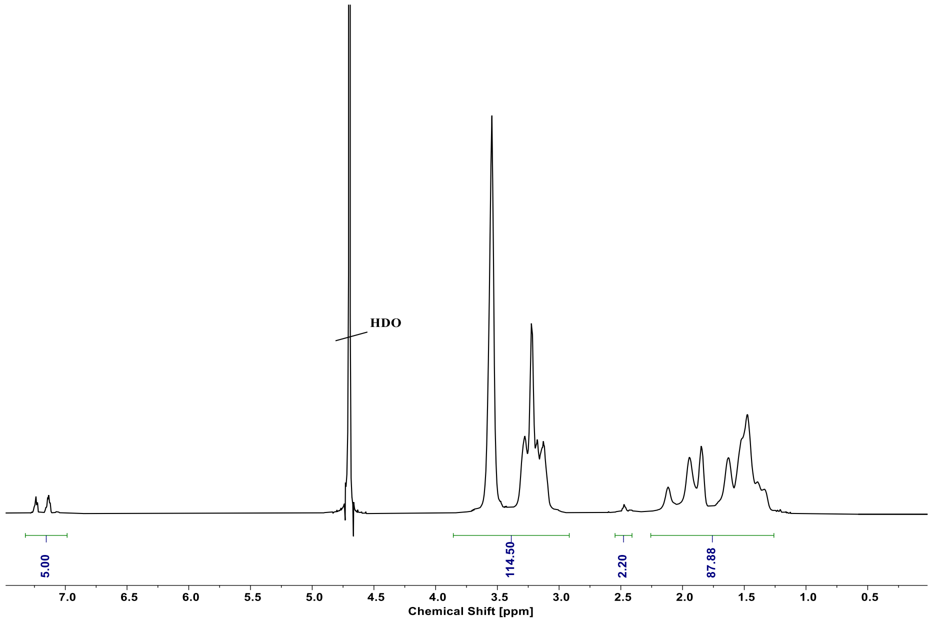

*Figure S18: ^{1}H-NMR spectra of p(HEAA) (**P4**) (600 MHz, D_2O).*

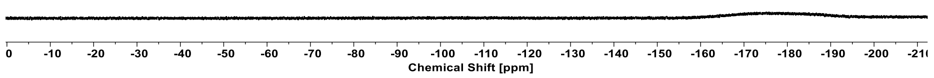

*Figure S19: ^{19}F-NMR spectra of p(HEAA) (**P4**) (600 MHz, D_2O).*

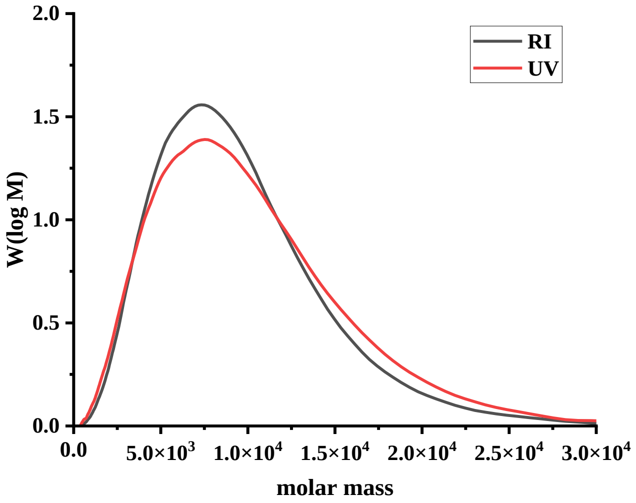

*Figure S20: Aqueous GPC measurement of p(HEAA) **P4**, eluent: PBS. RI Calculation.*

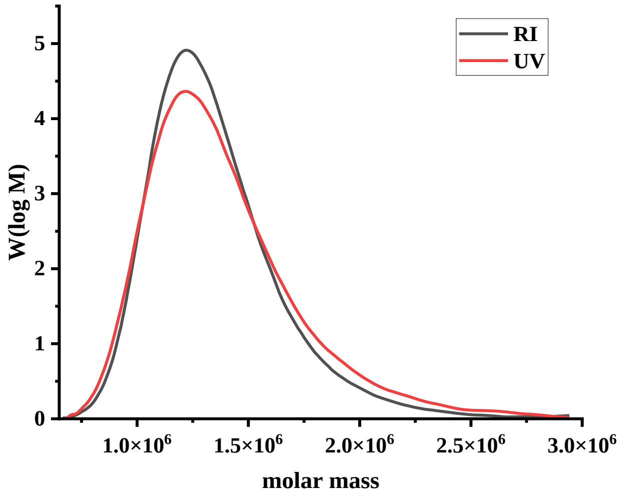

*Figure S21: Aqueous GPC measurement of p(HEAA) **P4**, eluent: PBS. LS Calculation.*

P5 – p(HEAA)

^{1}H-NMR (600 MHz, D_2O) δ (ppm) 7.35 – 6.99 (m, 5H, *H1*), 3.74 – 2.97 (m, *H5*, *H6*), 2.57 – 2.37 (m, 2H, *H7*), 2.21 – 1.22 (m, *H2*, *H3*, *H4*, *H8*). Pn ~ 57

^{19}F-NMR (564 MHz, D_2O) δ (ppm) No signals determined.

GPC (10 mM PBS, pH = 7.4, 298,15 K) *RI detector:* M_n = 7311.3 g/mol, M_w = 1003.2 g/mol, Đ = 1.37, *UV detector:* M_n = 6881 g/mol, M_w = 9984 g/mol, Đ = 1.45.

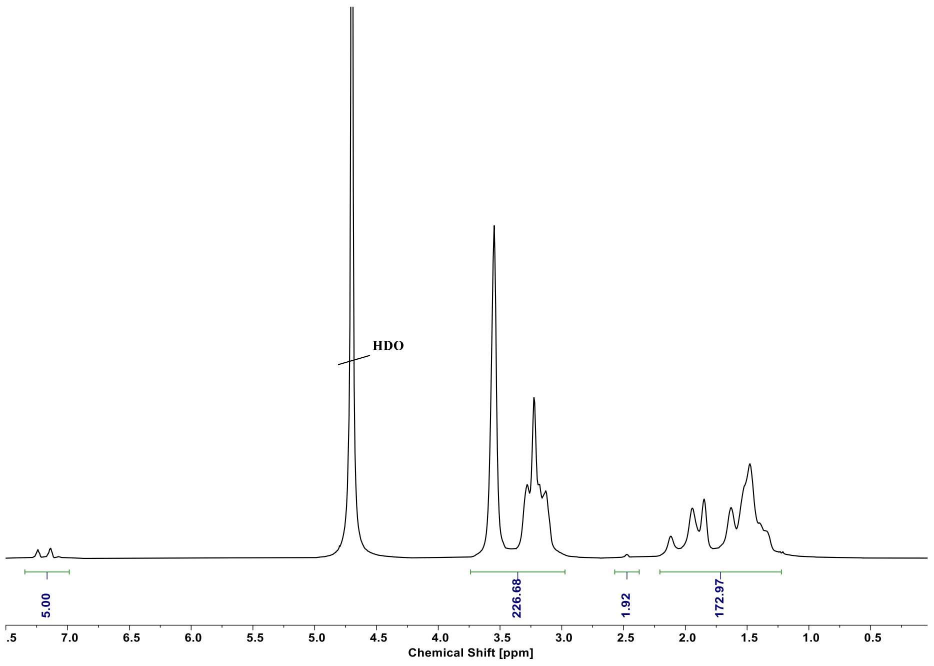

*Figure S22: ^{1}H-NMR spectra of p(HEAA) (**P5**) (600 MHz, D_2O).*

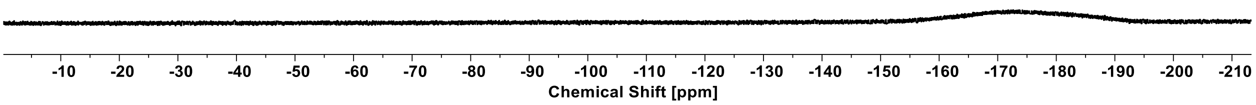

*Figure S23: ^{19}F-NMR spectra of p(HEAA) (**P5**) (600 MHz, D_2O).*

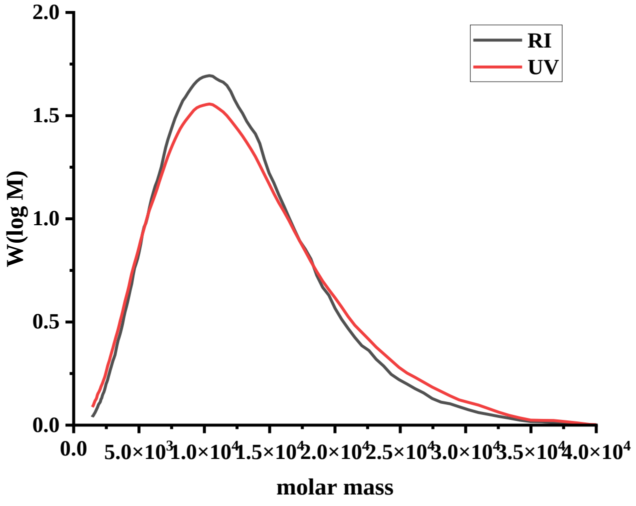

*Figure S24: Aqueous GPC measurement of p(HEAA) **P5**, eluent: PBS. RI Calculation.*

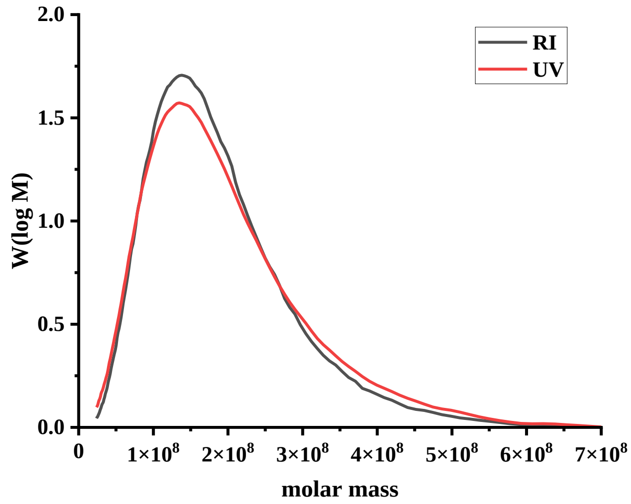

Figure S25: Aqueous GPC measurement of p(HEAA) ***P5****, eluent: PBS. LS Calculation.*

P6 – p(HEAA)

^{1}H-NMR (600 MHz, D_2O) δ (ppm) 7.27 – 7.05 (m, 5H, *H1*), 3.71 – 2.97 (m, *H5*, *H6*), 2.16 – 1.19 (m, *H2*, *H3*, *H4*, *H7*, *H8*). Pn ~ 235

^{19}F-NMR (564 MHz, D_2O) δ (ppm) No signals determined.

GPC (10 mM PBS, pH = 7.4, 298,15 K) *RI detector:* M_n = 21320 g/mol, M_w = 32360 g/mol, Đ = 1.52, *UV detector:* M_n = 16570 g/mol, M_w = 28120 g/mol, Đ = 1.70.

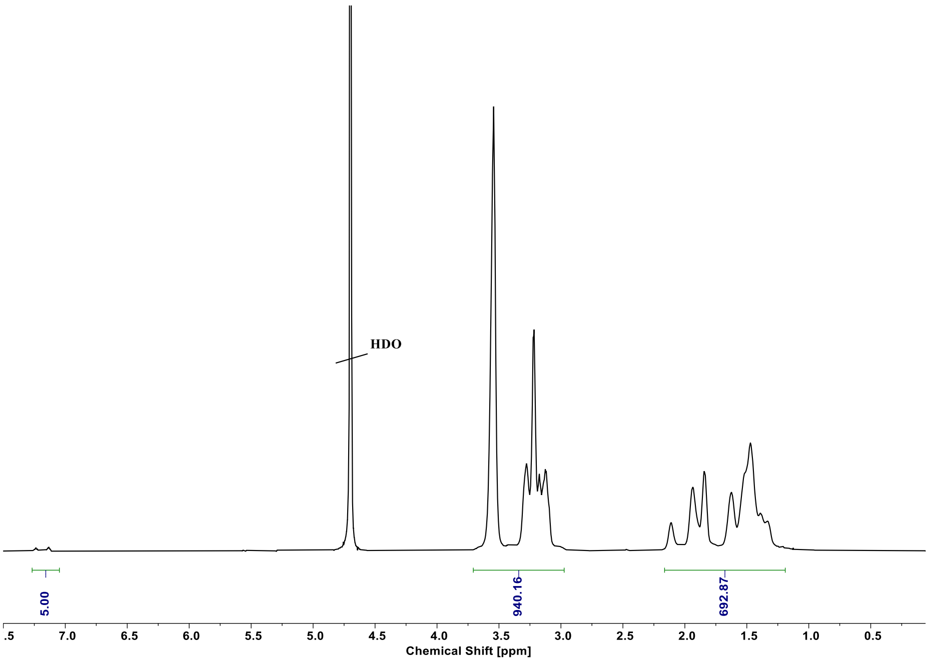

*Figure S26: ^{1}H-NMR spectra of p(HEAA) (**P6**) (600 MHz, D_2O).*

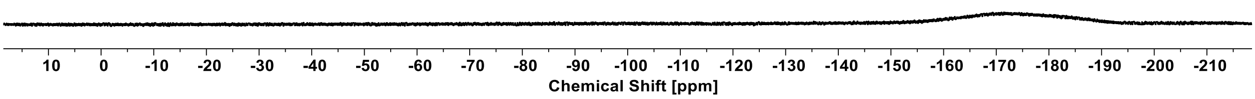

*Figure S27: ^{19}F-NMR spectra of p(HEAA) (**P6**) (600 MHz, D_2O).*

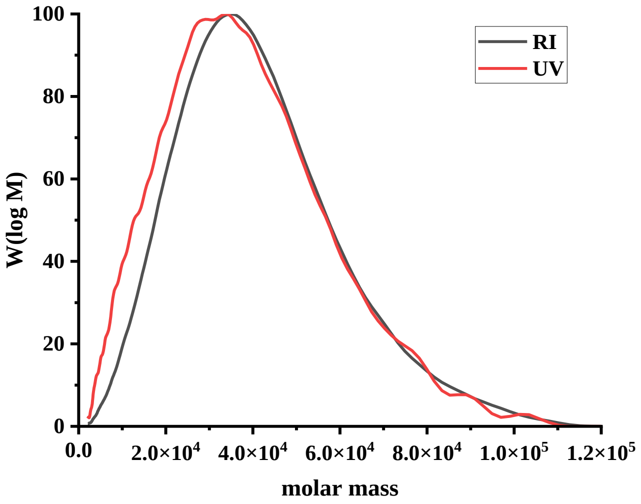

*Figure S28: Aqueous GPC measurement of p(HEAA) **P6**, eluent: PBS. RI Calculation.*

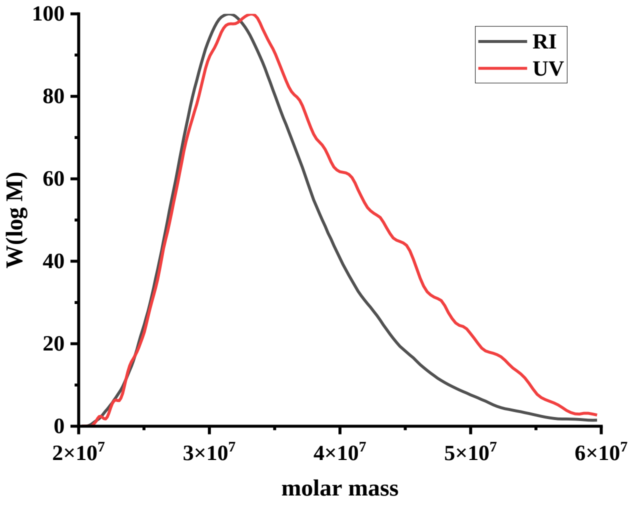

*Figure S29: Aqueous GPC measurement of p(HEAA) **P6**, eluent: PBS. LS Calculation.*

2.3 Oligoamidoamide synthesis of O1 and O2

Both mannose functionalized oligoamidoamides (O1, O2) were obtained by the application of solid phase synthesis, described in “1.3 General Methods”. The structures were synthesized in 0.2 mmol scale, were received as a white powder (***O1***: 249.56 mg, 1.9 mmol, 94% yield, ***O2***: 351.76 mg, 0.2 mmol, 92% yield) and were used without further purification.

O1 – monovalent N-functional oligoamidoamide

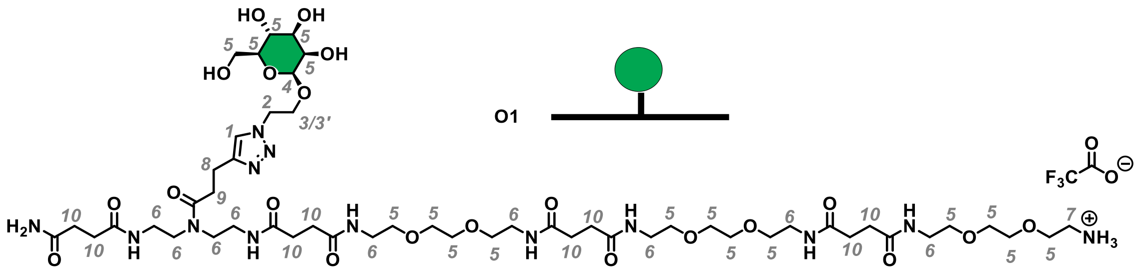

^{1}H NMR (300 MHz, D_2O) *δ (ppm)* 7.94 (s, 1H, *H1*), 4.68 – 4.64 (m, 2H, *H2*), 4.14 – 4.04 (m, 1H, *H3*), 3.97 – 3.89 (m, 1H, *H3'*), 3.88 – 3.84 (dd, ^3J = 3.3, 1.8 Hz, 1H, *H4*), 3.78 – 3.56 (m, 30H, *H5*), 3.50 – 3.32 (m, 18H, *H6*), 3.24 – 3.19 (t, ^3J = 5.1 Hz, 2H, *H7*), 3.07 – 3.01 (m, 2H, *H8*), 2.84 – 2.78 (m, 2H, *H9*), 2.56 – 2.46 (m, 16H, *H10*).

For a more definite analysis, the oligoamidoamine was analyzed once with protective groups via PR-HPLC-MS and once without protective groups.

LC-MS m/z calculated for $C_{74}H_{109}N_{13}O_{27}$ (with protecting groups) $[M + H]^+$ 1612.8, found 1613.6, $[M + H]^{2+}$ 806.9 found 807.0, $[M + H]^{3+}$ 538.3 found 538.3, determined relative purity: > 95%

LC-MS m/z calculated for $C_{66}H_{100}N_{13}O_{23}$ $[M + H]^{2+}$ 611.8 found 611.9, $[M + H]^{3+}$ 408.2 found 408.0

HR-ESI-MS m/z calculated for $C_{66}H_{100}N_{13}O_{23}$ $[M + H]^{3+}$ 611.8298 found 611.8322

MALDI m/z calculated for $C_{66}H_{100}N_{13}O_{23}$ $[M + H]^+$ 1222.66 found 1222.78, $[M + Na]^+$ 1244.64 found 1244.76, $[M + K]^+$ 1260.61 found 1260.74

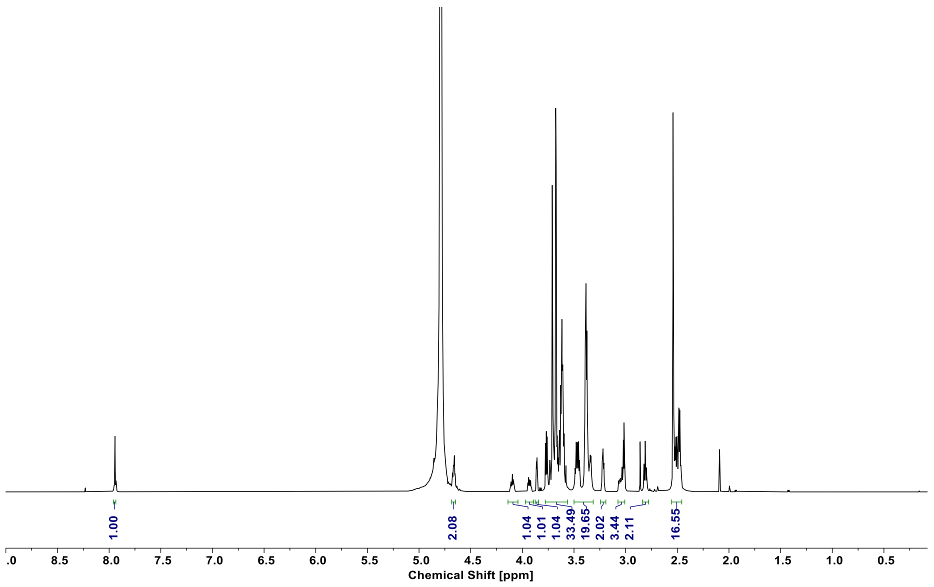

*Figure S30: ^{1}H-NMR spectra of monovalent oligoamidoamine (**O1**) (300 MHz, D_2O).*

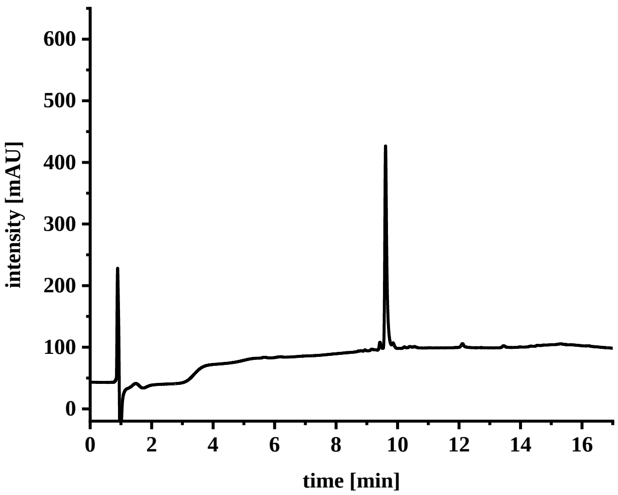

*Figure S31: RP-HPLC- chromatogram of monovalent oligoamidoamine (**O1**) with protecting groups (gradient of 5 to 95 vol% acetonitrile/water with 0.1 vol% formic acid, run time: 17 min).*

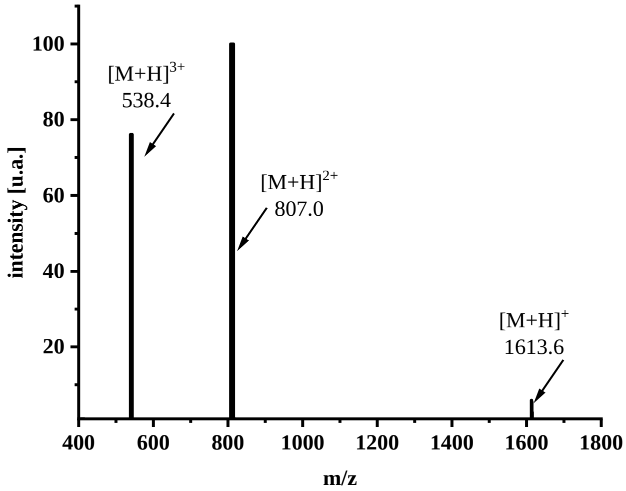

*Figure S32:ESI-MS spectra of monovalent oligoamidoamine (**O1**) with protecting groups at t = 9.61 min (gradient of 5 to 95 vol.% acetonitrile/water with 0.1 vol.% formic acid, run time: 17 min).*

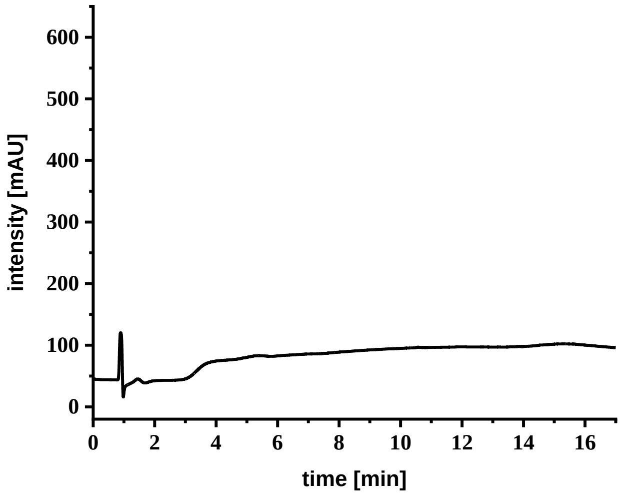

*Figure S33. RP-HPLC- chromatogram of monovalent oligoamidoamine (**O1**) (gradient of 5 to 95 vol% acetonitrile/water with 0.1 vol% formic acid, run time: 17 min). The product signal is detected in the injection peak.*

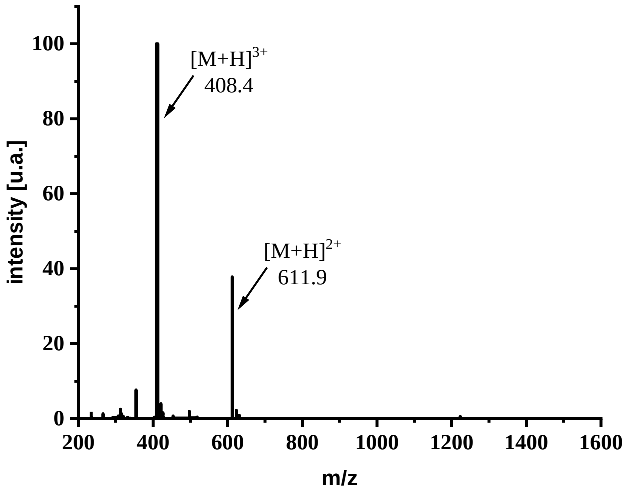

*Figure S34: ESI-MS spectra of monovalent oligoamidoamine (**O1**) at t = 0.89 min (gradient of 5 to 95 vol.% acetonitrile/water with 0.1 vol.% formic acid, run time: 17 min).*

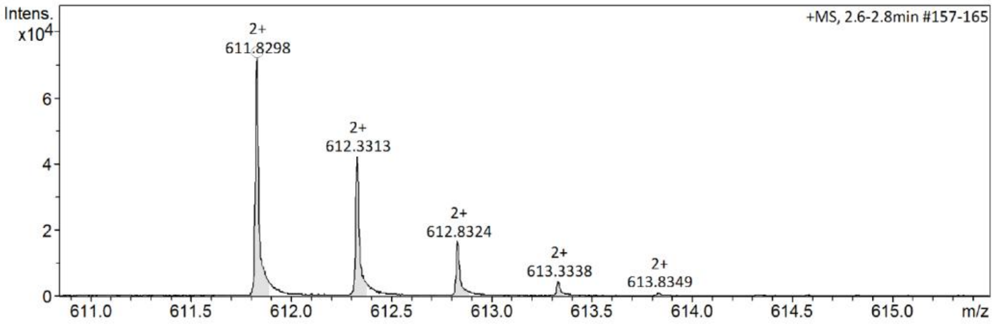

*Figure S35: HR-ESI-MS spectra of monovalent oligoamidoamine (**O1**).*

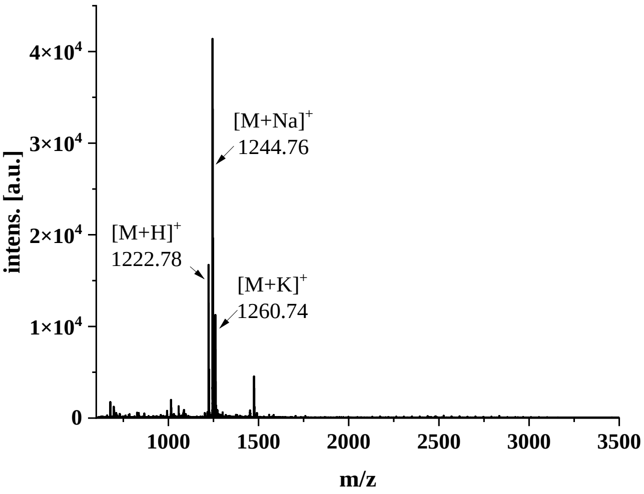

*Figure S36: MALDI spectra of chromatogram ofmonovalent oligoamidoamine (**O1**).*

O2 – trivalent N-functional oligoamidoamide

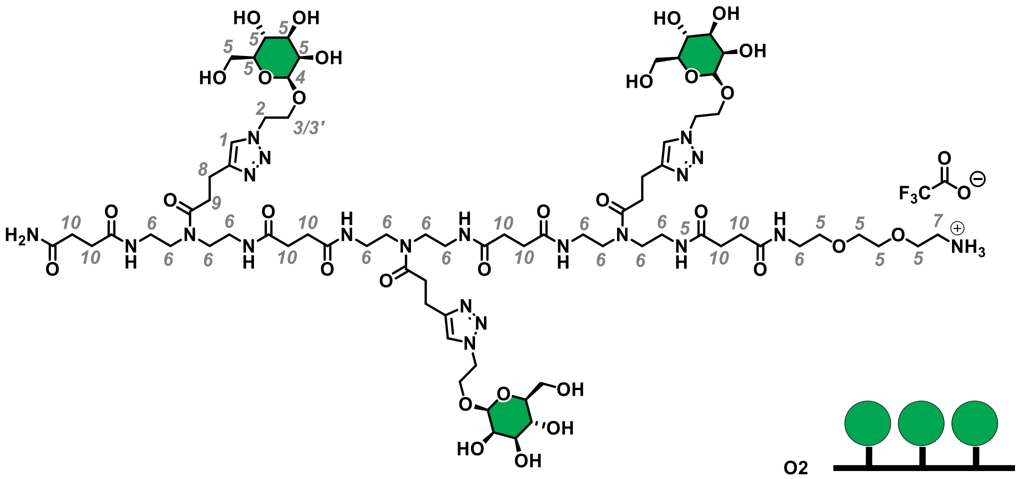

O2

^{1}H NMR (300 MHz, D_2O) *δ (ppm)* 7.96 (s, 3H, *H1*), 4.70 – 4.659 (m, 6H, *H2*), 4.15 – 4.05 (m, 3H, *H3*), 3.99 – 3.88 (m, 3H, *H3'*), 3.88 – 3.83 (m, 3H, *H4*), 3.80 – 3. 53 (m, 26H, *H5*), 3.53 – 3.26 (m, 26H, *H6*), 3.25 – 3.18 (t, ^3J = 5.3 – 2.98 (m, 6H overlap with DMF peak, *H8*), 2.88 – 2.74 (m, 6H overlap with DMF peak, *H9*), 2.57 – 2.38 (m, 16H, *H10*).

For a more definite analysis, the oligoamidoamine was analyzed once with protective groups via PR-HPLC-MS and once without protective groups.

LC-MS m/z calculated for $C_{112}H_{157}N_{21}O_{45}$ (with protecting groups) $[M + H]^{2+}$ 1259.0, found 1259.4, $[M + H]^{3+}$ 839.7 found 840.0, $[M + H]^{4+}$ 630.0 found 630.2, determined relative purity: > 91%

LC-MS m/z calculated for $C_{73}H_{123}N_{21}O_{31}$ $[M + H]^{2+}$ 896.4 found 896.5, $[M + H]^{3+}$ 598.0 found 597.8, $[M + H]^{4+}$ 448.7 found 448.6

HR-ESI-MS m/z calculated for $C_{73}H_{123}N_{21}O_{31}$ $[M + H]^{3+}$ 597.6306 found 597.6308

MALDI m/z calculated for $C_{73}H_{123}N_{21}O_{31}$ $[M + H]^+$ 1790.88 found 1791.06, $[M + Na]^+$ 1812.86 found 1813.03, $[M + K]^+$ 1828.83 found 1829.02

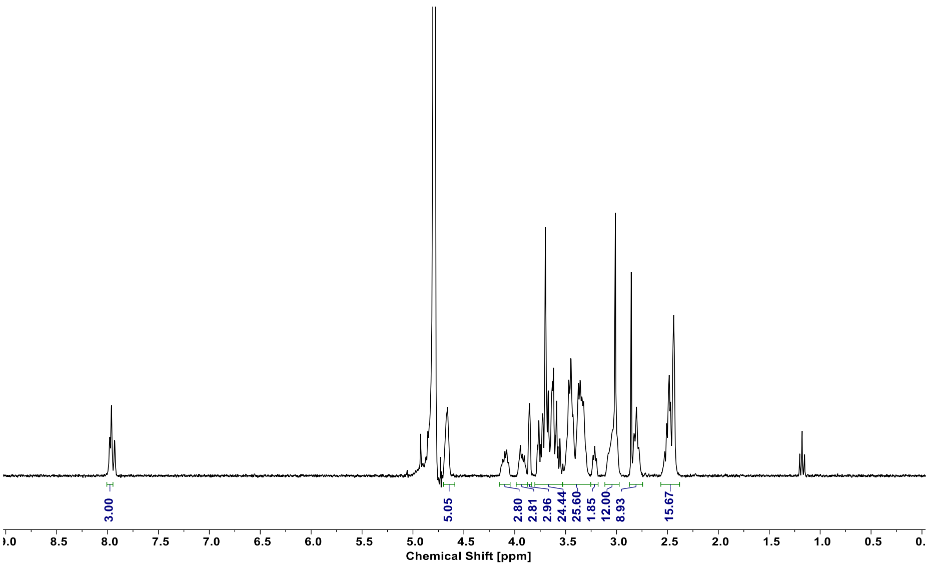

*Figure S37: ^{1}H-NMR spectra of trivalent oligoamidoamine (**O2**) (300 MHz, D_2O).*

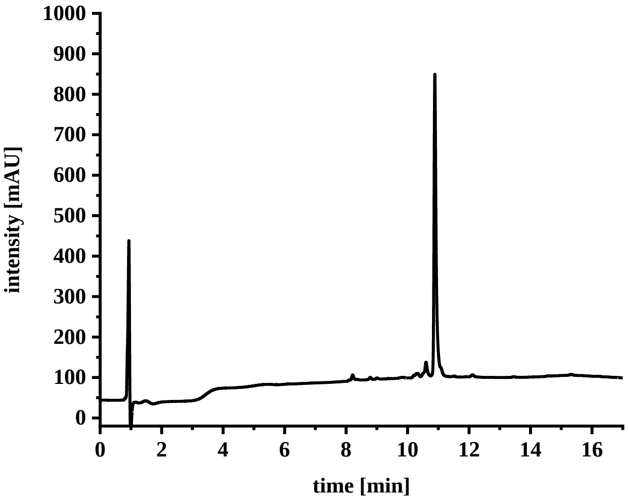

*Figure S38: RP-HPLC- chromatogram of trivalent oligoamidoamine (**O2**) with protecting groups (gradient of 5 to 95 vol% acetonitrile/water with 0.1 vol% formic acid, run time: 17 min).*

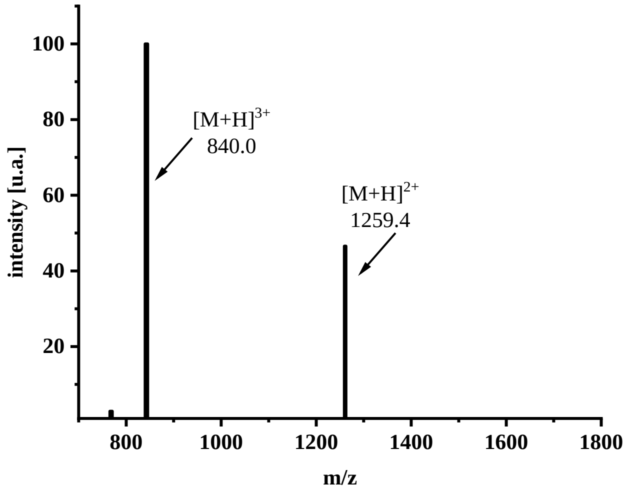

*Figure S39: ESI-MS spectra of trivalent oligoamidoamine (**O2**) with protecting groups at t = 10.88 min (gradient of 5 to 95 vol.% acetonitrile/water with 0.1 vol.% formic acid, run time: 17 min).*

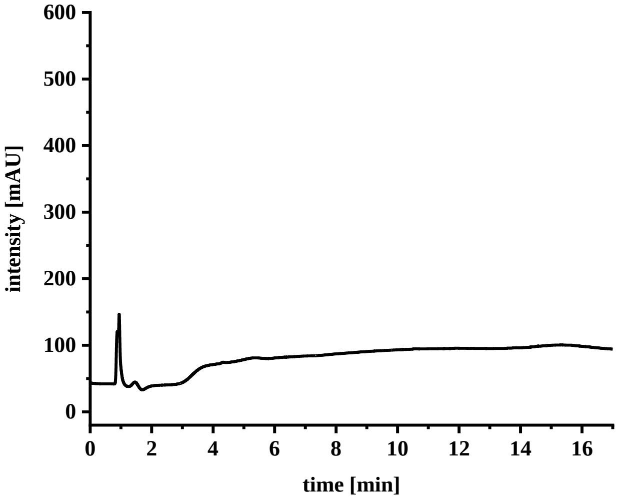

*Figure S40: RP-HPLC- chromatogram of trivalent oligoamidoamine (**O2**) (gradient of 5 to 95 vol% acetonitrile/water with 0.1 vol% formic acid, run time: 17 min). The product signal is detected in the injection peak.*

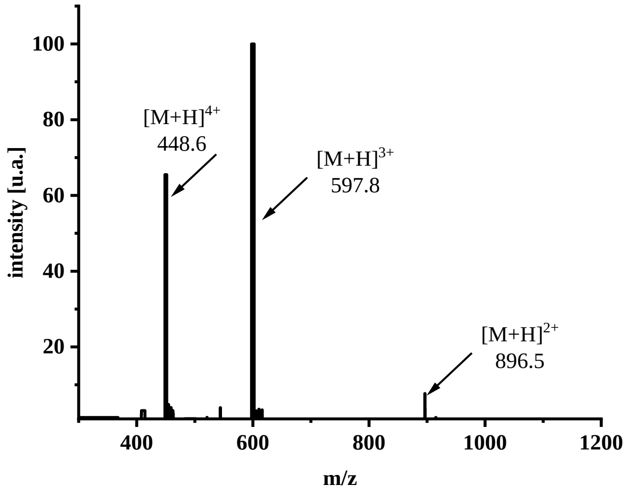

*Figure S41: ESI-MS spectra of trivalent oligoamidoamine (**O2**) at t = 0.94 min (gradient of 5 to 95 vol.% acetonitrile/water with 0.1 vol.% formic acid, run time: 17 min).*

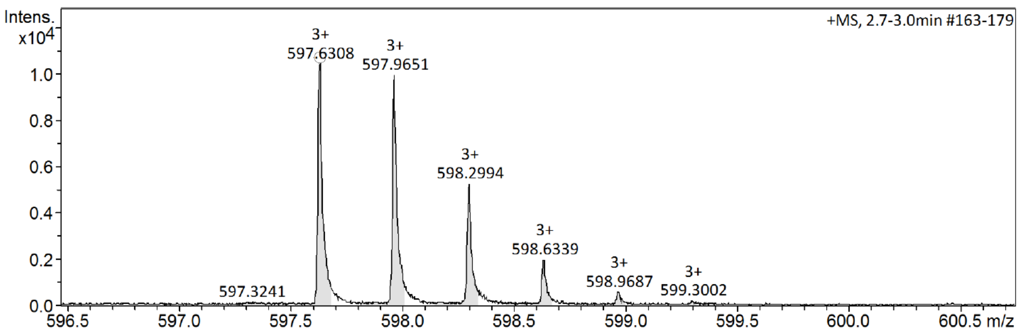

*Figure S42: HR-ESI-MS spectra of trivalent oligoamidoamine (**O2**).*

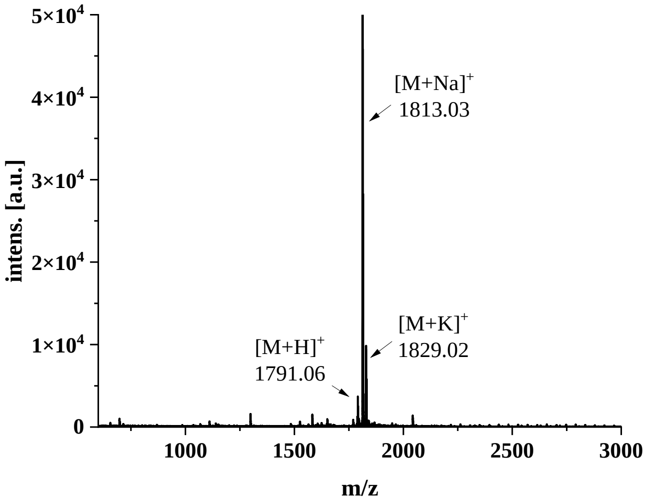

*Figure S43: MALDI spectra of trivalent oligoamidoamine (**O2**).*

2.4 Synthesis of side chain glycopolymers P7-P14

As all incorporation values brush and brush^2 structures are determined using ^{1}H-NMR spectroscopy in the following, the used molecular weights refer to the repeating units of polymers ***P4-6*** determined using ^{1}H-NMR.

P7 – ($_2E_{29}$)

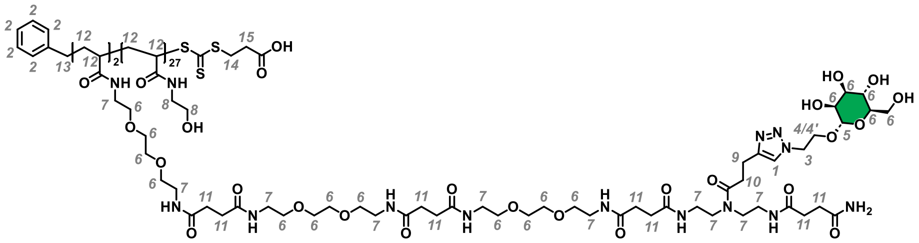

The synthesized polymer ***P7*** were obtained by the application of *synthesis of brush-like glycopolymers (arm-structures) and linear reference structures*, described in "1.3 General Methods". 50 mg (7 µmol, 210.1 µmol related to the repetition unit) of p(PFPA) ***P1*** were functionalized with oligoamidoamine ***O1*** (5%, 13.85 mg, 10.5 µmol) quenched with ethanolamine and were received after dialysis as a white powder (17.59 mg, 7% incorporation, 2.9 µmol, 42% yield).

^{1}H-NMR (600 MHz, D_2O) δ (ppm) 7.87 (s, 2H, *H1*), 7.35 – 7.05 (m, 5H, *H2*), 4.67 – 4.59 (m, *H3,* overlap with water peak), 4.12 – 4.04 (m, *H4*), 3.95 – 3.88 (m, *H4'*), 3.88 – 3.84 (m, *H5*), 3.83 – 3.09 (m, *H6* - *H8*), 3.04 – 0.86 (m, *H9- H 15*).

^{19}F-NMR (564 MHz, D_2O) δ (ppm) No signals determined.

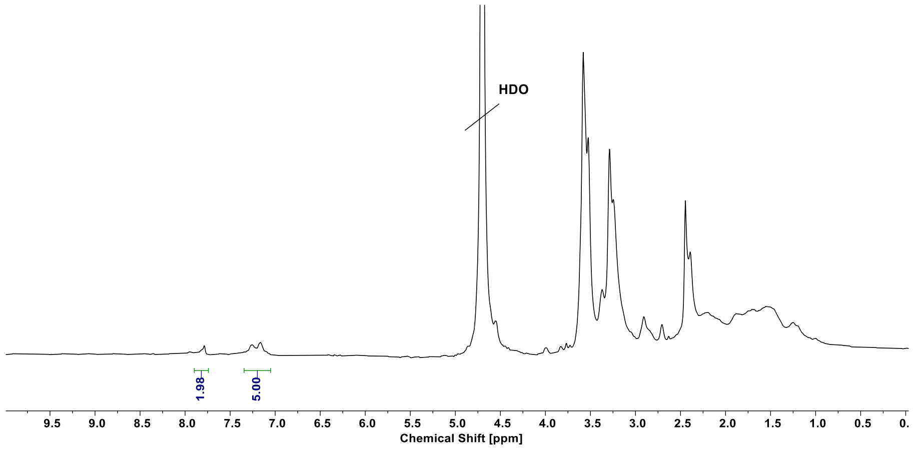

*Figure S44: ^{1}H-NMR spectra of $_2E_{29}$ (**P7**) (600 MHz, D_2O).*

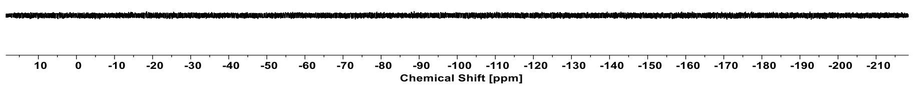

*Figure S45: ^{19}F-NMR spectra of $_2E_{29}$ (**P7**) (564 MHz, D_2O).*

P8 – ($_6E_{29}$)

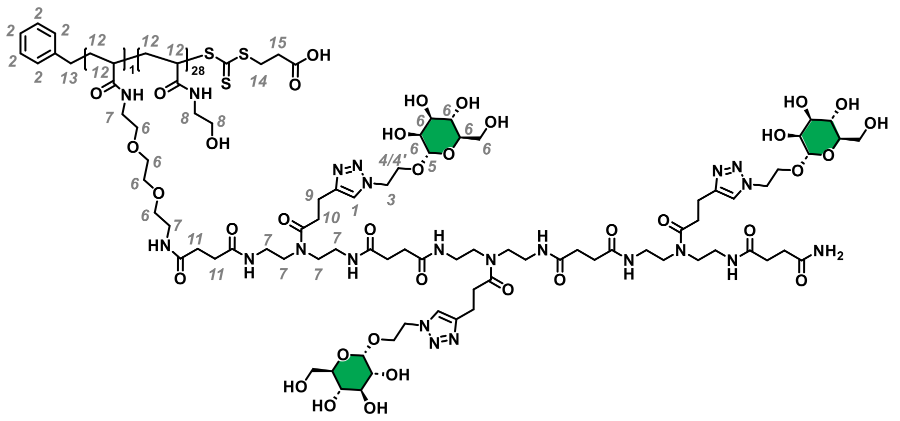

The synthesized polymer ***P8*** were obtained by the application of *synthesis of brush-like glycopolymers (arm-structures) and linear reference structures*, described in “1.3 General Methods”. 50 mg (7 µmol, 210.1 µmol related to the repetition unit) of p(PFPA) ***P1*** were functionalized with oligoamidoamine ***O2*** (5%, 19.84 mg, 10.5 µmol) quenched with ethanolamine and were received after dialysis as a white powder (17.8 mg, 3% incorporation, 5.22 µmol, 75% yield).

^{1}H-NMR (300 MHz, D_2O) δ (ppm) 7.87 (s, 3H, *H1*), 7.47 – 7.16 (m, 5H, *H2*), 4.72 – 4.54 (m, *H3*), 4.14 – 4.02 (m, *H4*), 3.97 – 3.83 (m, *H4’, H5*), 3.83 – 3.53 (m, *H6*, *H8*), 3.53 – 3.14 (m, *H7*, *H8*), 3.06 – 2.87 (m, *H9*), 2.87 – 2.72 (m, *H10*), 2.58 – 2.38 (m, *H11*), 3.08 – 0.99 (m, *H12 – H15*).

^{19}F-NMR (282 MHz, D_2O) δ (ppm) No signals determined.

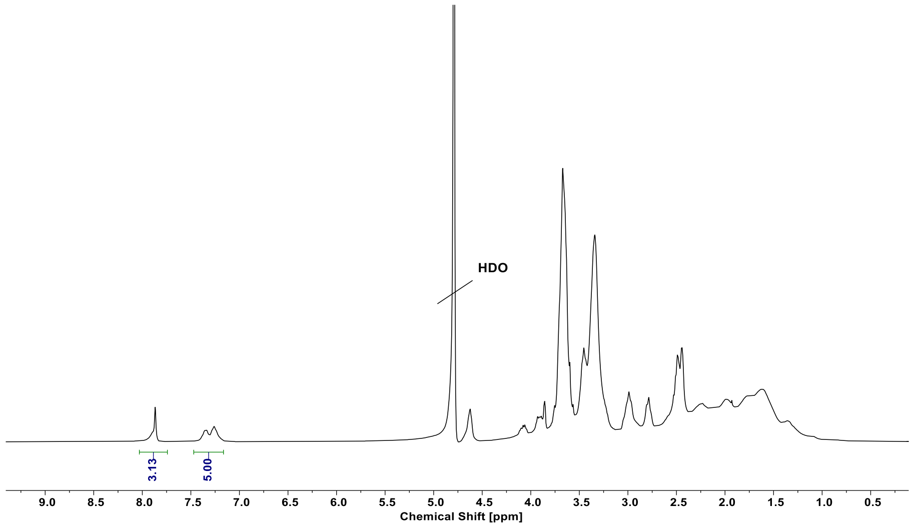

*Figure S46: ^{1}H-NMR spectra of $_6E_{29}$ (**P8**) (300 MHz, D_2O).*

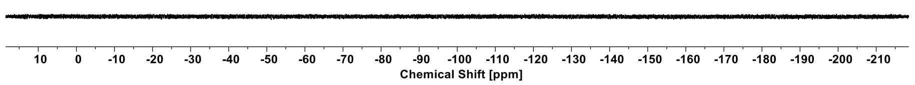

*Figure S47: ^{19}F-NMR spectra of $_6E_{29}$ (**P8**) (282 MHz, D_2O).*

***P9* – (${}_{2}\boldsymbol{I}_{29}$)**

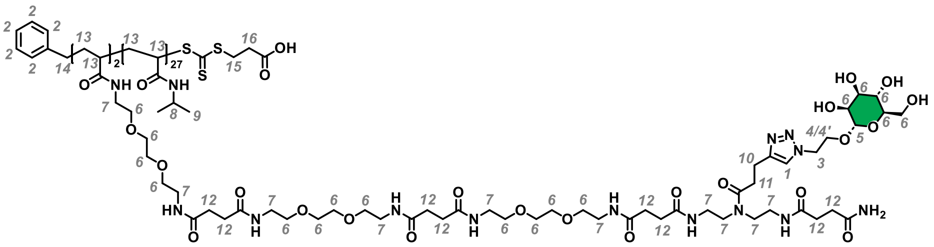

The synthesized polymer ***P9*** were obtained by the application of *synthesis of brush-like glycopolymers (arm-structures) and linear reference structures*, described in "1.3 General Methods". 50 mg (7 µmol, 210.1 µmol related to the repetition unit) of p(PFPA) ***P1*** were functionalized with oligoamidoamine ***O1*** (5%, 13.85 mg, 10.5 µmol) quenched with isopropylamine and were received after dialysis as a white powder (20.10 mg, 7% incorporation, 3.43 µmol, 49% yield).

^{1}H-NMR (600 MHz, D_2O) δ (ppm) 7.87 (s, 2H, *H1*), 7.44 – 7.06 (m, 5H, *H2*), 4.67 – 4.57 (m, *H3,* overlap with water peak), 4.22 – 3.79 (m, *H4, H4', H5, H8*), 3.75 – 3.53 (m, *H6*) 3.53 – 3.20 (m, *H7*), 3.08 – 2.96 (m, *H10*), 2.86 – 2.74 (m, *H11)*, 2.70 – 2.40 (m, *H12*), 1.26 – 0.96 (m, *H9*), 3.14 – 0.48 (m, *H13- H16*).

^{19}F-NMR (282 MHz, D_2O) δ (ppm) No signals determined.

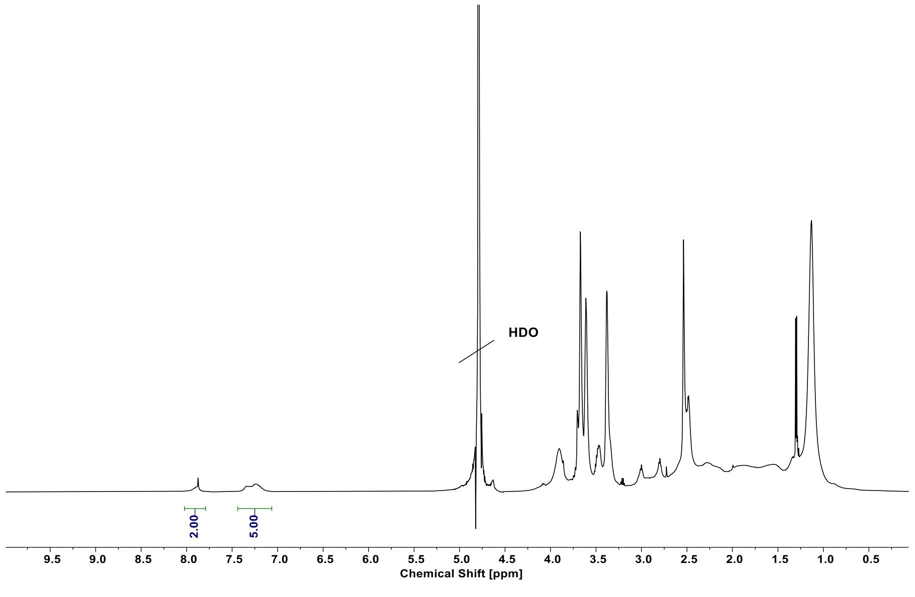

*Figure S48: ^{1}H-NMR spectra of ${}_{2}\boldsymbol{I}_{29}$ (**P9**) (600 MHz, D_2O).*

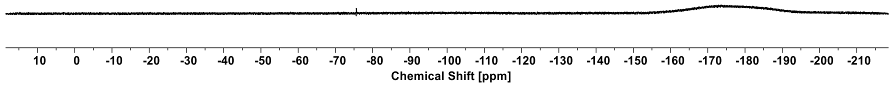

*Figure S49: ^{19}F-NMR spectra of ${}_{2}\boldsymbol{I}_{29}$ (**P9**) (564 MHz, D_2O).*

***P10* – ($_6\boldsymbol{I}_{29}$)**

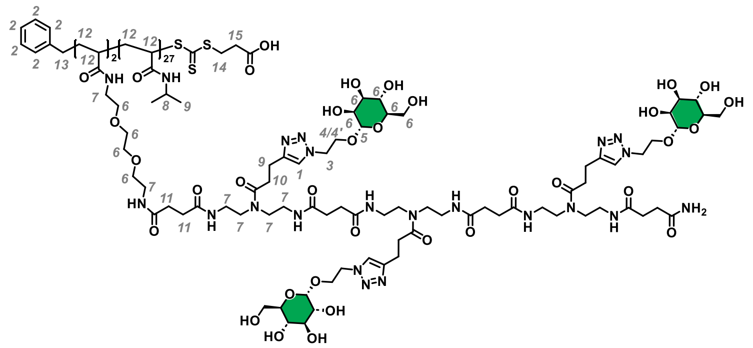

The synthesized polymer ***P10*** were obtained by the application of *synthesis of brush-like glycopolymers (arm-structures) and linear reference structures*, described in “1.3 General Methods”. 50 mg (7 µmol, 210.1 µmol related to the repetition unit) of p(PFPA) ***P1*** were functionalized with oligoamidoamine ***O2*** (5%, 19.84 mg, 10.5 µmol) quenched with isopropylamine and were received after dialysis as a white powder (28.06 mg, 7% incorporation, 4.0 µmol, 57% yield).

^{1}H-NMR (300 MHz, D_2O) δ (ppm) 7.87 (s, 5H, *H1*), 7.39 – 7.17 (m, 5H, *H2*), 4.70 – 4.55 (m, *H3*), 4.16 – 3.79 (m, *H4, H4’, H5, H8*), 3.80 – 3.54 (m, *H6*), 3.54 – 3.17 (m, *H7*), 3.08 – 2.89 (m, *H9*), 2.89 – 2.71 (m, *H10*), 2.57 – 2.36 (m, *H11*), 1.25 – 0.93 (m, *H9*), 3.15 – 0.46 (m, *H12 – H15*).

^{19}F-NMR (282 MHz, D_2O) δ (ppm) No signals determined.

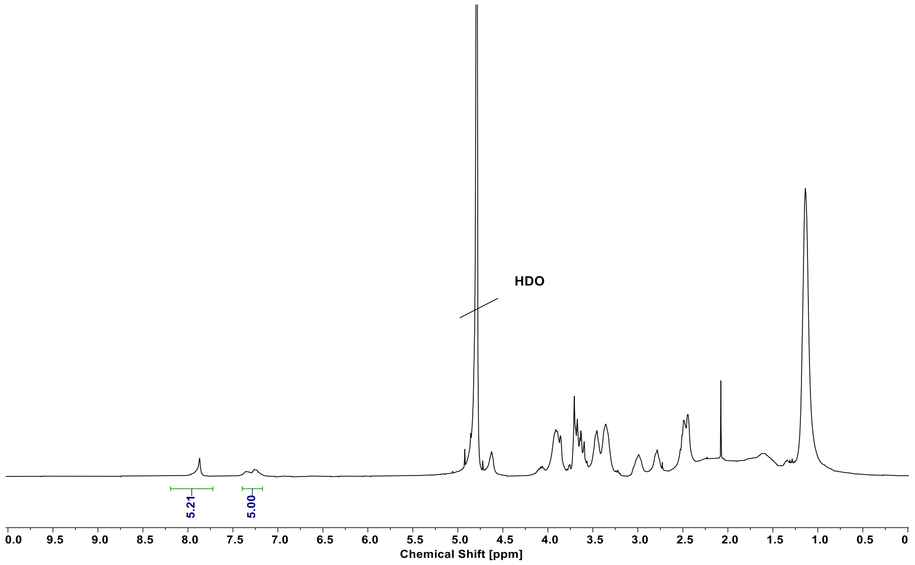

*Figure S50: ^{1}H-NMR spectra of $_6I_{29}$ (**P10**) (300 MHz, D_2O).*

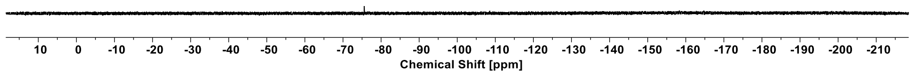

*Figure S51: ^{19}F-NMR spectra of $_6I_{29}$ (**P10**) (282 MHz, D_2O).*

***P11* – ($_{6}E_{57}$)**

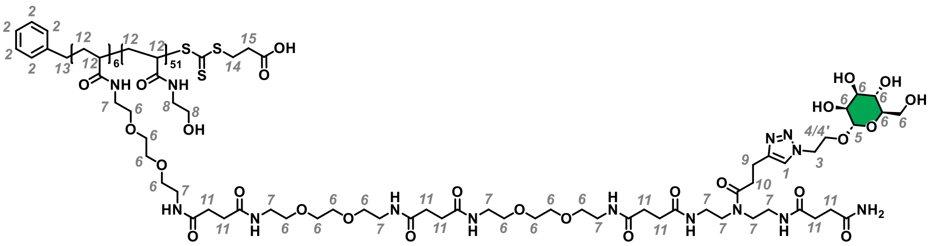

The synthesized polymer ***P11*** were obtained by the application of *synthesis of brush-like glycopolymers (arm-structures) and linear reference structures*, described in “1.3 General Methods”. 50 mg (3.6 µmol, 210.1 µmol related to the repetition unit) of p(PFPA) ***P2*** were functionalized with oligoamidoamine ***O1*** (15%, 41.55 mg, 31.51 µmol) quenched with ethanolamine and were received after dialysis as a white powder (38.18 mg, 11% incorporation, 2.77 µmol, 77% yield).

^{1}H-NMR (600 MHz, D_2O) δ (ppm) 7.88 (s, 6H, *H1*), 7.38 – 7.10 (m, 5H, *H2*), 4.66 – 4.61 (m, *H3,* overlap with water peak), 4.11 – 4.06 (m, *H4*), 3.94 – 3.89 (m, *H4’*), 3.87 – 3.85 (m, *H5*), 3.79 – 3.17 (m, *H6* - *H8*), 3.06 – 1.03 (m, *H9 – H15*).

^{19}F-NMR (282 MHz, D_2O) δ (ppm) No signals determined.

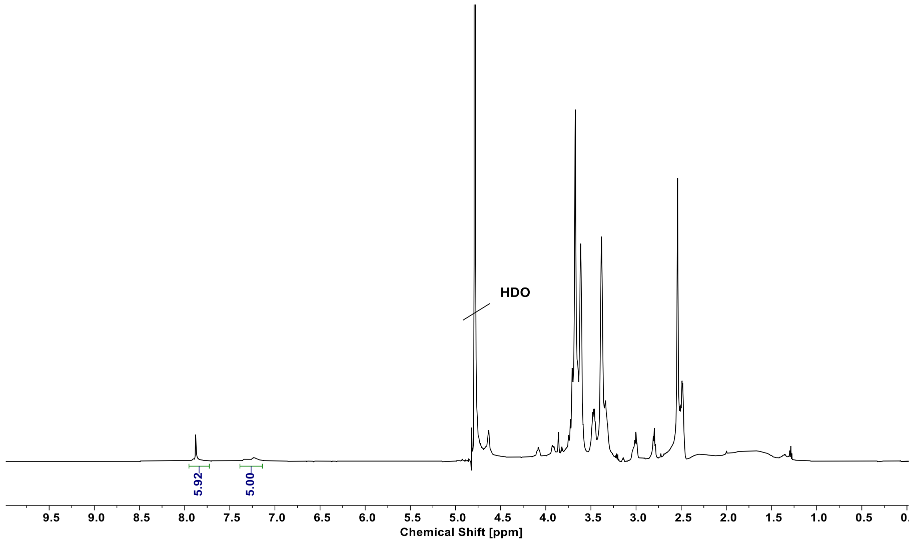

*Figure S52: ^{1}H-NMR spectra of $_{6}E_{57}$ (**P11**) (600 MHz, D_2O).*

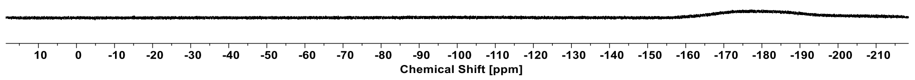

*Figure S53: ^{19}F-NMR spectra of $_{6}E_{57}$ (**P11**) (564 MHz, D_2O).*

***P12* – ($_{18}E_{57}$)**

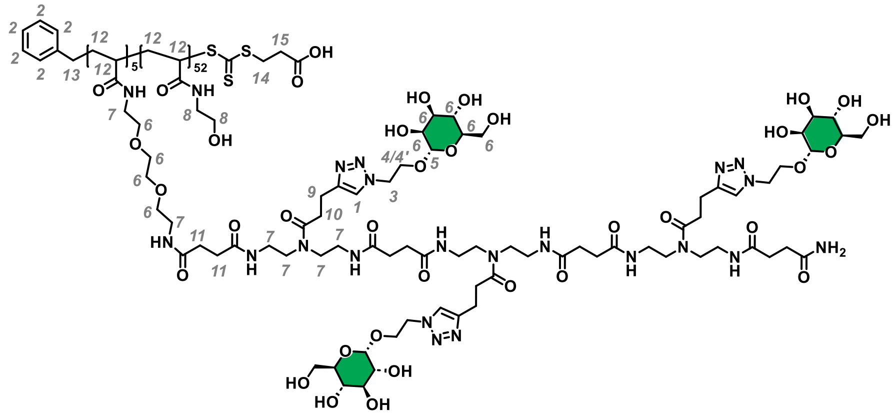

The synthesized polymer ***P12*** were obtained by the application of *synthesis of brush-like glycopolymers (arm-structures) and linear reference structures*, described in "1.3 General Methods". 50 mg (3.6 µmol, 210.1 µmol related to the repetition unit) of p(PFPA) ***P2*** were functionalized with oligoamidoamine ***O2*** (15%, 59.52 mg, 31.51 µmol) quenched with ethanolamine and were received after dialysis as a white powder (40.49 mg, 9% incorporation, 2.62 µmol, 73% yield).

^{1}H-NMR (300 MHz, D_2O) δ (ppm) 7.87 (s, 3H, *H1*), 7.47 – 7.10 (m, 5H, *H2*), 4.72 – 4.55 (m, *H3*), 4.13 – 4.02 (m, *H4*), 3.96 – 3.82 (m, *H4', H5*), 3.80 – 3.54 (m, *H6*, *H8*), 3.54 – 3.14 (m, *H7*, *H8*), 3.08 – 2.88 (m, *H9*), 2.88 – 2.71 (m, *H10*), 2.57 – 2.35 (m, *H11*), 3.12 – 0.57 (m, *H12 – H15*).

^{19}F-NMR (282 MHz, D_2O) δ (ppm) No signals determined.

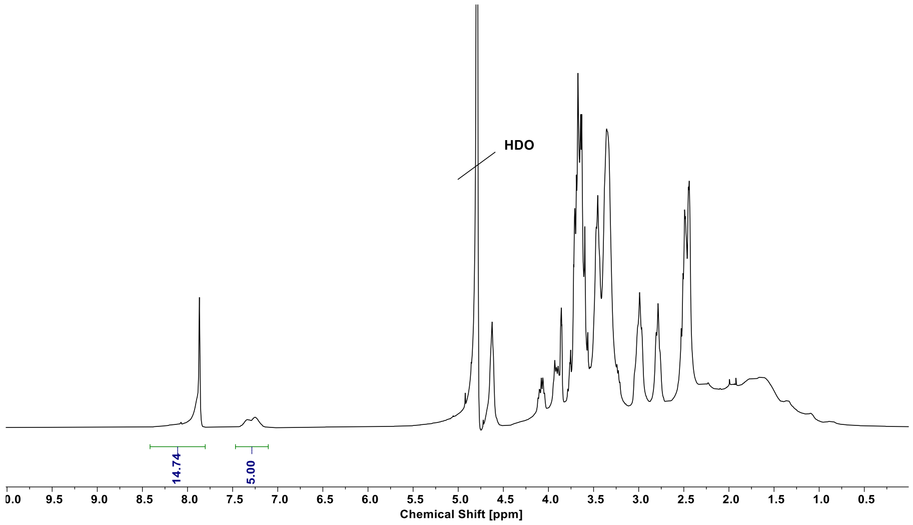

*Figure S54: ^{1}H-NMR spectra of $_{18}E_{57}$ (**P12**) (300 MHz, D_2O).*

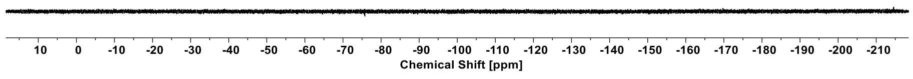

*Figure S55: ^{19}F-NMR spectra of $_{18}E_{57}$ (**P12**) (282 MHz, D_2O).*

***P13* – ($_6I_{57}$)**

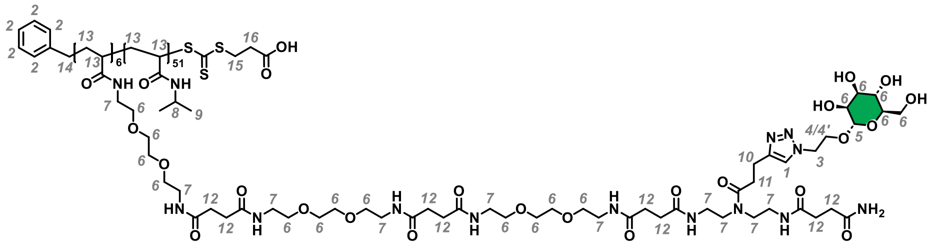

The synthesized polymer ***P13*** were obtained by the application of *synthesis of brush-like glycopolymers (arm-structures) and linear reference structures*, described in "1.3 General Methods". 50 mg (3.6 µmol, 210.1 µmol related to the repetition unit) of p(PFPA) ***P2*** were functionalized with oligoamidoamine ***O1*** (15%, 41.55 mg, 31.51 µmol) quenched with isopropylamine and were received after dialysis as a white powder (39.57 mg, 11% incorporation, 2.89 µmol, 80% yield).

^{1}H-NMR (300 MHz, D_2O) δ (ppm) 7.87 (s, 6H, *H1*), 7.41 – 7.11 (m, 5H, *H2*), 4.69– 4.57 (m, *H3,* overlap with water peak), 4.11 – 4.05 (m, *H4*), 3.99 – 3.84 (m, *H4', H5, H8*), 3.75 – 3.52 (m, *H6*) 3.52 – 3.28 (m, *H7, H8*), 3.05 – 2.96 (m, *H10*), 2.85 – 2.74 (m, *H11)*, 2.67 – 2.41 (m, *H12*), 1.24– 0.95 (m, *H9*), 3.14 – 0.74 (m, *H13- H16*).

^{19}F-NMR (282 MHz, D_2O) δ (ppm) No signals determined.

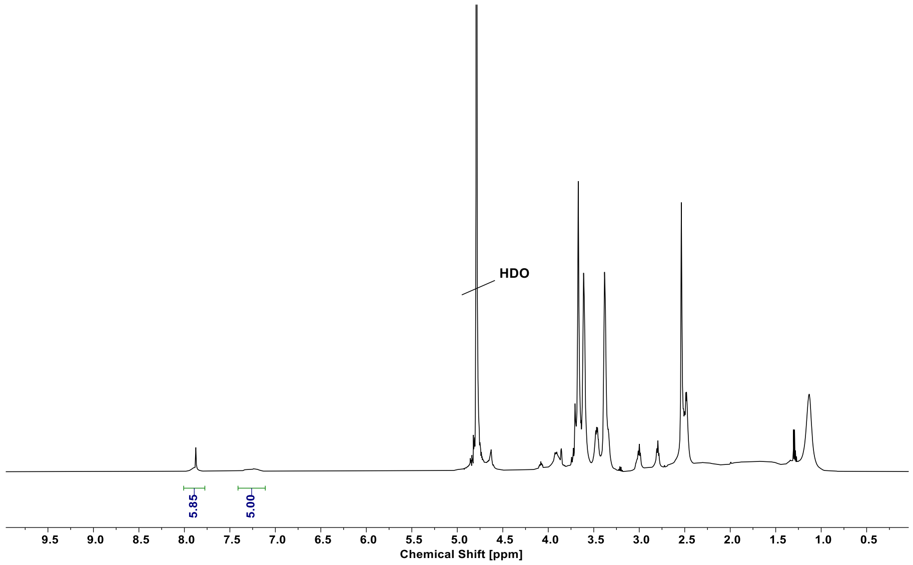

*Figure S56: ^{1}H-NMR spectra of $_6I_{57}$ (**P13**) (600 MHz, D_2O).*

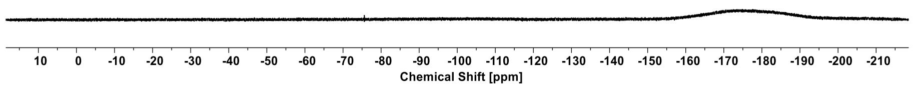

*Figure S57: ^{19}F-NMR spectra of $_6I_{57}$ (**P13**) (564 MHz, D_2O).*

***P14* – (${}_{18}I_{57}$)**

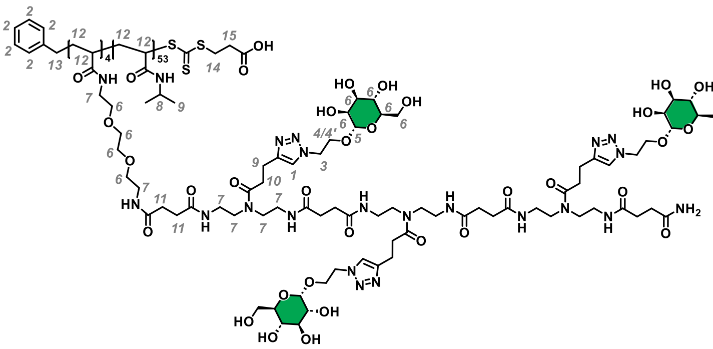

The synthesized polymer ***P14*** were obtained by the application of *synthesis of brush-like glycopolymers (arm-structures) and linear reference structures*, described in "1.3 General Methods". 50 mg (3.6 µmol, 210.1 µmol related to the repetition unit) of p(PFPA) ***P2*** were functionalized with oligoamidoamine ***O2*** (15%, 59.52 mg, 31.51 µmol) quenched with isopropylamine and were received after dialysis as a white powder (34.5 mg, 7% incorporation, 2.53 µmol, 70% yield).

^{1}H-NMR (300 MHz, D_2O) δ (ppm) 7.87 (s, 12H, *H1*), 7.43 – 7.05 (m, 5H, *H2*), 4.71 – 4.54 (m, *H3*), 4.12 – 4.01 (m, *H4*), 4.01 – 3.80 (m, *H4', H5, H8*), 3.80 – 3.55 (m, *H6*), 3.55 – 3.16 (m, *H7*), 3.07 – 2.89 (m, *H9*), 2.89 – 2.72 (m, *H10*), 2.58 – 2.34 (m, *H11*), 1.27 – 0.95 (m, *H9*), 3.14 – 0.56 (m, *H12 – H15*).

^{19}F-NMR (282 MHz, D_2O) δ (ppm) No signals determined.

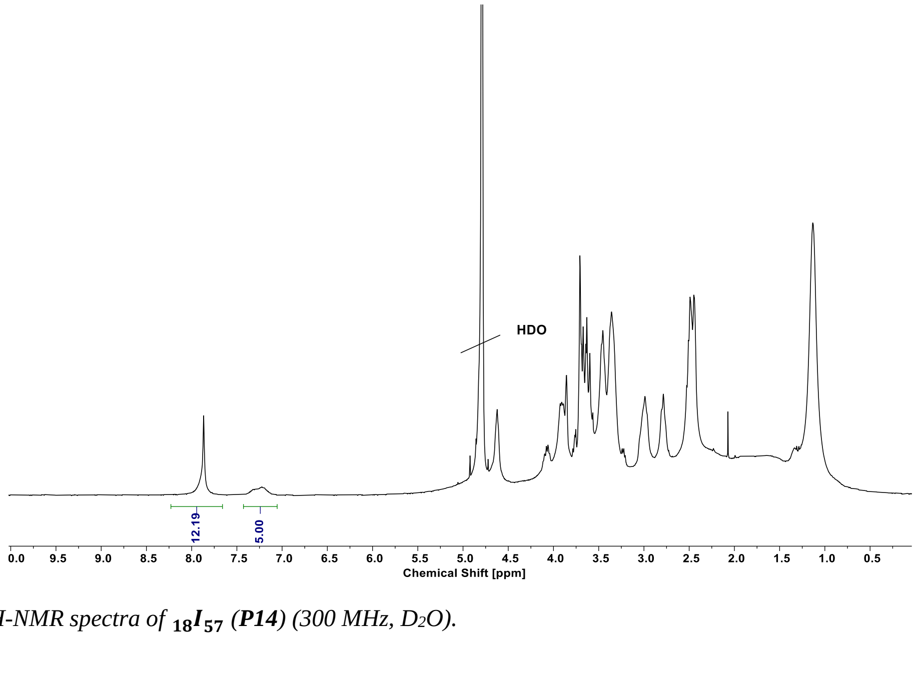

*Figure S58: ^{1}H-NMR spectra of ${}_{18}I_{57}$ (**P14**) (300 MHz, D_2O).*

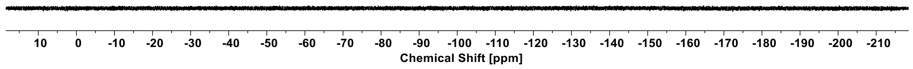

*Figure S59: ^{19}F-NMR spectra of ${}_{18}I_{57}$ (**P14**) (282 MHz, D_2O).*

2.5 Synthesis of brush²-glycopolymers B1-B16

B1 $-\ ({}_{22}E^{2}_{29})$

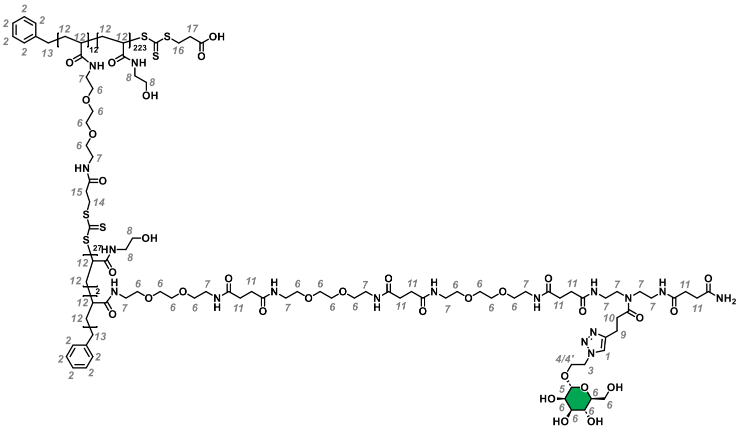

The synthesized brush² glycopolymer ***B1*** were obtained by the application of two steps: (1) *Attachment of the bridge molecule to arm polymers,* followed by *Cleavage of the protecting group of the conjugated bridge molecule* (2) *Attachment of the amine-activated arm polymers to the long polymer to gain brush² structures* described in "1.3 General Methods". 13 mg (0.23 µmol, 54.62 µmol related to the repetition unit) of p(PFPA) ***P3*** were functionalized with amine-preactivated arm polymer with comes from ***P7*** (5%, 16.79 mg, 2.73 µmol) quenched with ethanolamine and were received after dialysis as a white powder (13.77 mg, 5% incorporation, 0.14 µmol, 61% yield).

^{1}H-NMR (600 MHz, D_2O) δ (ppm) 7.88 (s, 24H, *H1*), 7.43 – 6.94 (m, 65H, *H2*), 4.66 – 4.59 (m, *H3,* overlap with water peak), 4.12 – 4.05 (m, *H4*), 3.96 – 3.12 (m, *H4', H5* - *H8*), 3.05 – 2.97 (m, *H9*), 2.97 – 2.87 (m, *H15*), 2.87 – 2.76 (m, *H10*), 2.64 – 2.42 (m, *H11*), 3.11 – 0.79 (m, *H12- H 14, H16, H17*).

^{19}F-NMR (282 MHz, D_2O) δ (ppm) No signals determined.

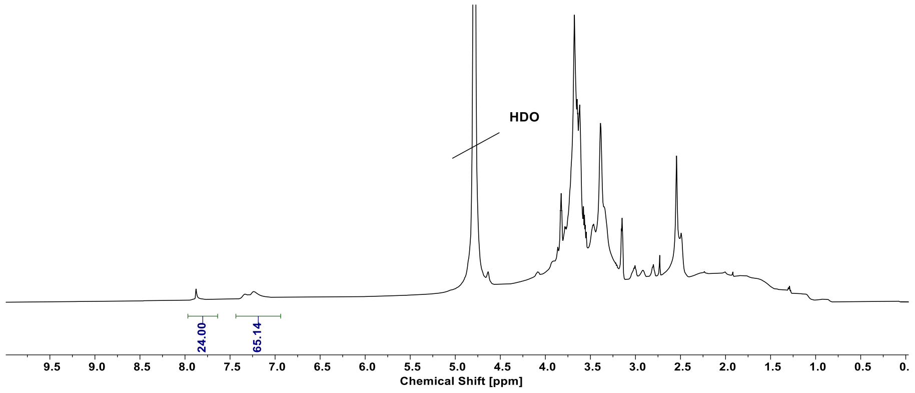

Figure S60: ^{1}H-NMR spectra of $_{22}\boldsymbol{B}^{2}_{29}$ *(**B1**) (600 MHz, D_2O).*

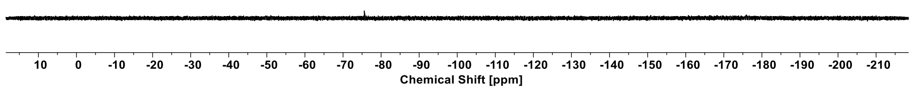

Figure S61: ^{19}F-NMR spectra of $_{22}\boldsymbol{B}^{2}_{29}$ *(**B1**) (564 MHz, D_2O).*

B2 **–** $(_{66}E^{2}_{29})$

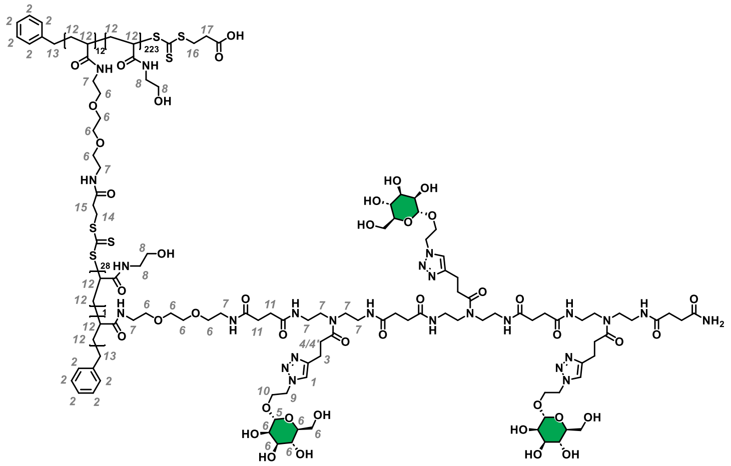

The synthesized brush² glycopolymer ***B2*** were obtained by the application of two steps: (1) *Attachment of the bridge molecule to arm polymers,* followed by *Cleavage of the protecting group of the conjugated bridge molecule* (2) *Attachment of the amine-activated arm polymers to the long polymer to gain brush² structures* described in "1.3 General Methods". 18 mg (0.32 µmol, 75.7 µmol related to the repetition unit) of p(PFPA) ***P3*** were functionalized with amine-preactivated arm polymer with comes from ***P8*** (5%, 23.6 mg, 3.8 µmol) quenched with ethanolamine and were received after dialysis as a white powder (15.79 mg, 5% incorporation, 0.17 µmol, 53% yield).

^{1}H-NMR (300 MHz, D_2O) δ (ppm) 7.89 (s, 36H, *H1*), 7.49 – 7.18 (m, 66H, *H2*), 4.67 – 4.55 (m, *H3*, overlap with water), 4.16 – 4.01 (m, *H4*), 4.01 – 0.00 (m, H4'-H17).

^{19}F-NMR (282 MHz, D_2O) δ (ppm) No signals determined.

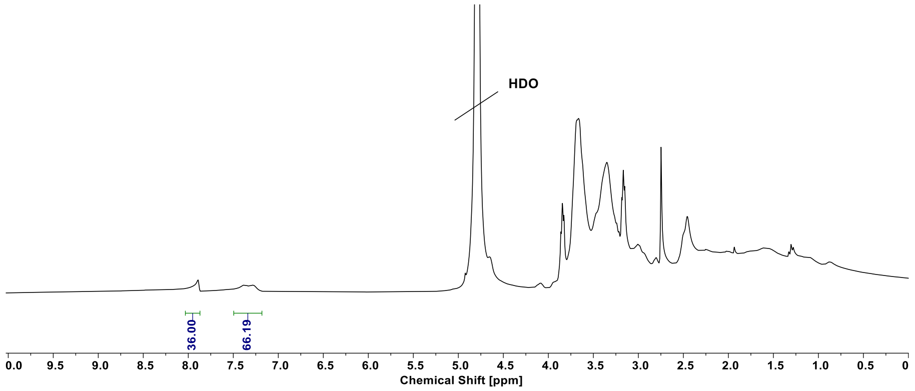

*Figure S62: ^{1}H-NMR spectra of $_{66}\boldsymbol{B}^{2}_{29}$ (**B2**) (300 MHz, D_2O).*

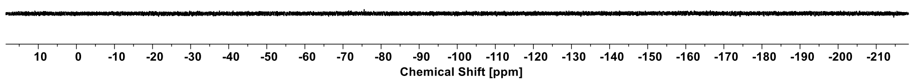

*Figure S63: ^{19}F-NMR spectra of $_{66}\boldsymbol{B}^{2}_{29}$ (**B2**) (282 MHz, D_2O).*

***B3* – ($_{22}I^2_{29}$)**

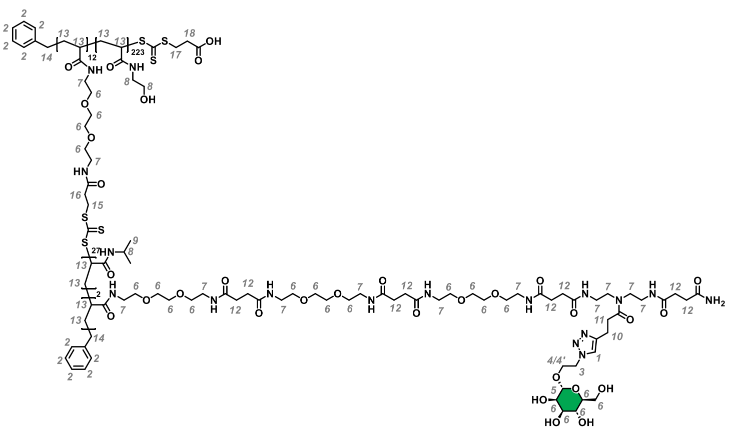

The synthesized brush² glycopolymer ***B3*** were obtained by the application of two steps: (1) *Attachment of the bridge molecule to arm polymers,* followed by *Cleavage of the protecting group of the conjugated bridge molecule* (2) *Attachment of the amine-activated arm polymers to the long polymer to gain brush² structures* described in "1.3 General Methods". 20 mg (0.36 µmol, 84.03 µmol related to the repetition unit) of p(PFPA) ***P3*** were functionalized with amine-preactivated arm polymer with comes from ***P9*** (5%, 25.60 mg, 4.20 µmol) quenched with ethanolamine and were received after dialysis as a white powder (26.75 mg, 5% incorporation, 0.27 µmol, 75% yield, slight impurities with ethanolamine).

^{1}H-NMR (300 MHz, D_2O) δ (ppm) 7.87 (s, 24H, *H1*), 7.60 – 7.07 (m, 65H, *H2*), 4.65 – 4.58 (m, *H3,* overlap with water peak), 4.11 – 3.76 (m, *H4, H4', H5, H8*), 3.76 – 3.51 (m, *H6*) 3.51 – 3.06 (m, *H7*), 3.06 – 2.96 (m, *H10*), 2.96- 2.84 (m, *H16*), 2.85 – 2.75 (m, *H11)*, 2.65 – 2.34 (m, *H12*), 1.26 – 0.95 (m, *H9*), 3.14 – 0.48 (m, *H13 - H15, H17, H18*).

^{19}F-NMR (282 MHz, D_2O) δ (ppm) No signals determined.

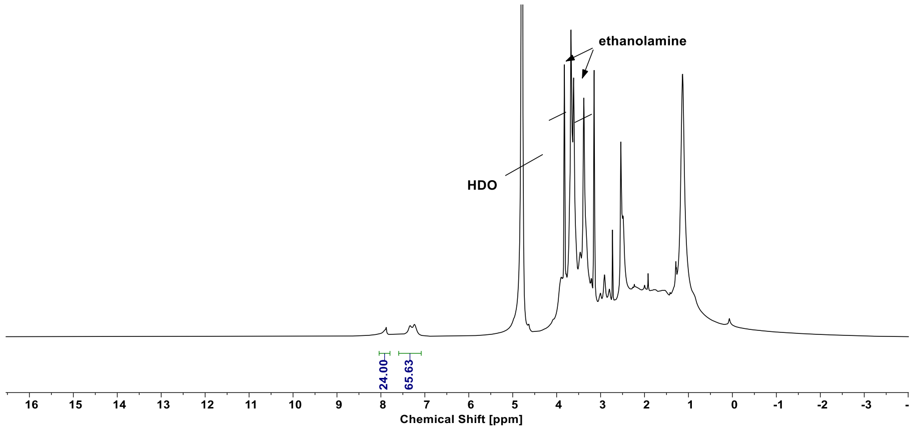

*Figure S64: ^{1}H-NMR spectra of $_{22}I^2_{29}$ (**B3**) (600 MHz, D_2O).*

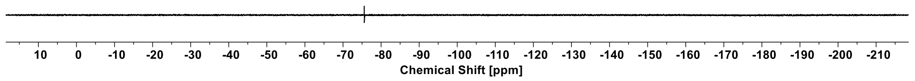

*Figure S65: ^{19}F-NMR spectra of $_{22}I^2_{29}$ (**B3**) (564 MHz, D_2O).*

***B4* – ($_{66}I^{2}_{29}$)**

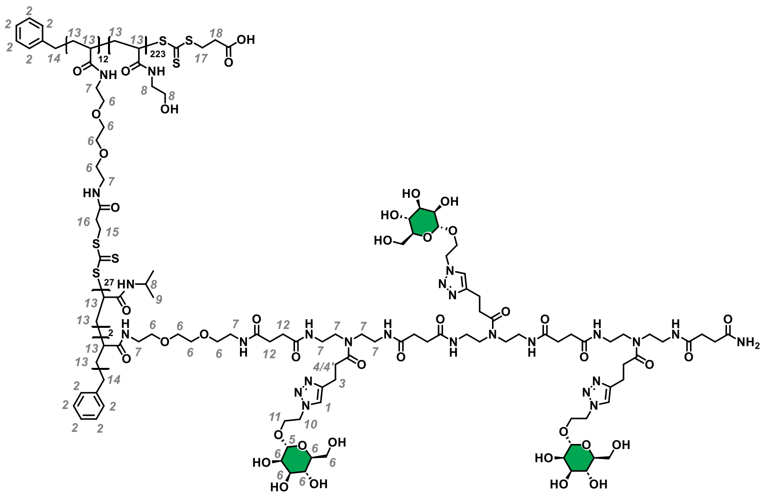

The synthesized brush² glycopolymer ***B4*** were obtained by the application of two steps: (1) *Attachment of the bridge molecule to arm polymers,* followed by *Cleavage of the protecting group of the conjugated bridge molecule* (2) *Attachment of the amine-activated arm polymers to the long polymer to gain brush² structures* described in "1.3 General Methods". 16 mg (0.28 µmol, 67.22 µmol related to the repetition unit) of p(PFPA) ***P3*** were functionalized with amine-preactivated arm polymer with comes from ***P10*** (5%, 24.30 mg, 3.36 µmol) quenched with ethanolamine and were received after dialysis as a white powder (20.10 mg, 5% incorporation, 0.18 µmol, 64% yield, slight impurities with ethanolamine).

^{1}H-NMR (300 MHz, D_2O) δ (ppm) 7.89 (s, 72H, *H1*), 7.48 – 7.17 (m, 67H, *H2*), 4.68 – 4.58 (m, *H3,* overlap with water peak), 4.32 – 3.10 (m, *H4, H4', H5 – H8)*, 3.09 – 2.87 (m, *H10, H16*), 2.87 – 2.71 (m, *H11*), 2.58 – 2.35 (m, *H12*), 1.28 – 0.83 (m, *H9*), 3.11 – 0.46 (m, *H13 – H15, H17, H18*).

^{19}F-NMR (282 MHz, D_2O) δ (ppm) No signals determined.

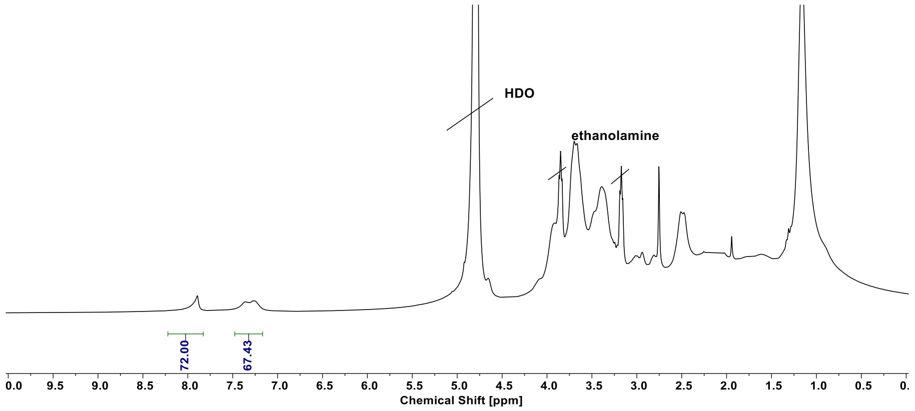

*Figure S66: ^{1}H-NMR spectra of $_{66}I^{2}_{29}$ (**B4**) (300 MHz, D_2O).*

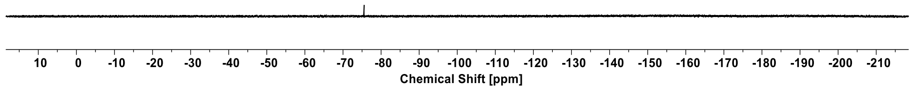

*Figure S67: ^{19}F-NMR spectra of $_{66}I^{2}_{29}$ (**B4**) (282 MHz, D_2O).*

***B5* – $({}_{66}\boldsymbol{E}^{2}_{57})$**

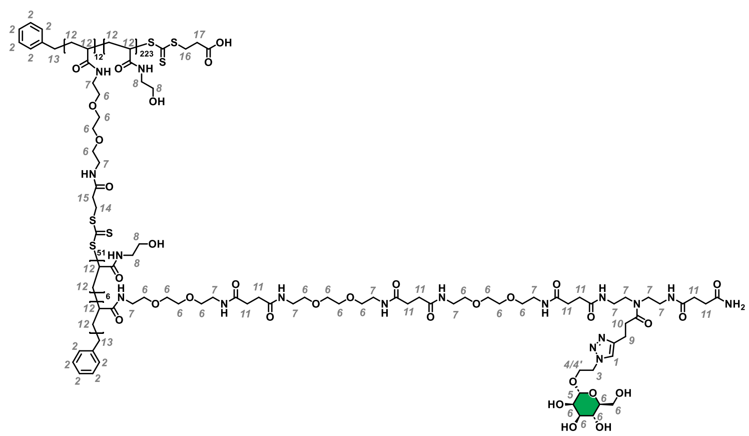

The synthesized brush² glycopolymer ***B5*** were obtained by the application of two steps: (1) *Attachment of the bridge molecule to arm polymers,* followed by *Cleavage of the protecting group of the conjugated bridge molecule* (2) *Attachment of the amine-activated arm polymers to the long polymer to gain brush² structures* described in "1.3 General Methods". 10 mg (0.18 µmol, 42.02 µmol related to the repetition unit) of p(PFPA) ***P3*** were functionalized with amine-preactivated arm polymer with comes from ***P11*** (5%, 29.44 mg, 2.10 µmol) quenched with ethanolamine and were received after dialysis as a white powder (26.11 mg, 5% incorporation, 0.13 µmol, 74% yield).

^{1}H-NMR (300 MHz, D_2O) δ (ppm) 7.88 (s, 72H, *H1*), 7.46 – 6.97 (m, 65H, *H2*), 4.65 – 4.58 (m, *H3,* overlap with water peak), 4.12 – 4.05 (m, *H4*), 3.95 – 3.10 (m, *H4', H5* - *H8*), 3.07 – 2.95 (m, *H9*), 2.95 – 2.85 (m, *H15*), 2.85 – 2.75 (m, *H10*), 2.70 – 2.38 (m, *H11*), 3.18 – 0.44 (m, *H12- H 14, H16, H17*).

^{19}F-NMR (282 MHz, D_2O) δ (ppm) No signals determined.

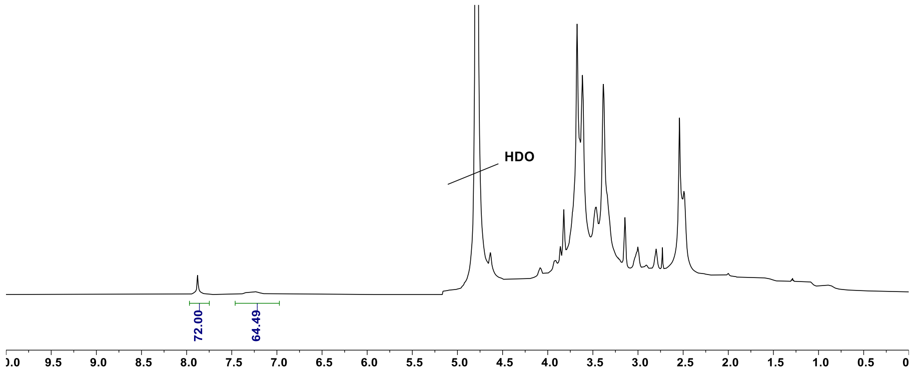

*Figure S68: ^{1}H-NMR spectra of ${}_{66}\boldsymbol{E}^{2}_{57}$ (**B5**) (600 MHz, D_2O).*

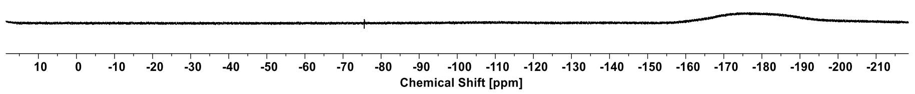

*Figure S69: ^{19}F-NMR spectra of ${}_{66}\boldsymbol{E}^{2}_{57}$ (**B5**) (564 MHz, D_2O).*

***B6* – ($_{198}E_{57}^{2}$)**

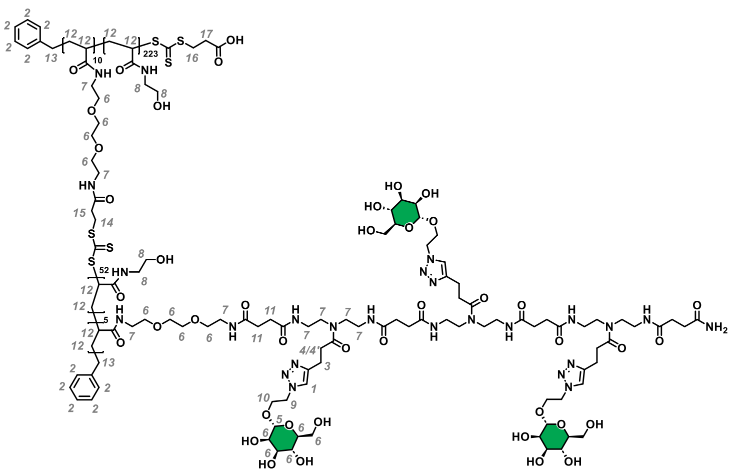

The synthesized brush² glycopolymer ***B6*** were obtained by the application of two steps: (1) *Attachment of the bridge molecule to arm polymers,* followed by *Cleavage of the protecting group of the conjugated bridge molecule* (2) *Attachment of the amine-activated arm polymers to the long polymer to gain brush² structures* described in "1.3 General Methods". 10 mg (0.18 µmol, 42.02 µmol related to the repetition unit) of p(PFPA) ***P3*** were functionalized with amine-preactivated arm polymer with comes from ***P12*** (5%, 32.98 mg, 2.10 µmol) quenched with ethanolamine and were received after dialysis as a white powder (26.15 mg, 4% incorporation, 0.14 µmol, 79% yield).

^{1}H-NMR (300 MHz, D_2O) δ (ppm) 7.88 (s, 3H, *H1*), 7.40 – 7.11 (m, 5H, *H2*), 4.68 – 4.53 (m, *H3,* overlap with water), 4.15 – 4.04 (m, *H4*), 3.99 – 3.81 (m, *H4', H5*), 3.81 – 3.12 (m, *H6 – H8*), 3.12 – 2.89 (m, *H9*), 2.88 – 2.72 (m, *H10*), 2.65 – 2.39 (m, *H11*), 3.12 – 0.68 (m, *H12 – H15*).

^{19}F-NMR (282 MHz, D_2O) δ (ppm) No signals determined.

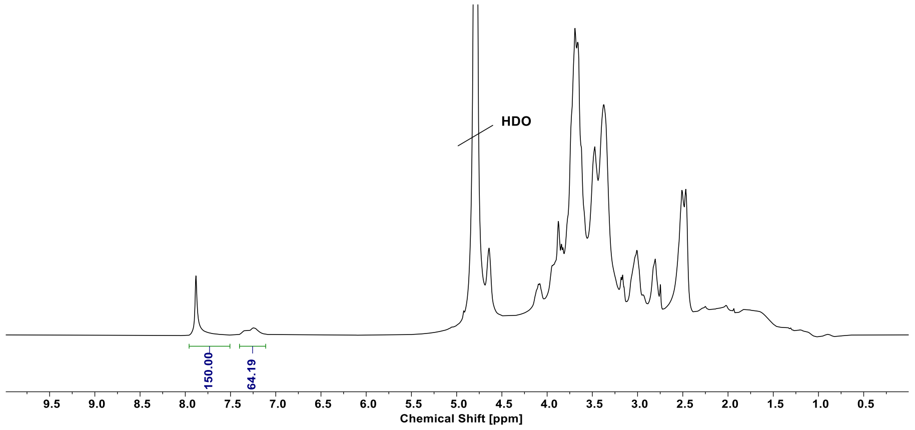

*Figure S70: ^{1}H-NMR spectra of $_{198}E_{57}^{2}$ (**B6**) (300 MHz, D_2O).*

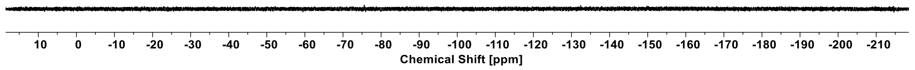

*Figure S71: ^{19}F-NMR spectra of $_{198}E_{57}^{2}$ (**B6**) (282 MHz, D_2O).*

***B7* – $\left({}_{66}I^{2}_{57}\right)$**

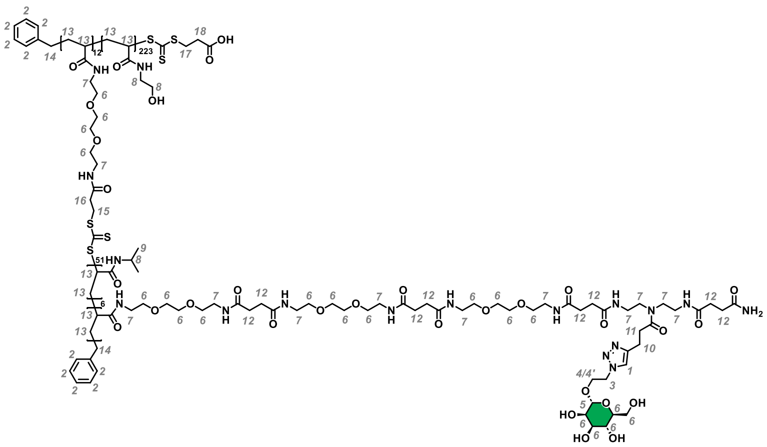

The synthesized brush² glycopolymer ***B7*** were obtained by the application of two steps: (1) *Attachment of the bridge molecule to arm polymers,* followed by *Cleavage of the protecting group of the conjugated bridge molecule* (2) *Attachment of the amine-activated arm polymers to the long polymer to gain brush² structures* described in "1.3 General Methods". 16 mg (0.28 µmol, 67.22 µmol related to the repetition unit) of p(PFPA) ***P3*** were functionalized with amine-preactivated arm polymer with comes from ***P13*** (5%, 46.77 mg, 3.36 µmol) quenched with ethanolamine and were received after dialysis as a white powder (32.35 mg, 5% incorporation, 0.17 µmol, 61% yield).

^{1}H-NMR (300 MHz, D_2O) δ (ppm) 7.88 (s, 72H, *H1*), 7.42 – 6.99 (m, 65H, *H2*), 4.66 – 4.57 (m, *H3,* overlap with water peak), 4.12 – 3.75 (m, *H4, H4', H5, H8*), 3.75 – 3.52 (m, *H6*) 3.52 – 3.08 (m, *H7*), 3.07 – 2.95 (m, *H10*), 2.95- 2.85 (m, *H16*), 2.85 – 2.76 (m, *H11)*, 2.64 – 2.32 (m, *H12*), 1.25 – 0.94 (m, *H9*), 3.18 – 0.23 (m, *H13 - H15, H17, H18*).

^{19}F-NMR (282 MHz, D_2O) δ (ppm) No signals determined.

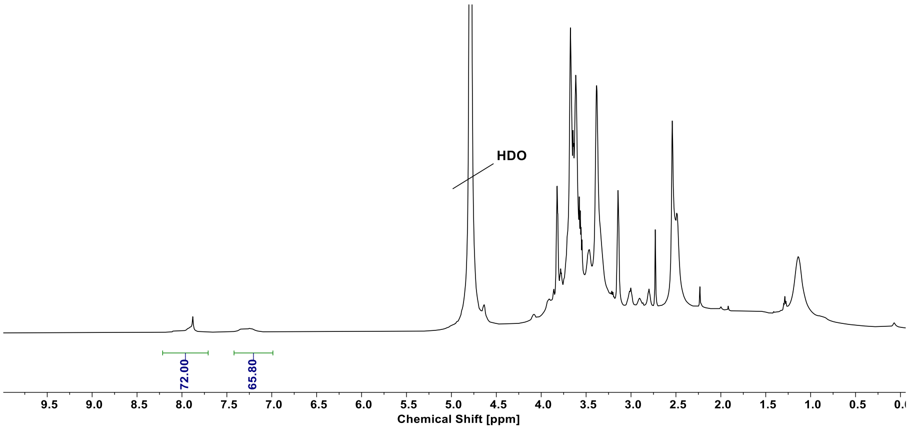

*Figure S72: ^{1}H-NMR spectra of ${}_{66}I^{2}_{57}$ (**B7**) (600 MHz, D_2O).*

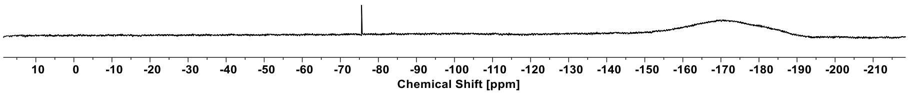

*Figure S73: ^{19}F-NMR spectra of ${}_{66}I^{2}_{57}$ (**B7**) (564 MHz, D_2O).*

***B8* – ($_{198}I^{2}_{57}$)**

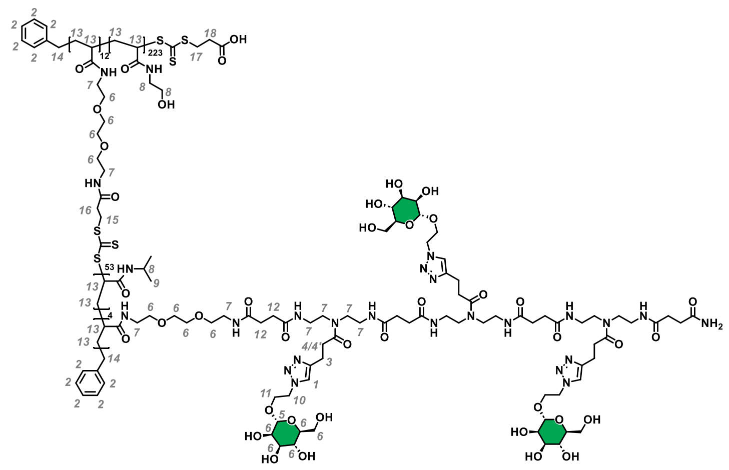

The synthesized brush² glycopolymer ***B7*** were obtained by the application of two steps: (1) *Attachment of the bridge molecule to arm polymers,* followed by *Cleavage of the protecting group of the conjugated bridge molecule* (2) *Attachment of the amine-activated arm polymers to the long polymer to gain brush² structures* described in "1.3 General Methods". 14 mg (0.25 µmol, 58.82 µmol related to the repetition unit) of p(PFPA) ***P3*** were functionalized with amine-preactivated arm polymer with comes from ***P14*** (5%, 40.77 mg, 2.94 µmol) quenched with ethanolamine and were received after dialysis as a white powder (31.76 mg, 5% incorporation, 0.17 µmol, 68% yield).

^{1}H-NMR (300 MHz, D_2O) δ (ppm) 7.88 (s, 114H, *H1*), 7.44 – 7.11 (m, 64H, *H2*), 4.66 – 4.54 (m, *H3,* overlap with water peak), 4.14 – 3.09 (m, *H4, H4', H5 – H8)*, 3.09 – 2.86 (m, *H10, H16*), 2.86 – 2.71 (m, *H11*), 2.59 – 2.36 (m, *H12*), 1.25 – 0.97 (m, *H9*), 3.10 – 0.00 (m, *H13 – H15, H17, H18*).

^{19}F-NMR (282 MHz, D_2O) δ (ppm) No signals determined.

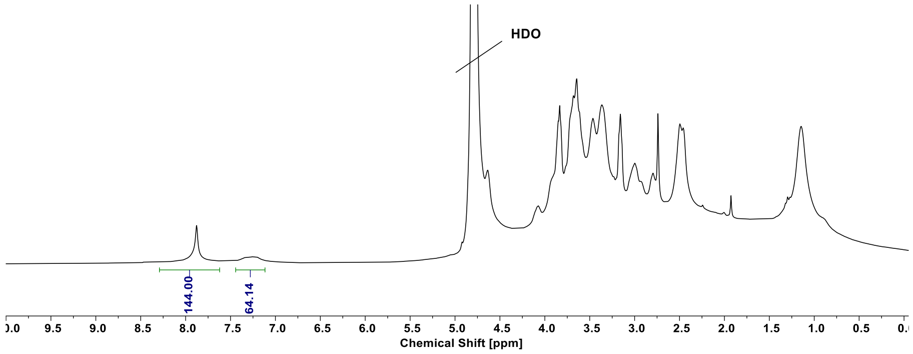

*Figure S74: ^{1}H-NMR spectra of $_{198}I^{2}_{57}$ (**B8**) (300 MHz, D_2O).*

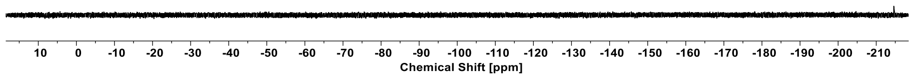

*Figure S75: ^{19}F-NMR spectra of $_{198}I^{2}_{57}$ (**B8**) (282 MHz, D_2O).*

***B9* – ($^{*}_{22}E^{2}_{29}$)**

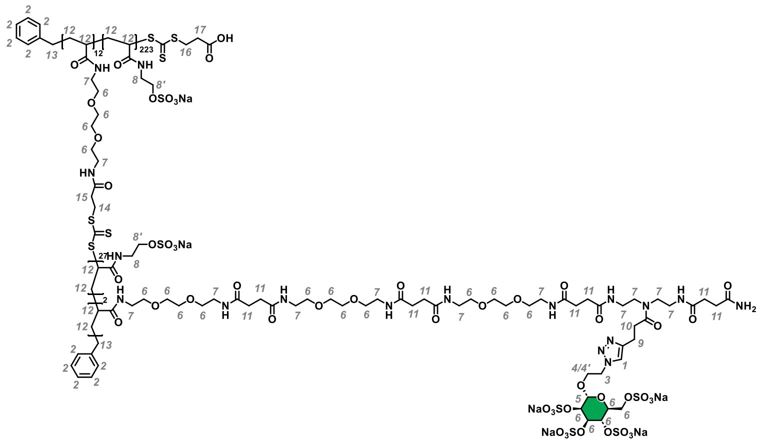

The synthesized polymer ***B9*** were obtained by the application of *standard protocol for global sulfation*, described in “1.3 General Methods”. 9.65 mg (0.097 µmol) of reference structure ***B1*** were sulfated and received after dialysis as a white powder (8.85 mg, 0.054 µmol assuming complete sulfation, 55% yield, degree of sulfation: ^{1}H-NMR - complete low field shift of e.g. the triazole peak by 0.05 ppm.

^{1}H-NMR (300 MHz, D_2O) δ (ppm) 7.93 (s, *H1*), 7.48 – 7.07 (m, *H2*), 5.19 – 5.11 (m, *H5,* overlap with water peak), 4.26 – 3.90 (m, *H8’*), 3.89 – 3.10 (m, *H6 - H8)*, 3.09 – 2.97 (m, *H9*), 2.97 – 2.88 (m, *H15*), 2.88 – 2.77 (m, *H10*), 2.63 – 2.39 (m, *H11*), 4.51 – 0.59 (m, *H3, H4, H4’, H12- H14, H16, H17*).

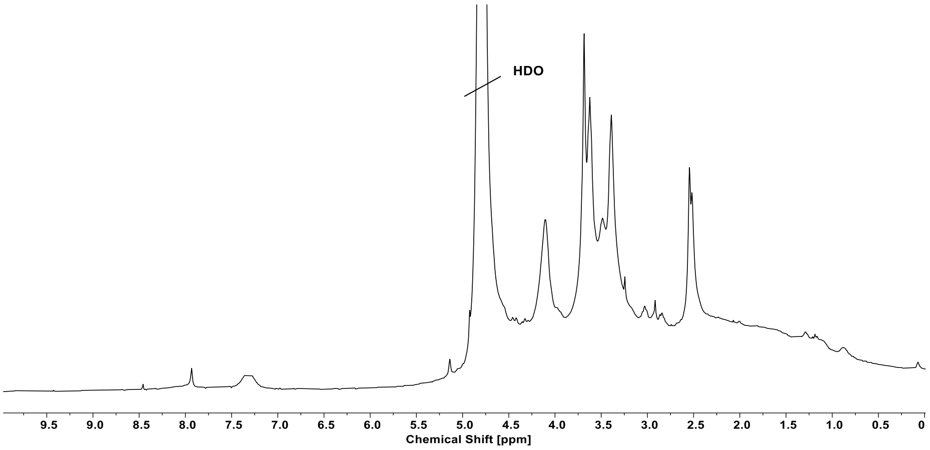

*Figure S76: ^{1}H-NMR spectra of sulfated $^{*}_{22}E^{2}_{29}$ (**B9**) (300 MHz, D_2O).*

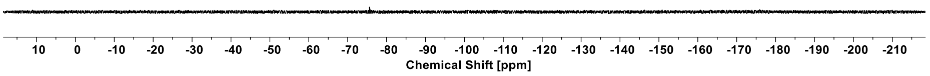

*Figure S77: ^{19}F-NMR spectra of sulfated $^{*}_{22}E^{2}_{29}$ (**B9**) (282 MHz, D_2O).*

***B10* – ($_{66}^{*}E_{29}^{2}$)**

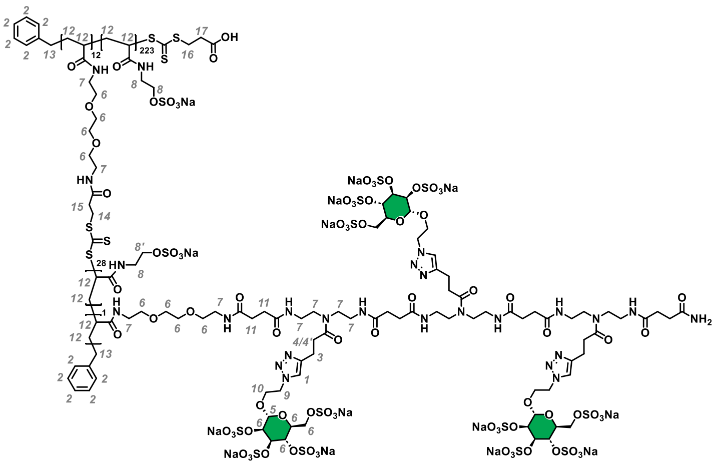

The synthesized polymer ***B10*** were obtained by the application of *standard protocol for global sulfation*, described in "1.3 General Methods". 8.0 mg (0.087 µmol) of reference structure ***B2*** were sulfated and received after dialysis as a white powder (10.44 mg, 0.064 µmol assuming complete sulfation, 74% yield, degree of sulfation: ^{1}H-NMR - complete low field shift of e.g. the triazole peak by 0.03 ppm.

^{1}H-NMR (300 MHz, D_2O) δ (ppm) 7.90 (s, *H1*), 7.41 – 7.12 (m, *H2*), 5.16 – 5.10 (m, *H5,* overlap with water peak), 4.26 – 3.02 (m, *H8'*), 3.08 – 2.97 (m, *H9*), 2.97 – 2.89 (m, *H15*), 2.89 – 2.79 (m, *H10*), 2.63 – 2.39 (m, *H11*), 4.63 – 0.79 (m. *H3, H4, H4', H12- H14, H16, H17*).

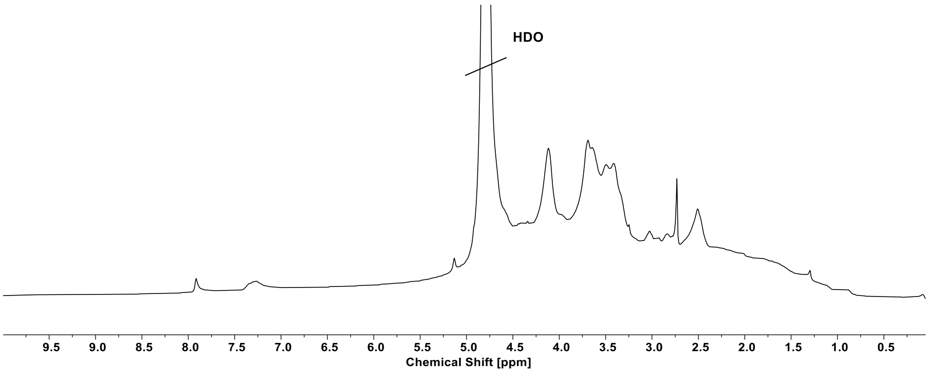

*Figure S78: ^{1}H-NMR spectra of sulfated $_{66}^{*}E_{29}^{2}$ (**B10**) (300 MHz, D_2O).*

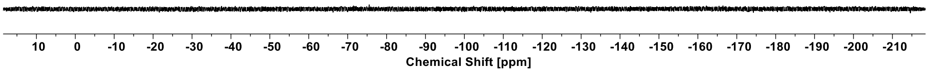

*Figure S79: ^{19}F-NMR spectra of sulfated $_{66}^{*}E_{29}^{2}$ (**B10**) (282 MHz, D_2O).*

***B11* – ($_{22}^{*}I_{29}^{2}$)**

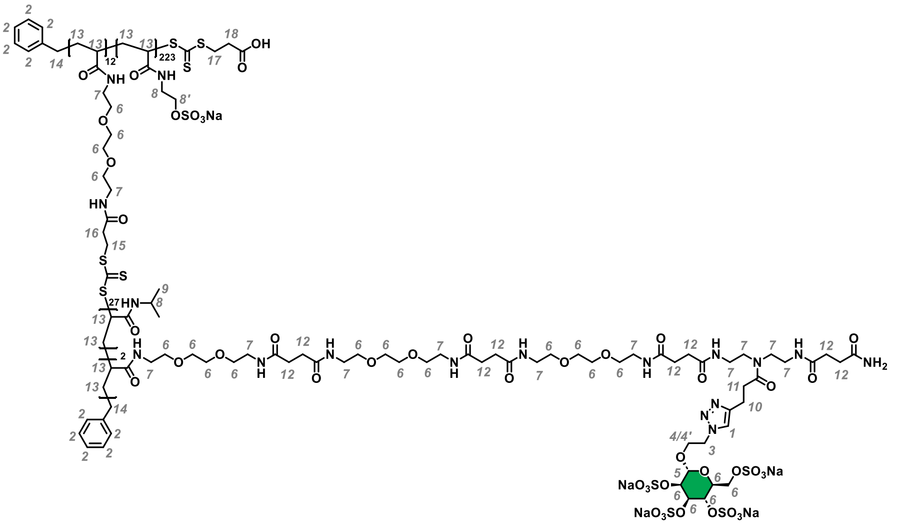

The synthesized polymer ***B11*** were obtained by the application of *standard protocol for global sulfation*, described in "1.3 General Methods". 12.9 mg (0.131 µmol) of reference structure ***B3*** were sulfated and received after dialysis as a white powder (11.17 mg, 0.085 µmol assuming complete sulfation, 65% yield, degree of sulfation: ^{1}H-NMR - complete low field shift of e.g. the triazole peak by 0.06 ppm.

^{1}H-NMR (300 MHz, D_2O) δ (ppm) 7.94 (s, *H1*), 7.49 – 7.09 (m, *H2*), 4.51 – 0.14 (m *H3*-*H18,* overlap with water peak).

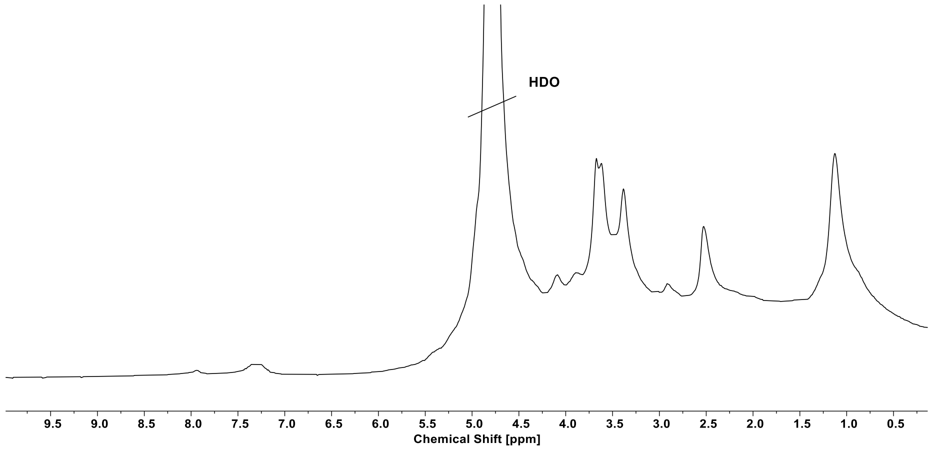

*Figure S80: ^{1}H-NMR spectra of sulfated $_{22}^{*}I_{29}^{2}$ (**B11**) (300 MHz, D_2O).*

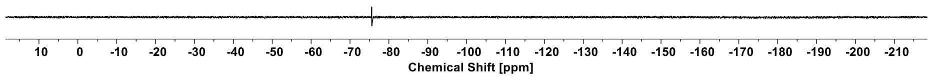

*Figure S81: ^{19}F-NMR spectra of sulfated $_{22}^{*}I_{29}^{2}$ (**B11**) (282 MHz, D_2O).*

***B12* – ($_{66}^{*}I_{29}^{2}$)**

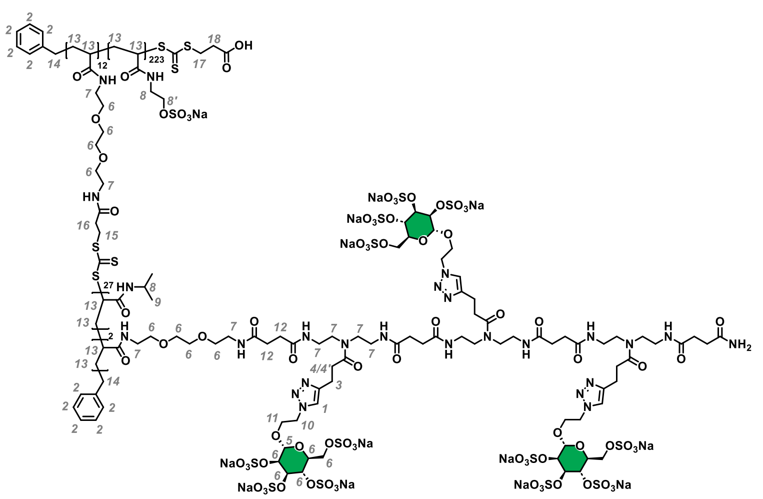

The synthesized polymer ***B12*** were obtained by the application of *standard protocol for global sulfation*, described in "1.3 General Methods". 10 mg (0.089 µmol) of reference structure ***B4*** were sulfated and received after dialysis as a white powder (12.4 mg, 0.075 µmol assuming complete sulfation, 84% yield, degree of sulfation: ^{1}H-NMR - complete low field shift of e.g. the triazole peak by 0.03 ppm.

^{1}H-NMR (300 MHz, D_2O) δ (ppm) 7.92 (s, *H1*), 7.43 – 7.13 (m, *H2*), 5.15 – 5.01 (m, *H5, overlap with water peak*), 4.68 – 3.12 (m, *H3, H4, H4', H5 – H8,* overlap with water peak), 3.07 – 2.98 (m, *H10*), 2.98 – 2.88 (m, *H16*), 2.88 – 2.79 (m, *H11*), 2.61 – 2.36 (m, *H12*), 1.28 – 0.95 (m, *H9*), 3.09 – 0.00 (m, *H13 – H15, H17, H18*).

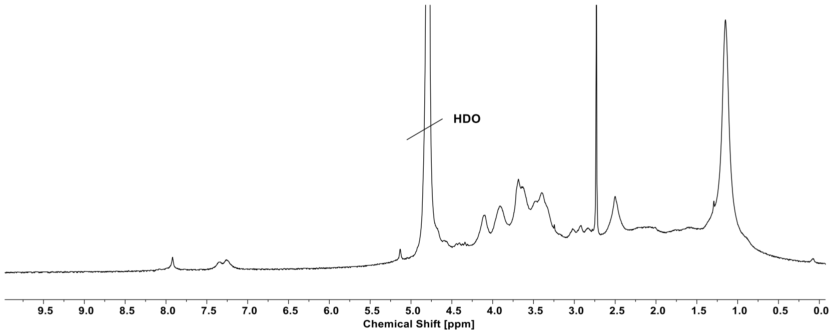

*Figure S82: ^{1}H-NMR spectra of sulfated $_{66}^{*}I_{29}^{2}$ (**B12**) (300 MHz, D_2O).*

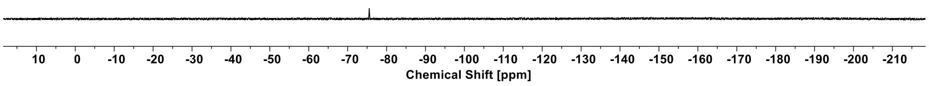

*Figure S83: ^{19}F-NMR spectra of sulfated $_{66}^{*}I_{29}^{2}$ (**B12**) (282 MHz, D_2O).*

***B13* – ($_{66}^{*}E_{57}^{2}$)**

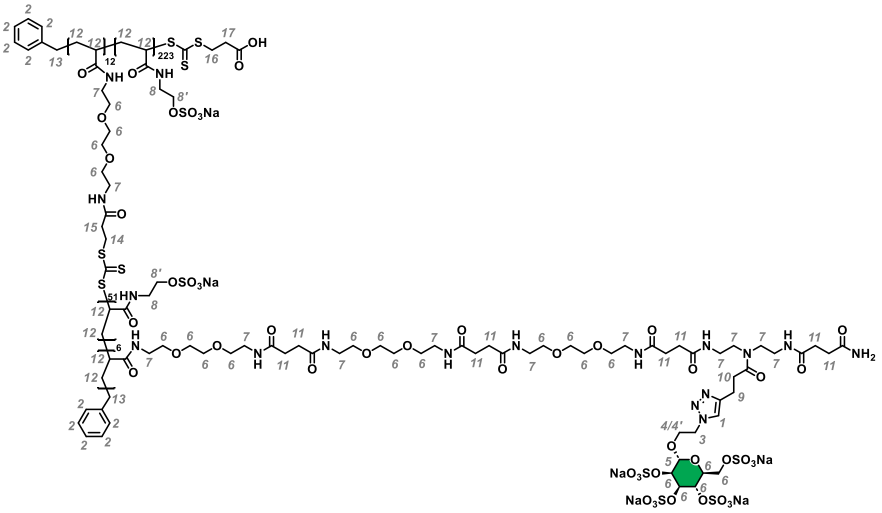

The synthesized polymer ***B13*** were obtained by the application of *standard protocol for global sulfation*, described in "1.3 General Methods". 13 mg (0.067 µmol) of reference structure ***B5*** were sulfated and received after dialysis as a white powder (13.36 mg, 0.043 µmol assuming complete sulfation, 64% yield, degree of sulfation: EA – 52% ^{1}H-NMR - complete low field shift of e.g. the triazole peak by 0.06 ppm.

^{1}H-NMR (300 MHz, D_2O) δ (ppm) 7.93 (s, *H1*), 7.42 – 7.09 (m, *H2*), 5.27 – 5.08 (m, *H5,* overlap with water peak), 4.24 – 4.00 (m, *H8'*), 3.88 – 3.11 (m, *H6 - H8)*, 3.09 – 2.97 (m, *H9*), 2.97 – 2.88 (m, *H15*), 2.88 – 2.77 (m, *H10*), 2.65 – 2.40 (m, *H11*), 4.18 – 0.52 (m, *H3, H4, H4', H12- H14, H16, H17*).

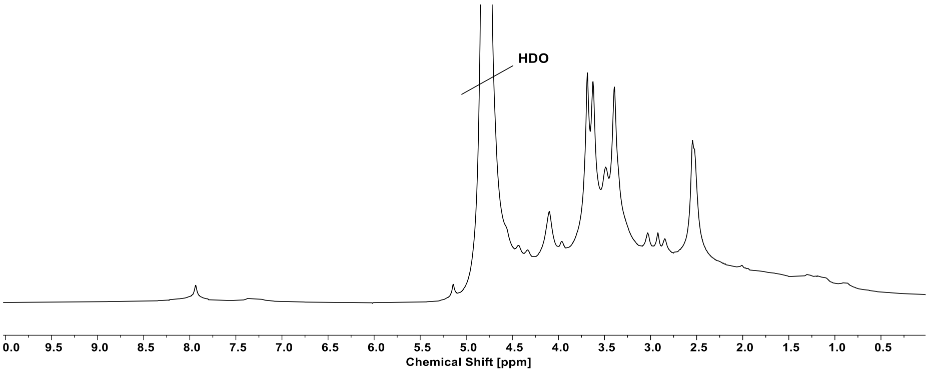

*Figure S84: ^{1}H-NMR spectra of sulfated $_{66}^{*}E_{57}^{2}$ (**B13**) (300 MHz, D_2O).*

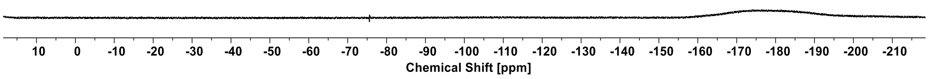

*Figure S85: ^{19}F-NMR spectra of sulfated $_{66}^{*}E_{57}^{2}$ (**B13**) (282 MHz, D_2O).*

B14 ***– (*** $_{198}^{*}E_{57}^{2}$***)***

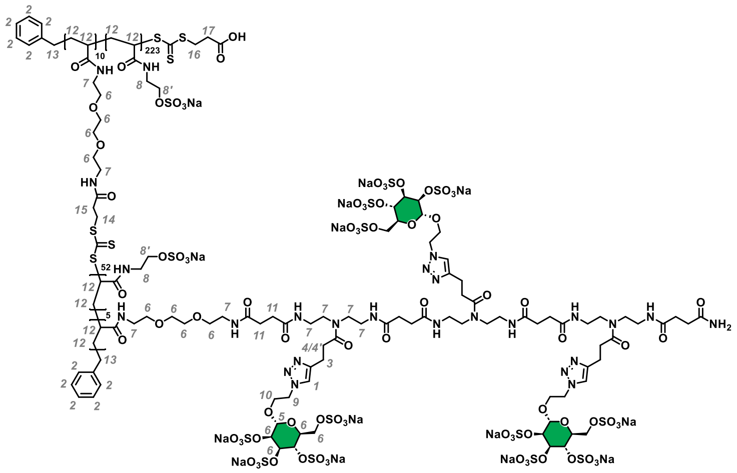

The synthesized polymer ***B14*** were obtained by the application of *standard protocol for global sulfation*, described in “1.3 General Methods”. 13 mg (0.071 µmol) of reference structure ***B6*** were sulfated and received after dialysis as a white powder (18.97 mg, 0.059 µmol assuming complete sulfation, 83% yield, degree of sulfation: ^{1}H-NMR - complete low field shift of e.g. the triazole peak by 0.04 ppm.

^{1}H-NMR (300 MHz, D_2O) δ (ppm) 7.92 (s, *H1*), 7.42 – 7.18 (m, *H2*), 5.17 – 5.10 (m, *H5,* overlap with water peak), 4.70 – 4.61 (m, *H3*, overlap with water), 4.21 – 4.02 (m, *H8’*), 4.62 – 0.00 (m, *H4, H4’ – H7, H8 - H17*).

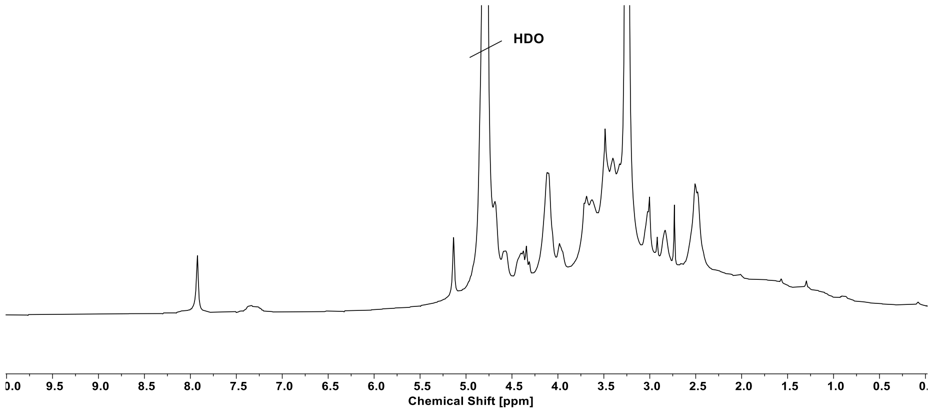

Figure S86: ^{1}*H-NMR spectra of sulfated* $_{198}^{*}E_{57}^{2}$ *(**B14**) (300 MHz,* D_2O*).*

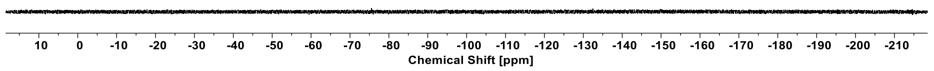

Figure S87: ^{19}*F-NMR spectra of sulfated* $_{198}^{*}E_{57}^{2}$ *(**B14**) (282 MHz,* D_2O*).*

***B15* – (${}_{66}^{*}\boldsymbol{I}_{57}^{2}$)**

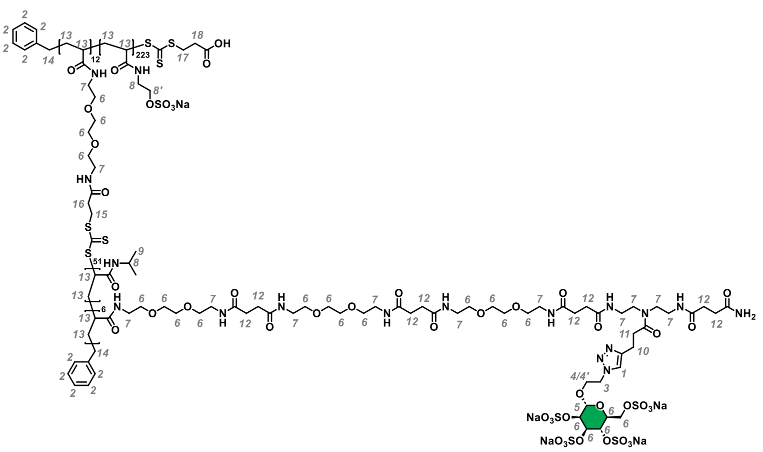

The synthesized polymer ***B15*** were obtained by the application of *standard protocol for global sulfation*, described in “1.3 General Methods”. 14.86 mg (0.077 µmol) of reference structure ***B7*** were sulfated and received after dialysis as a white powder (12.75 mg, 0.052 µmol assuming complete sulfation, 68% yield, degree of sulfation: ^{1}H-NMR - complete low field shift of e.g. the triazole peak by 0.06 ppm.

^{1}H-NMR (300 MHz, D_2O) δ (ppm) 7.93 (s, *H1*), 7.47 – 7.12 (m, *H2*), 4.36 – 0.19 (m *H3-H18,* overlap with water peak).

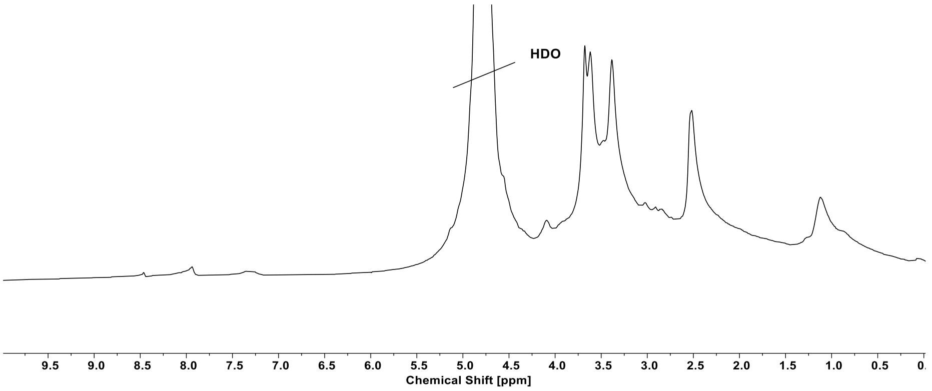

*Figure S88: ^{1}H-NMR spectra of sulfated ${}_{66}^{*}\boldsymbol{I}_{57}^{2}$ (**B15**) (300 MHz, D_2O).*

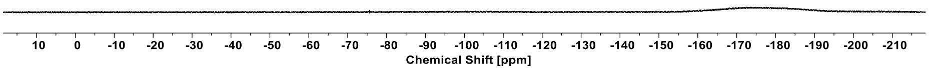

*Figure S89: ^{19}F-NMR spectra of sulfated ${}_{66}^{*}\boldsymbol{I}_{57}^{2}$ (**B15**) (282 MHz, D_2O).*

***B16* – ($_{198}^{*}I_{57}^{2}$)**

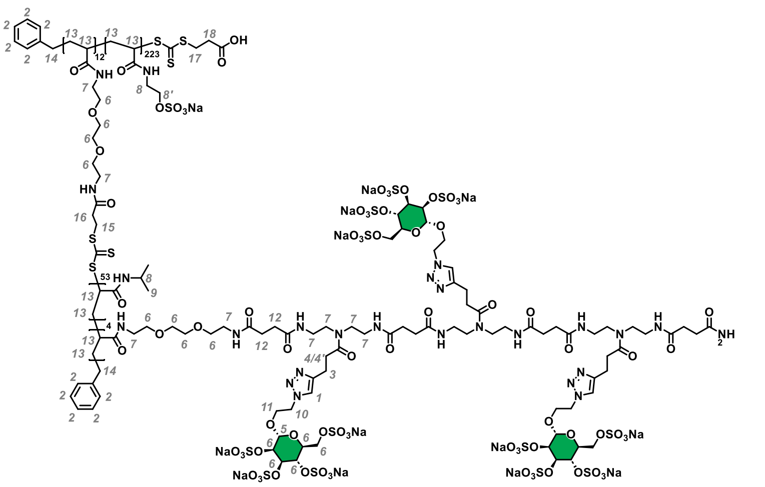

The synthesized polymer ***B16*** were obtained by the application of *standard protocol for global sulfation*, described in "1.3 General Methods". 15 mg (0.078 µmol) of reference structure ***B8*** were sulfated and received after dialysis as a white powder (19.54 mg, 0.072 µmol assuming complete sulfation, 92% yield, degree of sulfation: ^{1}H-NMR - complete low field shift of e.g. the triazole peak by 0.05 ppm.

^{1}H-NMR (300 MHz, D_2O) δ (ppm) 7.92 (s, *H1*), 7.47 – 7.11 (m, *H2*), 5.16 – 5.10 (m, *H5, overlap with water*), 4.48 – 0.00 (m *H3-H18,* overlap with water peak).

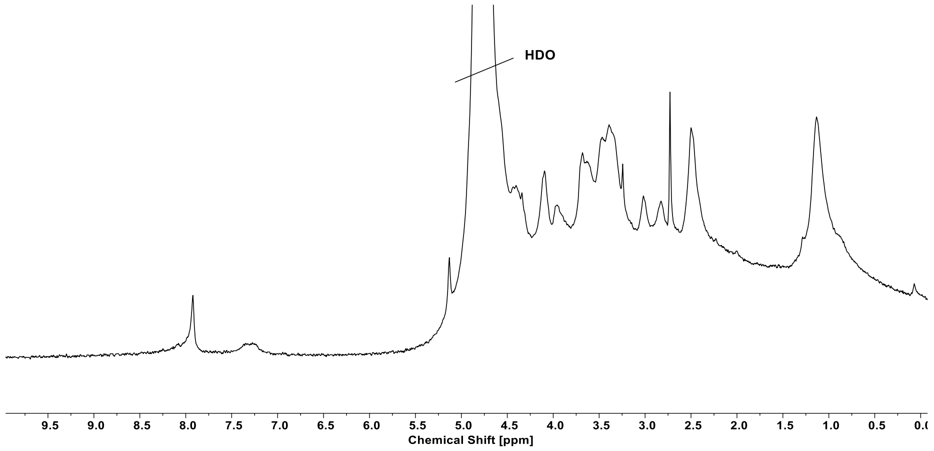

*Figure S90: ^{1}H-NMR spectra of sulfated $_{198}^{*}I_{57}^{2}$ (**B15**) (300 MHz, D_2O).*

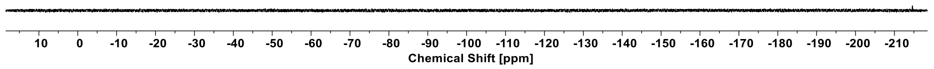

*Figure S91: ^{19}F-NMR spectra of sulfated $_{198}^{*}I_{57}^{2}$ (**B15**) (282 MHz, D_2O).*

2.6 Synthesis of reference structures P15–P24 and B17

***P15* – ($_{11}E_{235}$)**

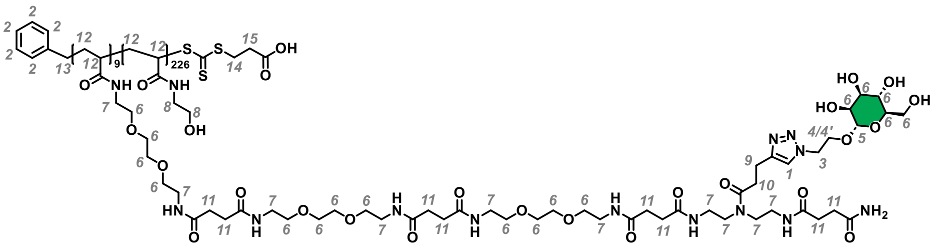

The synthesized polymer ***P15*** were obtained by the application of *synthesis of brush-like glycopolymers (arm-structures) and linear reference structures*, described in “1.3 General Methods”. 50 mg (0.89 µmol, 210.1 µmol related to the repetition unit) of p(PFPA) ***P3*** were functionalized with oligoamidoamine ***O1*** (5%, 13.85 mg, 10.5 µmol) quenched with ethanolamine and were received after dialysis as a white powder (31.52 mg, 4% incorporation, 0.84 µmol, 94% yield).

^{1}H-NMR (300 MHz, D_2O) δ (ppm) 7.88 (s, 9H, *H1*), 7.44 – 7.14 (m, 5H, *H2*), 4.67 – 4.59 (m, *H3,* overlap with water peak), 4.14 – 3.08 (m, *H4, H4’, H5* - *H8*), 3.08 – 0.77 (m, *H9* – *H15*).

^{19}F-NMR (282 MHz, D_2O) δ (ppm) No signals determined.

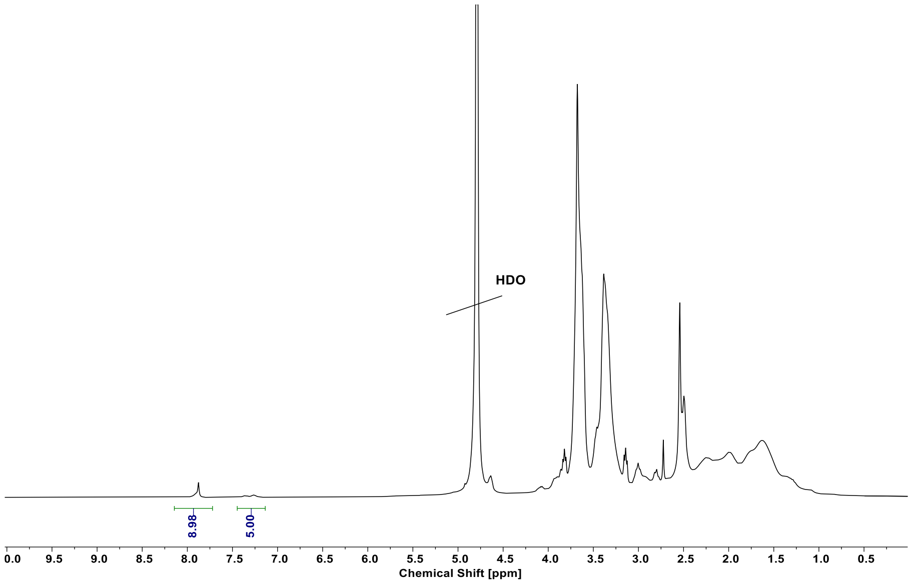

*Figure S92: ^{1}H-NMR spectra of $_{11}E_{235}$ (**P15**) (300 MHz, D_2O).*

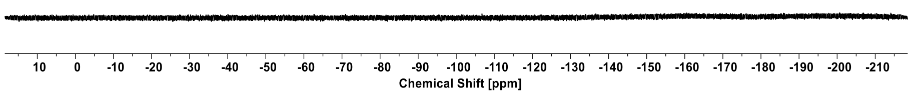

*Figure S93: ^{19}F-NMR spectra of $_{11}E_{235}$ (**P15**) (282 MHz, D_2O).*

P16 – $({}_{33}E_{235})$

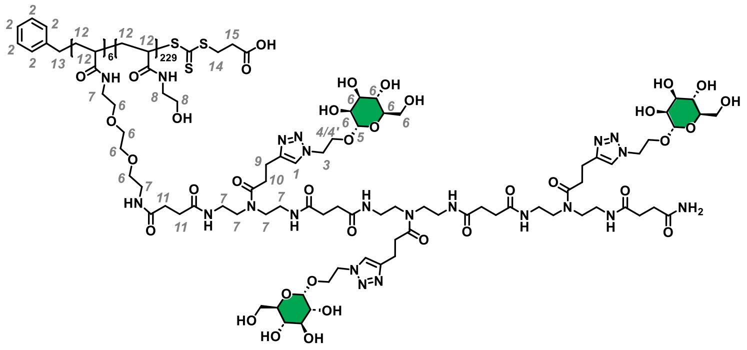

The synthesized polymer ***P16*** were obtained by the application of *synthesis of brush-like glycopolymers (arm-structures) and linear reference structures*, described in "1.3 General Methods". 50 mg (0.89 µmol, 210.1 µmol related to the repetition unit) of p(PFPA) ***P3*** were functionalized with oligoamidoamine ***O2*** (5%, 19.84 mg, 10.5 µmol) quenched with ethanolamine and were received after dialysis as a white powder (27.25 mg, 3% incorporation, 0.72 µmol, 81% yield).

^{1}H-NMR (300 MHz, D_2O) δ (ppm) 7.87 (s, 3H, *H1*), 7.43 – 7.16 (m, 5H, *H2*), 4.68 – 4.52 (m, *H3,* overlap with water peak), 4.15 – 4.01 (m, *H4*), 4.01 – 3.09 (m, *H4', H5 - H8*), 3.07 – 2.86 (m, *H9*), 2.86 – 2.70 (m, *H10*), 2.58 – 2.37 (m, *H11*), 3.08 – 0.70 (m, *H12 – H15*).

^{19}F-NMR (282 MHz, D_2O) δ (ppm) No signals determined.

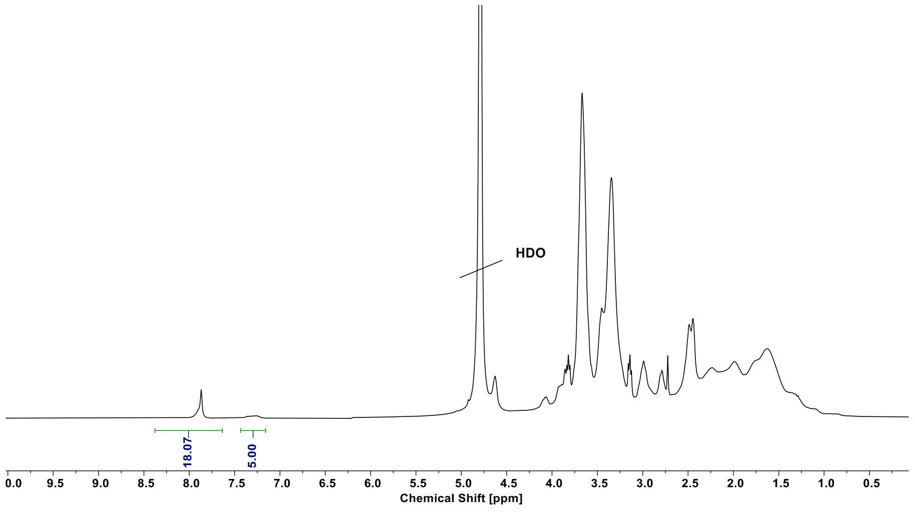

*Figure S94: ^{1}H-NMR spectra of ${}_{33}E_{235}$ (**P16**) (300 MHz, D_2O).*

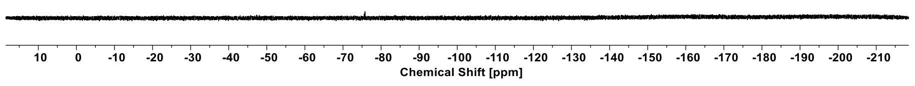

*Figure S95: ^{19}F-NMR spectra of ${}_{33}E_{235}$ (**P16**) (282 MHz, D_2O).*

***P17* – ($_{11}I_{235}$)**

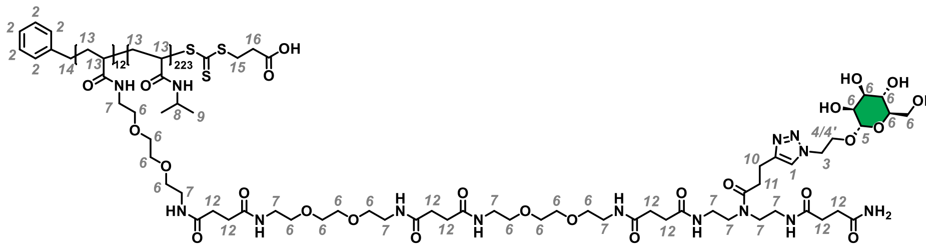

The synthesized polymer ***P17*** were obtained by the application of *synthesis of brush-like glycopolymers (arm-structures) and linear reference structures*, described in "1.3 General Methods". 50 mg (0.89 µmol, 210.1 µmol related to the repetition unit) of p(PFPA) ***P3*** were functionalized with oligoamidoamine ***O1*** (5%, 13.84 mg, 10.5µmol) quenched with isopropylamine and were received after dialysis as a white powder (26.62 mg, 5% incorporation, 0.65 µmol, 73% yield).

^{1}H-NMR (300 MHz, D_2O) δ (ppm) 7.88 (s, 2H, *H1*), 7.44 – 7.11 (m, 5H, *H2*), 4.68 – 4.57 (m, *H3,* overlap with water peak), 4.20 – 3.79 (m, *H4, H4', H5, H8*), 3.78 – 3.54 (m, *H6*) 3.54 – 3.28 (m, *H7, H8*), 3.08 – 2.95 (m, *H10*), 2.88 – 2.74 (m, *H11)*, 2.62 – 2.41 (m, *H12*), 1.25 – 0.95 (m, *H9*), 3.12– 0.49 (m, *H13- H16*).

^{19}F-NMR (282 MHz, D_2O) δ (ppm) No signals determined.

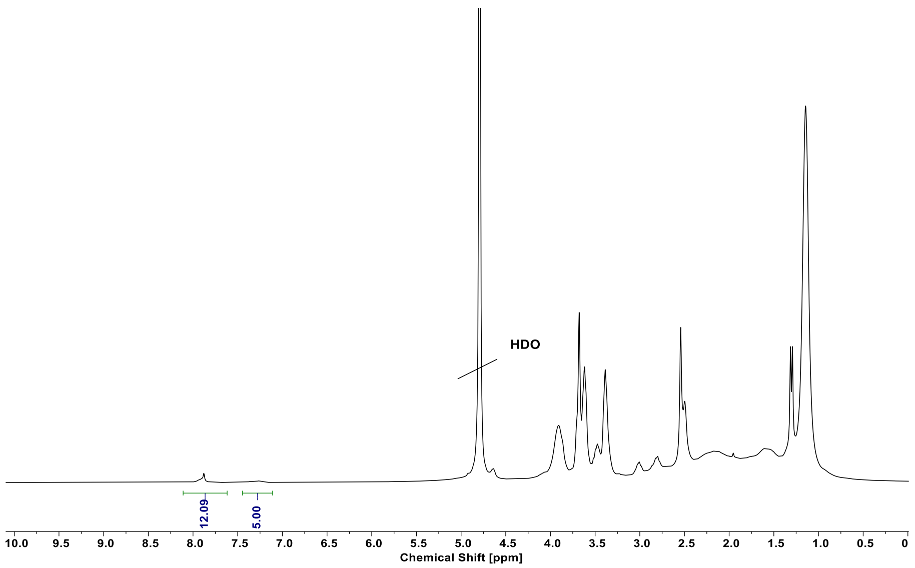

*Figure S96: ^{1}H-NMR spectra of $_{11}I_{235}$ (**P17**) (300 MHz, D_2O).*

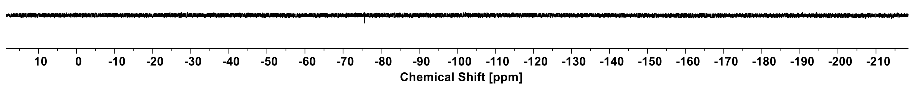

*Figure S97: ^{19}F-NMR spectra of $_{11}I_{235}$ (**P17**) (282 MHz, D_2O).*

***P18* – ($_{33}\boldsymbol{I}_{235}$)**

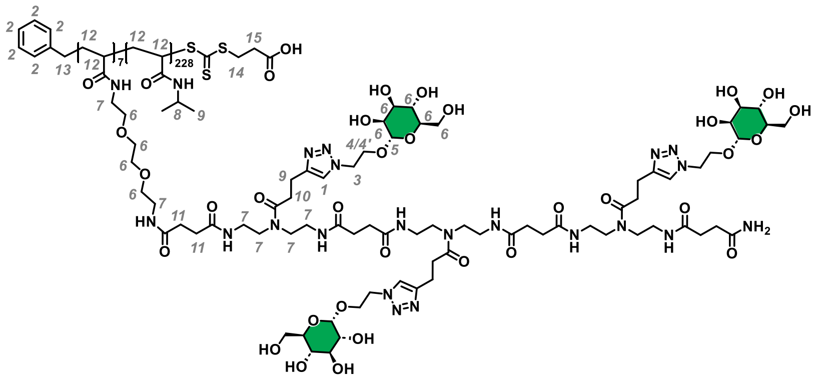

The synthesized polymer ***P18*** were obtained by the application of *synthesis of brush-like glycopolymers (arm-structures) and linear reference structures*, described in "1.3 General Methods". 50 mg (0.89 µmol, 210.1 µmol related to the repetition unit) of p(PFPA) ***P3*** were functionalized with oligoamidoamine ***O2*** (5%, 19.84 mg, 10.5 µmol) quenched with isopropylamine and were received after dialysis as a white powder (28.75 mg, 3% incorporation, 0.74 µmol, 83% yield).

^{1}H-NMR (300 MHz, D_2O) δ (ppm) 7.87 (s, 21H, *H1*), 7.43 – 7.16 (m, 5H, *H2*), 4.68 – 4.55 (m, *H3,* overlap with water peak), 4.13 – 3.79 (m, *H4, H4', H5, H8*), 3.79 – 3.55 (m, *H6*), 3.55 – 3.17 (m, *H7*), 3.06 – 2.90 (m, *H9*), 2.90 – 2.70 (m, *H10*), 2.56 – 2.35 (m, *H11*), 1.25 – 0.99 (m, *H9*), 3.14 – 0.55 (m, *H12 – H15*).

^{19}F-NMR (282 MHz, D_2O) δ (ppm) No signals determined.

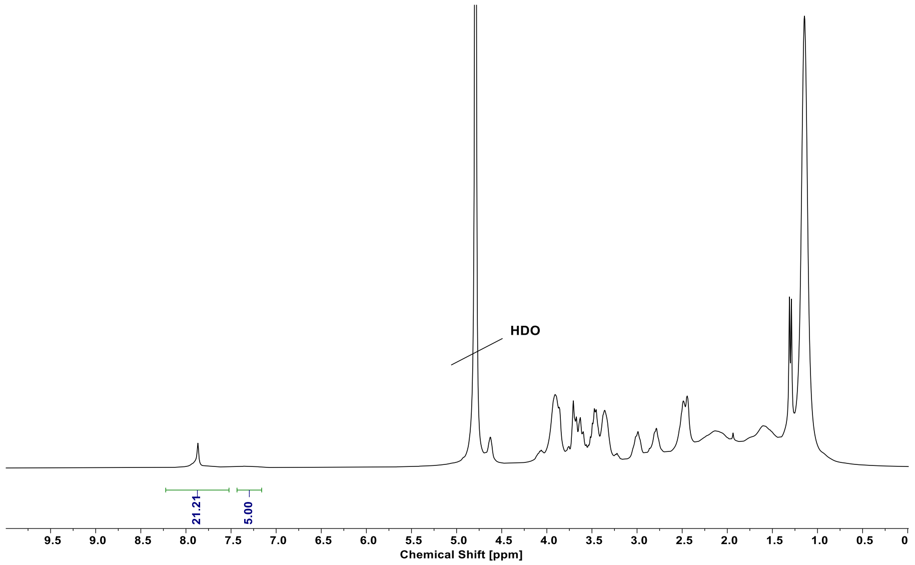

*Figure S98: ^{1}H-NMR spectra of $_{33}\boldsymbol{I}_{235}$ (**P18**) (300 MHz, D_2O).*

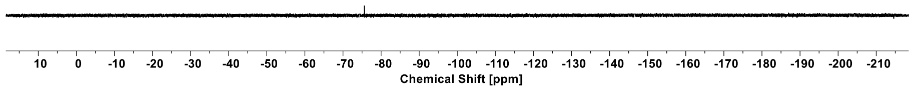

*Figure S99: ^{19}F-NMR spectra of $_{33}\boldsymbol{I}_{235}$ (**P18**) (282 MHz, D_2O).*

***P19* – ($_{11}^{*}E_{235}$)**

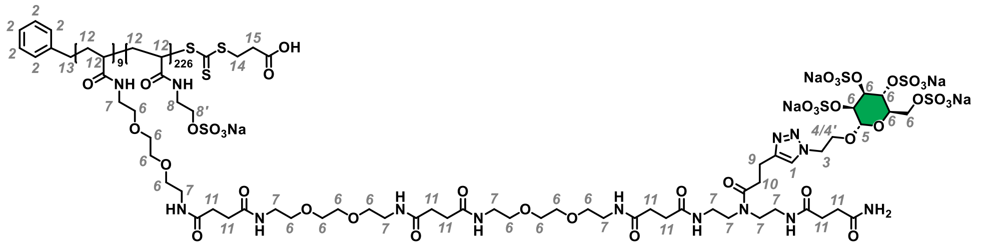

The synthesized polymer ***P19*** were obtained by the application of *standard protocol for global sulfation*, described in "1.3 General Methods". 13.04 mg (0.35 µmol) of reference structure ***P15*** were sulfated and received after dialysis as a white powder (11.36 mg, 0.18 µmol assuming complete sulfation, 51% yield, degree of sulfation: ^{1}H-NMR - complete low field shift of e.g. the triazole peak (1) by 0.06 ppm or the ethanolamine side chain signals 8' by 0.45 ppm and 8 by 0.13 ppm).

^{1}H-NMR (300 MHz, D_2O) δ (ppm) 7.93 (s, *H1*), 7.41 – 7.14 (m, *H2*), 5.16 – 5.11 (m, *H5*), 4.71 – 3.17 (m, H3, *H4, H4', H6 – H8,* overlap with water peak), 4.27 – 4.01 (m, *H8'*), 3.11 – 2.96 (m, *H9*), 2.89 – 2.78 (m, *H10*), 3.19 – 0.80 (m, *H11* – *H15*).

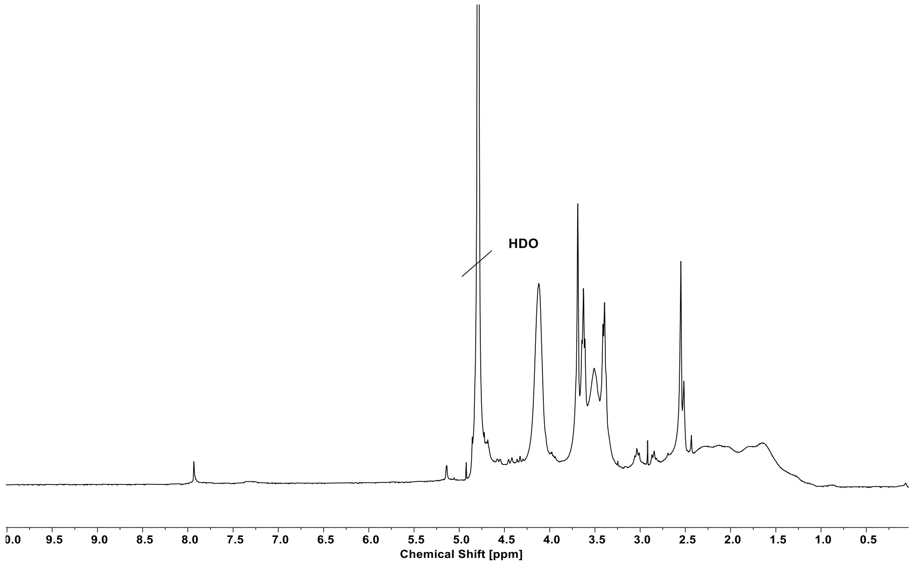

*Figure S100: ^{1}H-NMR spectra of sulfated $_{11}^{*}E_{235}$ (**P19**) (300 MHz, D_2O).*

***P20* – $({}_{33}^{*}E_{235})$**

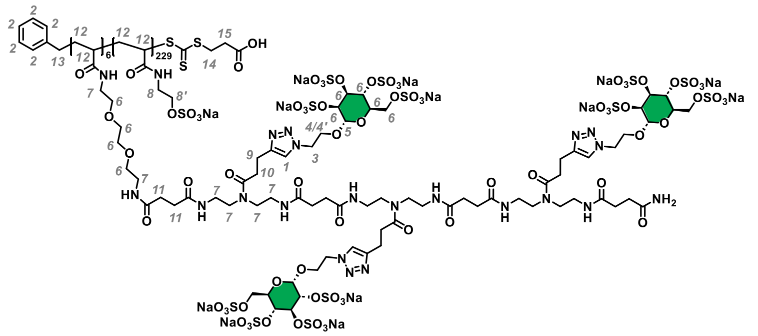

The synthesized polymer ***P20*** were obtained by the application of *standard protocol for global sulfation*, described in “1.3 General Methods”. 9.55 mg (0.25 µmol) of reference structure ***P19*** were sulfated and received after dialysis as a white powder (10.0 mg, 0.15 µmol assuming complete sulfation, 56% yield, degree of sulfation: ^{1}H-NMR - complete low field shift of e.g. the triazole peak by 0.05 ppm. **^{1}H-NMR** (300 MHz, D_2O) δ (ppm) 7.92 (s, *H1*), 7.41 – 7.18 (m, *H2*), 5.16 – 5.11 (m, *H5*), 4.71 – 3.22 (m, *H3, H4, H4’, H6 – H8,* overlap with water peak), 4.27 – 4.01 (m, *H8’*), 3.08 – 2.94 (m, *H9*), 2.94 – 2.79 (m, *H10*), 3.19 – 0.77 (m, *H11 – H15*).

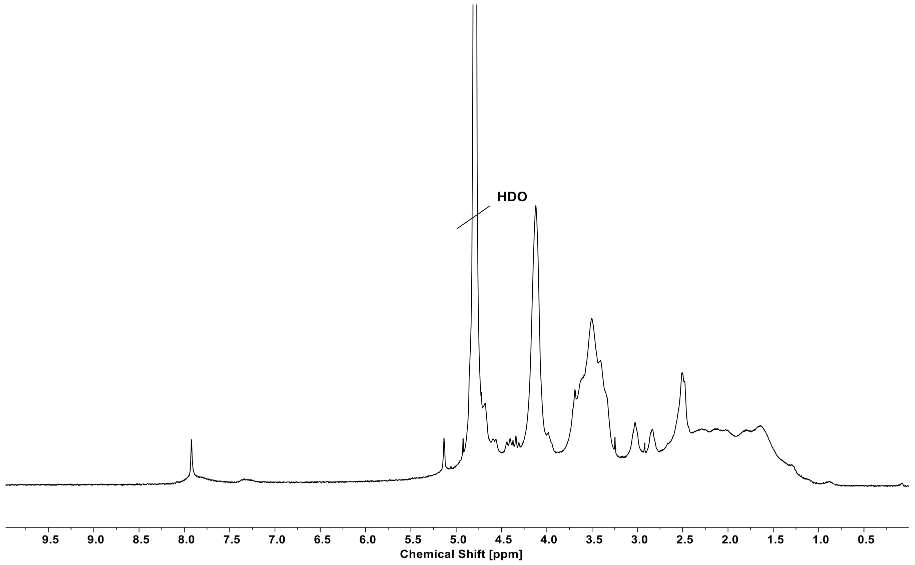

*Figure S101: ^{1}H-NMR spectra of sulfated ${}_{33}^{*}\boldsymbol{E}_{235}$ (**P20**) (300 MHz, D_2O).*

P21 – ($_{11}^{*}I_{235}$)

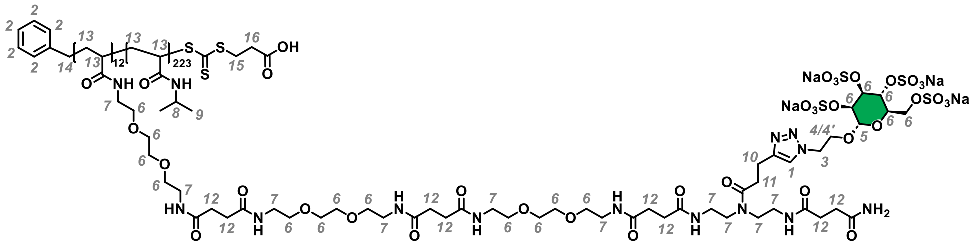

The synthesized polymer ***P21*** were obtained by the application of *standard protocol for global sulfation*, described in "1.3 General Methods". 13.04 mg (0.32 µmol) of reference structure ***P17*** were sulfated and received after dialysis as a white powder (11.07 mg, 0.24 µmol assuming complete sulfation, 75% yield, degree of sulfation: ^{1}H-NMR - complete low field shift of e.g. the triazole peak by 0.06 ppm.

^{1}H-NMR (300 MHz, D_2O) δ (ppm) 7.93 (s, *H1*), 7.44 – 7.12 (m, *H2*), 5.18 – 5.10 (m, *H5*), 4.72 – 3.77 (m, *H3, H4, H4' H6,* overlap with water peak), 4.04 – 3.76 (m, *H8*), 3.77 – 3.54 (m, *H6*), 3.54 – 3.23 (m, *H7*), 3.09 – 2.98 (m, *H10*), 2.89 – 2.76 (m, *H11)*, 2.62 – 2.42 (m, *H12*), 1.26 – 0.85 (m, *H9*), 3.16– 0.55 (m, *H13- H16*).

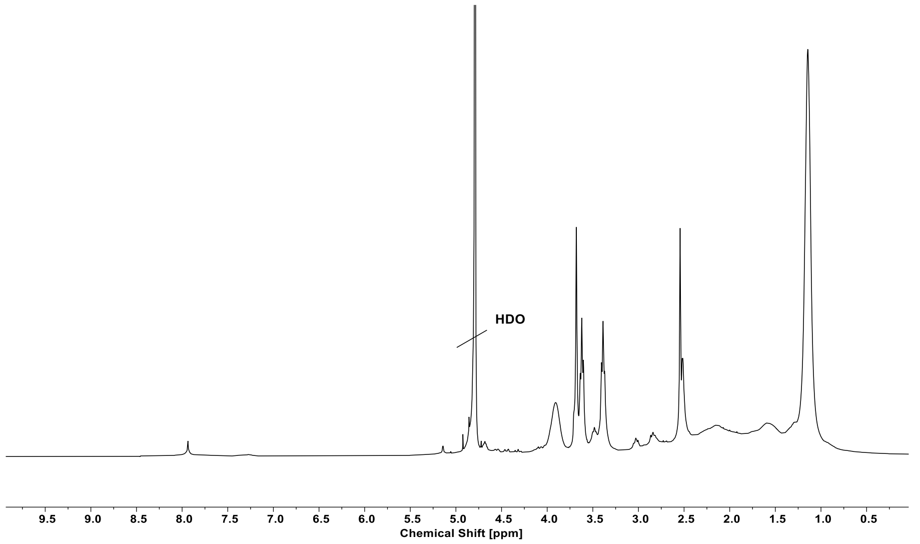

*Figure S102: ^{1}H-NMR spectra of sulfated $_{11}^{*}I_{235}$ **(P21)** (300 MHz, D_2O).*

***P22* – ($_{33}^{*}I_{235}$)**

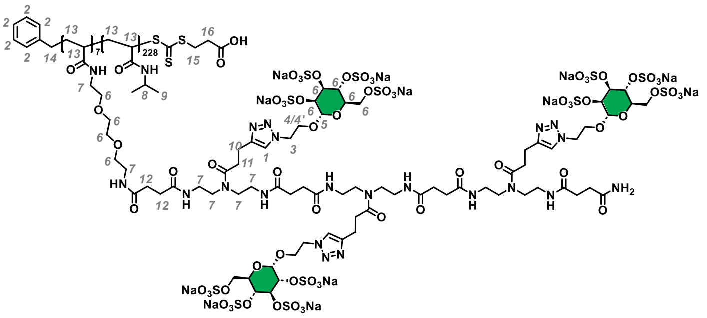

The synthesized polymer ***P22*** were obtained by the application of *standard protocol for global sulfation*, described in "1.3 General Methods". 13.08 mg (0.34 µmol) of reference structure ***P21*** were sulfated and received after dialysis as a white powder (12.16 mg, 0.27 µmol assuming complete sulfation, 79% yield, degree of sulfation: ^{1}H-NMR - complete low field shift of e.g. the triazole peak by 0.05 ppm.

^{1}H-NMR (300 MHz, D_2O) δ (ppm) 7.92 (s, *H1*), 7.43 – 7.16 (m, *H2*), 5.18 – 5.09 (m, *H5*), 4.72 – 3.76 (m, *H3, H4, H4', H6)*, 4.03 – 3.76 (m, *H8*), 3.76 – 3.57 (m, *H6*), 3.57 – 3.20 (m, *H7*), 3.07 – 2.94 (m, *H10*), 2.95 – 2.72 (m, *H11)*, 2.56 – 2.38 (m, *H12*), 1.26 – 0.83 (m, *H9*), 3.12– 0.00 (m, *H13- H16*).

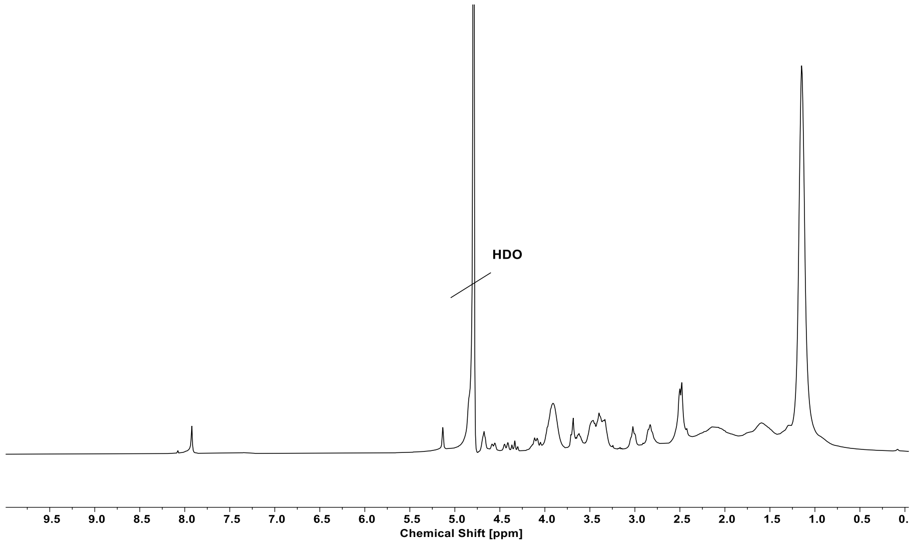

*Figure S103: ^{1}H-NMR spectra of sulfated $_{33}^{*}I_{235}$ (**P22**) (300 MHz, D_2O).*

P23 – ($_{0}\boldsymbol{I}_{235}$***)***

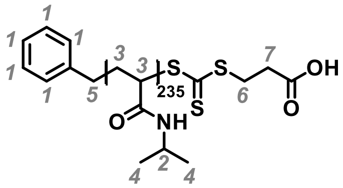

The synthesized polymer ***P23*** were obtained by the application of *synthesis of brush-like glycopolymers (arm-structures) and linear reference structures*, described in "1.3 General Methods". 33 mg (138.65 µmol, 0.59 µmol related to the repetition unit) of p(PFPA) ***P3*** were non functionalized with carbohydrates, only quenched with isopropylamine and were received after dialysis as a white powder (15.37 mg, 0.57 µmol, 97% yield).

^{1}H-NMR (300 MHz, D_2O) δ (ppm) 7.10 – 6.82 (m, *H1*), 3.74 – 3.44 (m, *H2*), 2.07 – 0.36 (m, *H3 – H*).

^{19}F-NMR (282 MHz, D_2O) δ (ppm) No signals of PFP-side chain determined.

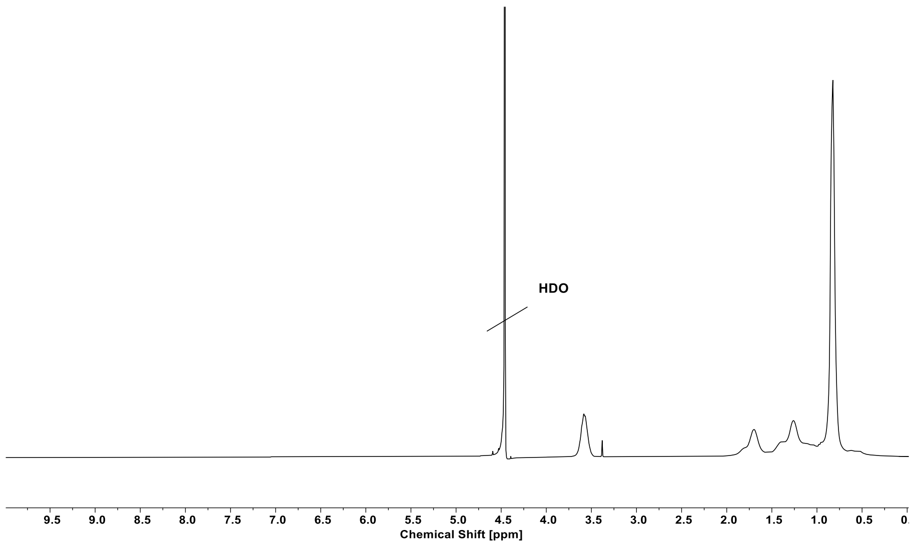

Figure S104: ^{1}H-NMR spectra of $\boldsymbol{I}_{235}$ ***(P23)*** *(300 MHz, D_2O).*

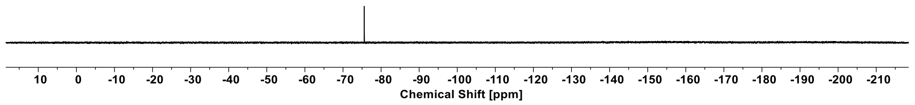

Figure S105: ^{19}F-NMR spectra of $\boldsymbol{I}_{235}$ (**P23**) (282 MHz, D_2O).

***P24* – ($_{0}E_{235}$)**

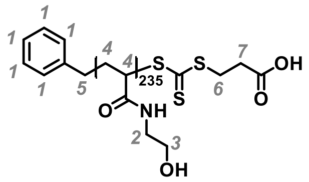

The synthesized polymer ***P24*** were obtained by the application of *synthesis of brush-like glycopolymers (arm-structures) and linear reference structures*, described in "1.3 General Methods". 38 mg (0.68 µmol, 159.66 µmol related to the repetition unit) of p(PFPA) ***P3*** were non functionalized with carbohydrates, only quenched with ethanolamine and were received after dialysis as a white powder (16.36 mg, 0.6 µmol, 88% yield).

^{1}H-NMR (300 MHz, D_2O) δ (ppm) 7.41 – 7.15 (m, *H1*), 4.00 – 2.82 (m, *H3*), 2.66 – 0.71 (m, *H7*), 2.26 – 1.16 (m, *H4 – H7*).

^{19}F-NMR (282 MHz, D_2O) δ (ppm) No signals determined.

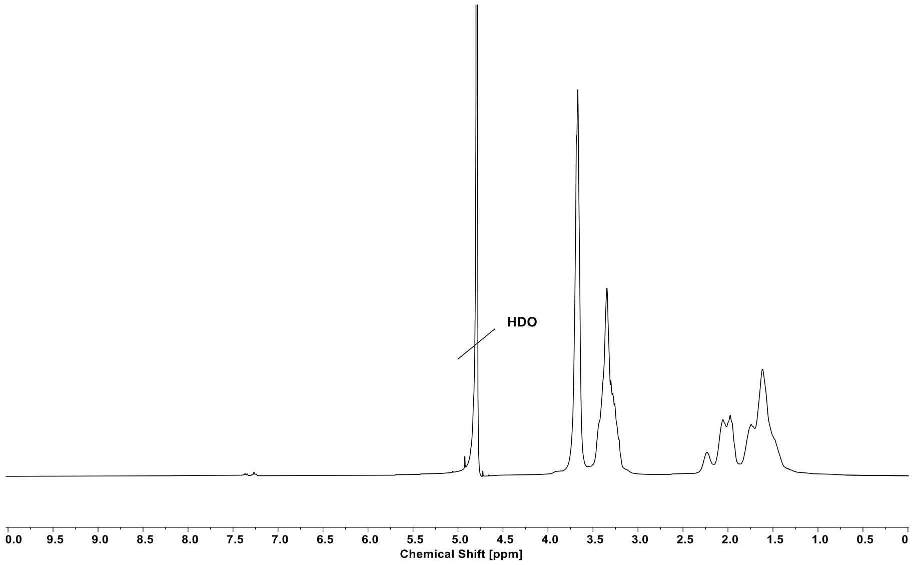

Figure S106: ^{1}H-NMR spectra of E_{235} ***(P24)*** *(300 MHz, D_2O).*

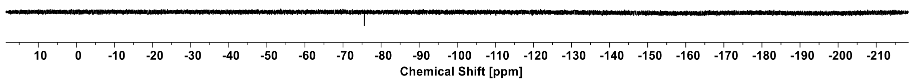

Figure S107: ^{19}F-NMR spectra of E_{235} ***(P24)*** *(282 MHz, D_2O).*

***B17* – ($_{0}E^{2}_{57}$)**

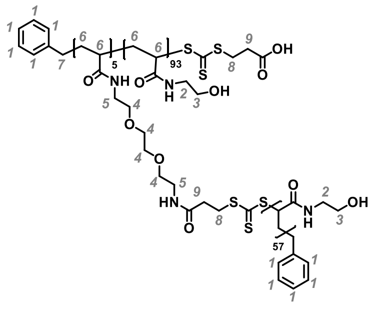

The synthesized brush² glycopolymer ***B17*** were obtained by the application of two steps: (1) *Attachment of the bridge molecule to arm polymers,* followed by *Cleavage of the protecting group of the conjugated bridge molecule* (2) *Attachment of the amine-activated arm polymers to the long polymer to gain brush² structures* described in "1.3 General Methods". 10 mg (0.18 µmol, 42.02 µmol related to the repetition unit) of p(PFPA) Pn ~ 98 were functionalized with amine-preactivated arm polymer with comes from **P5** (5%, 14.83 mg, 2.10 µmol) quenched with ethanolamine and were received after dialysis as a white powder (12.06 mg, only theoretically 5% incorporation, 0.11 µmol, 61% yield).

^{1}H-NMR (600 MHz, D_2O) δ (ppm) 7.45 – 7.10 (m, *H1*), 3.99 – 3.05 (m, *H2*, *H3*), 2.98 – 2.85 (m, *H4*), 2.84 – 0.76 (m, *H5 – H9*).

^{19}F-NMR (564 MHz, D_2O) δ (ppm) No signals determined.

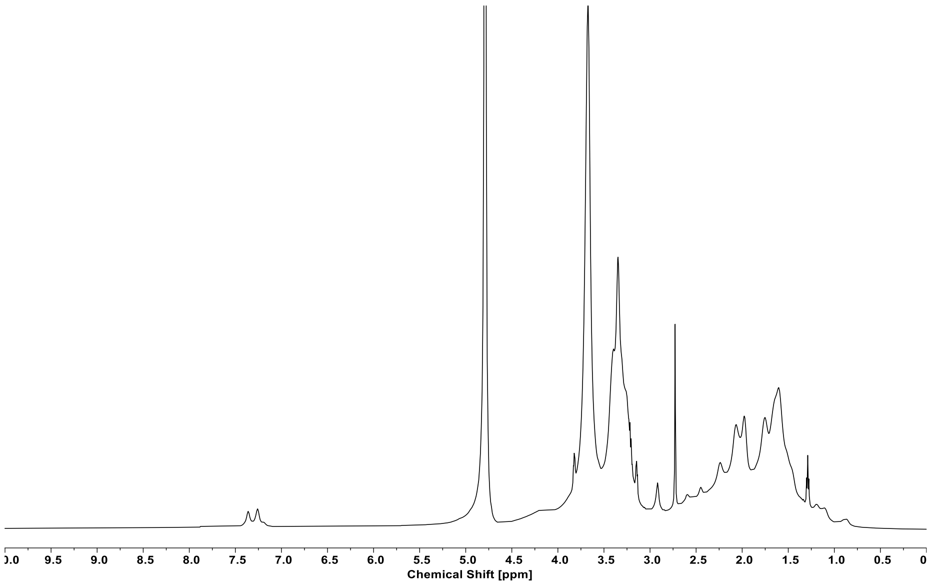

Figure S108: ^{1}H-NMR spectra of **($_{0}E^{2}_{57}$)** ***(B17)*** *(600 MHz, D_2O).*

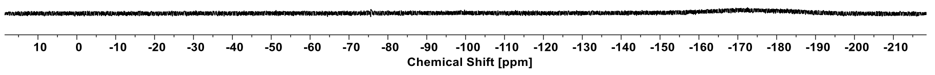

Figure S109: ^{19}F-NMR spectra of **($_{0}E^{2}_{57}$)** ***(B17)*** *(564 MHz, D_2O).*

2.7 DLS measurement

Table 2: Summary of the DLS results with a respective concentration of 0.5 mg/mL water. The limited reliability of the particle sizer in complex distributions (bi- or multimodal) - as partly observed in our AFM measurements - probably explains the deviations of individual DLS measurements from the trend.

structue type	label		temp. [°C]#	d_h [nm]#
**brush^2** 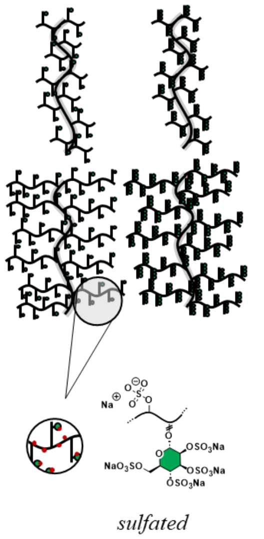	$_{22}E^{2}_{29}$	**B1**	20	80.0
	$_{66}E^{2}_{29}$	**B2**	20	65.6
	$_{22}I^{2}_{29}$	**B3**	20	92.0
	$_{66}I^{2}_{29}$	**B4**	20/40	102.6/99.8
	$_{66}E^{2}_{57}$	**B5**	20	113.0
	$_{198}E^{2}_{57}$	**B6**	20	73.0
	$_{66}I^{2}_{57}$	**B7**	20/40	184.2/320.4
	$_{198}I^{2}_{57}$	**B8**	20/40	123.0/445.3
	$_{22}^{*}E^{2}_{29}$	**B9**	20	149.6
	$_{66}^{*}E^{2}_{29}$	**B10**	20	77.1
	$_{66}^{*}E^{2}_{57}$	**B11**	20	67.4
	$_{198}^{*}E^{2}_{57}$	**B12**	20	67.1
	$_{22}^{*}I^{2}_{29}$	**B13**	20/40	179.8/118.5
	$_{66}^{*}I^{2}_{29}$	**B14**	20/40	73.6/61.5
	$_{66}^{*}I^{2}_{57}$	**B15**	20/40	108.7/498.7
	$_{198}^{*}I^{2}_{57}$	**B16**	20/40	118.7/96.7
**control structures** 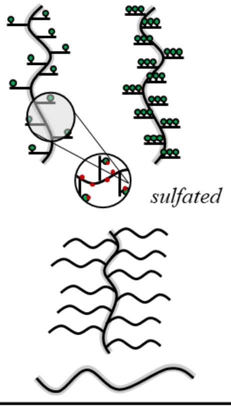	$_{11}E_{235}$	**P15**	20	65.6
	$_{33}E_{235}$	**P16**	20	118.3
	$_{11}I_{235}$	**P17**	20/40	50.8/69.9
	$_{33}I_{235}$	**P18**	20/40	71.4
	$_{11}^{*}E_{235}$	**P19**	20	98.4
	$_{33}^{*}E_{235}$	**P20**	20	107.1/16.4
	$_{11}^{*}I_{235}$	**P21**	20/40	87.5/61.5
	$_{33}^{*}I_{235}$	**P22**	20/40	97.2/83.0
	$_{0}E^{2}_{57}$	**B17**	20	87.9
	$_{0}E_{235}$	**P23**	20	107.8
	$_{0}I_{235}$	**P24**	20/40	137.2/208.5

determined via DLS

2.8 SANS measurement

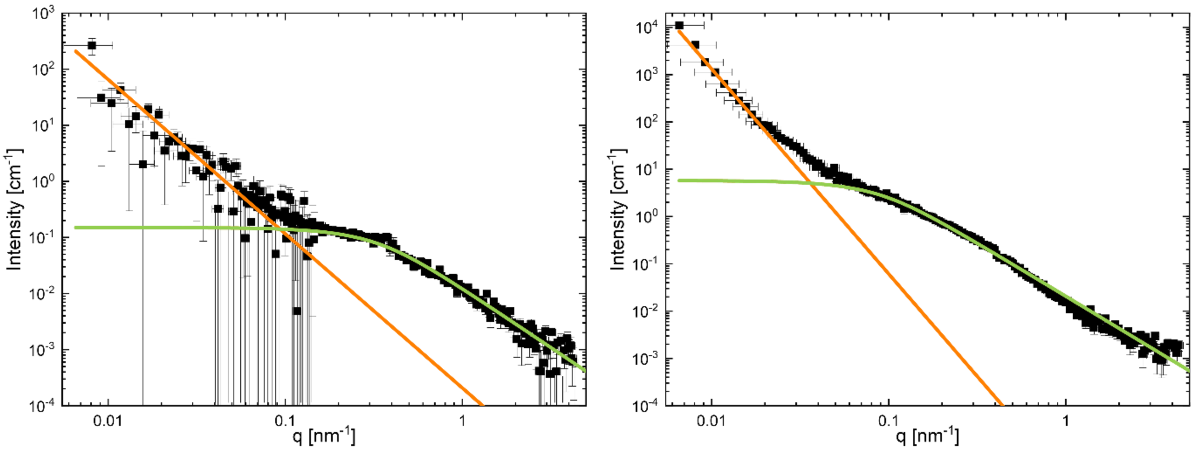

*Figure S110: Results from SANS measurements. The measurements were performed at 25 °C. Both samples were measured in D_2O with concentrations of 5 mg/ml. The Data of sample **B6** is shown on the left and of sample **P15** on the right. The measured data is shown as dots and the contributions to the fits are shown as a solid line. The power law at low q is shown in orange and the Gaussian coil fit is shown in green. The incoherent background scattering was subtracted.*

2.9 AFM measurement

Desorption by AFM-Based Single Molecule Force Spectroscopy

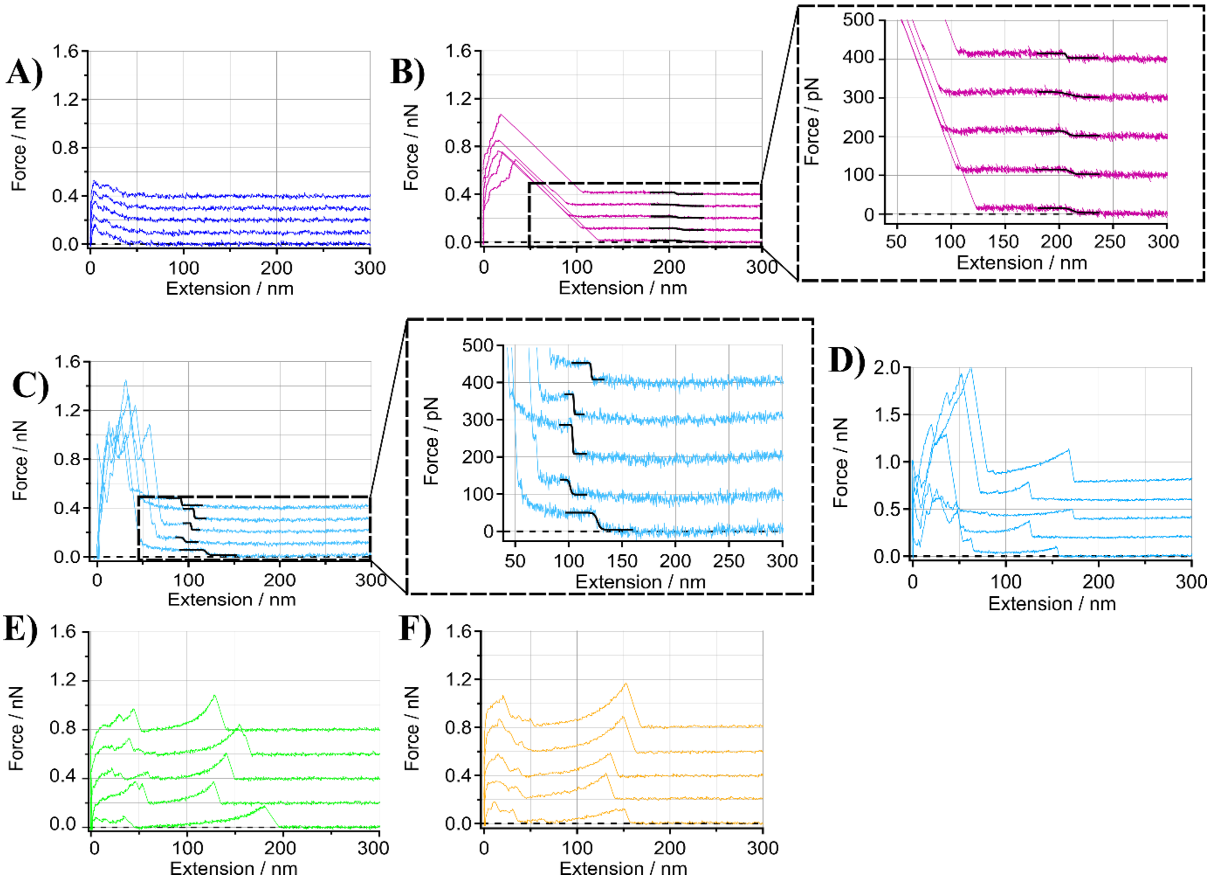

*Figure S111. Exemplary single molecule desorption force-extension curves for A) triethoxysilane-PEG-methoxy (5 kDa) and triethoxysilane-PEG-amine (5 kDa) in a 50:1 ratio on mica in water (control measurements) and B-F) polymers **B17**, **P15**, **P19** and **B11** on mica in water. The extensions range between 100-200 nm, which are consistent with the lengths of the synthesized polymers, which are expected to have a backbone of around 72 nm (based on the C-C length of 1.53 Å[12] for the repeating unit of 235 monomers), plus the PEG linker (ca. 41 nm).[13] The polydispersity index (PDI) of the polymer (1.37) leads to variability in the observed extensions, allowing for some molecules to extend beyond the sum of the backbone and linker lengths, reaching up to 200 nm. The curves either exhibit different desorption motifs, such as plateaus of constant force, as observed for B, C) polymers **B17** and **P15**, or a single desorption peak, as for D-F) polymers **P15**, **P19** and **B11**. We show the whole force-extension curve with a zoom into the relevant plateau portions in (B, C). Plateaus of constant force are indicated with a sigmoidal fit, used for the plateau force and extension evaluation. To improve the presentation, we have vertically offset the curves for (A-C) 100 or (D-F) 200 pN.*

We performed our AFM force spectroscopy experiments in pure water to specifically investigate hydrogen-bond-driven interactions between the glycopolymers and the mica substrate. While buffer conditions would better mimic physiological environments, water was chosen as a clean and suitable medium to investigate the desorption behavior. To further assign the interactions to hydrogen bonds, we performed experiments with **P15** and **B11** on mica using HEPES (10 mM, 50 mM NaCl, pH 7.0 adjusted with NaOH), which can align with the hexagonal lattice of mica and effectively block hydrogen bonding sites.[14] Furthermore, this formation of the hydrogen bonds might be reduced by water molecules aligning to the OH groups of the HEPES molecules. Altogether, this resulted in a loss of detectable interaction events, supporting our claim, that the interactions observed in water are primarily due to hydrogen bonds.

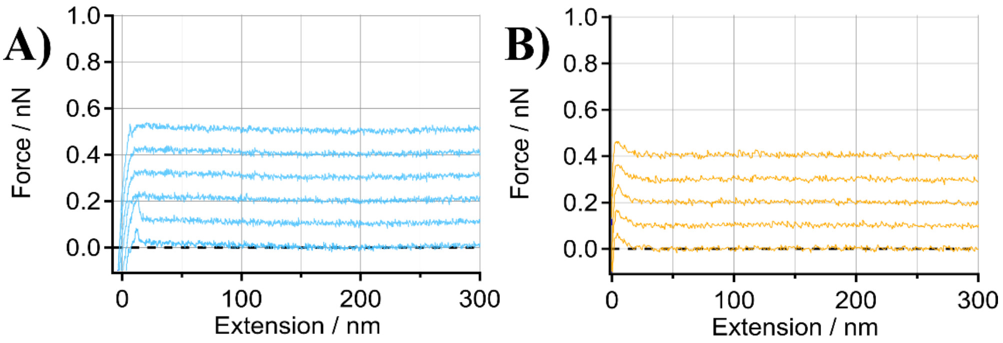

*Figure S112. Exemplary single molecule desorption force-extension curves for A) polymer **P15** and B) polymer **B11** on mica in HEPES (10 mM, 50 mM NaCl, pH 7.0 adjusted with NaOH). In contrast to measurements in pure water (S111) no single molecule desorption motifs are visible, most likely due to HEPES blocking hydrogen bonding sites by aligning with the hexagonal lattice of mica.*

Calculation hydrogen bond density (normalized for based on the backbone length)

One monomer unit contributes two C-C bonds to the backbone length. With 1.53 Å per C-C bond[12] and $N = 235$, this leads to a backbone length of $L_b = 2 \cdot 0.153\ nm \cdot 235 = 72\ nm$. The structure **B17** has no mannose. **P15** and **P19** both have 11 side arms, each containing one mannose group, with each mannose contributing four free groups (hydroxyl for **P15,** hydroxyl and sulfate for **P19**) for hydrogen bonds. Compared to **B17**, this leads to an increase of the potential density of hydrogen bonds per nm of $\rho_{P15,\,P19} = \frac{11 \cdot 4}{72\ nm} = 0.6\ \frac{H\text{-}bonds}{nm}$. **B11** has 11 side arms, each containing six mannose group, with each mannose contributing four free groups (hydroxyl and sulfate) for hydrogen bonds. Compared to **B17**, this leads to an increase of the potential density of hydrogen bonds per nm of $\rho_{B11} = \frac{66 \cdot 4}{72\ nm} = 3.6\ \frac{H\text{-}bonds}{nm}$.

Conformations by AFM-Based Imaging

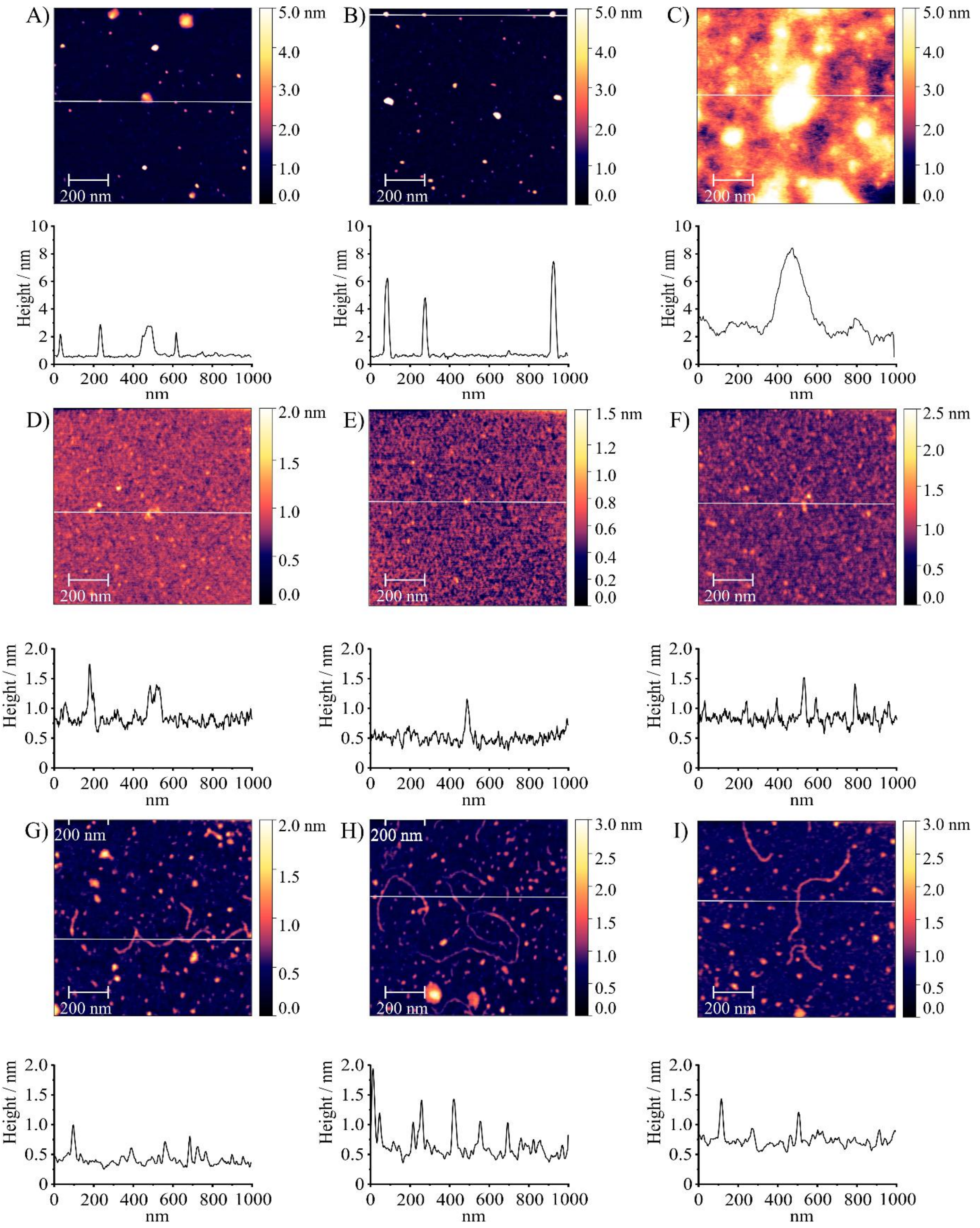

*Figure S113: AFM topography images (size: 1 µm^2) revealing different conformations of A-C) polymer **B17** (brush²), D-F) polymer **P19** (linear) and G-I) polymer **B11** (brush²), in analogy to the AFM topography images in Figure 6 (main text), all taken on different mica surface positions. All polymers were deposited from a 0.1 µg mL^{-1} polymer solution in water and then dried under vacuum. Images were obtained in air. Line scans are given below the topography images, their positions are indicated by white lines in the topography images.*

B17 (Figure S113 A-C), without any mannose, shows a high degree of aggregation, whereas the glycopolymers **P19** and **B11** (Figure S113 D-I), containing mannose, form smaller aggregates, or in the case of **B11** even linear aggregates. The compact aggregates for brush glycopolymer **B17** (Figure S113 A-C) are clearly observed in the line profiles, where the surface itself is rather clean and flat, interrupted by features reaching heights of up to 9 nm. They might represent a larger aggregate or collapsed bundle. This is different for both, **P19** and **B11** (Figure S113 D-I), where the surface is uneven, showing small aggregates with collapsed conformations all over the line profile, reaching heights of up to 2 nm. This might be a sign of the polymers interacting with the substrate surface, likely mediated by multivalent interactions involving the mannose moieties. For **B11** (Figure S113 G-I), extended, linear conformations are visible with heights up to 1 nm. The width of all the structures observed in the AFM images ranges from 20 nm to 50 nm, except for the large aggregates in the case of **B17** (Figure S113 A-C). Please note that these width values are prone to tip convolution as the cantilever has a tip radius of 5 nm.

3. Literature

(1) Dewhurst, C. Graphical reduction and analysis small-angle neutron scattering program: GRASP. *J. App. Crystallogr.* **2023**, *56* (5), 1595.
(2) Zemplén, G.; Pacsu, E. Über die Verseifung acetylierter Zucker und verwandter Substanzen. *Berichte der deutschen chemischen Gesellschaft (A and B Series)* **1929**, *62* (6), 1613-1614.
(3) Hutter, J. L.; Bechhoefer, J. Calibration of atomic-force microscope tips. *Rev.Sci. Instrum.* **1993**, *64* (7), 1868-1873.
(4) Pirzer, T.; Geisler, M.; Scheibel, T.; Hugel, T. Single molecule force measurements delineate salt, pH and surface effects on biopolymer adhesion. *Phys. Biol.* **2009**, *6* (2), 025004.
(5) Nečas, D.; Klapetek, P. Gwyddion: an open-source software for SPM data analysis. *Open Phys.* **2012**, *10* (1), 181-188.
(6) Ponader, D.; Wojcik, F.; Beceren-Braun, F.; Dernedde, J.; Hartmann, L. Sequence-defined glycopolymer segments presenting mannose: synthesis and lectin binding affinity. *Biomacromolecules* **2012**, *13* (6), 1845-1852.
(7) Ebbesen, M.; Gerke, C.; Hartwig, P.; Hartmann, L. Biodegradable poly (amidoamine) s with uniform degradation fragments via sequence-controlled macromonomers. *Polym. Chem.* **2016**, *7* (46), 7086-7093.
(8) Hill, S. A.; Gerke, C.; Hartmann, L. Recent Developments in Solid-Phase Strategies towards Synthetic, Sequence-Defined Macromolecules. *Chem. Asian J.* **2018**, *13* (23), 3611-3622.
(9) Illmann, M. D.; Schäfl, L.; Drees, F.; Hartmann, L.; Schmidt, S. Glycan-Presenting Coacervates Derived from Charged Poly (active esters): Preparation, Phase Behavior, and Lectin Capture. *Biomacromolecules* **2023**, *24* (6), 2532-2540.
(10) Graisuwan, W.; Zhao, H.; Kiatkamjornwong, S.; Theato, P.; Hoven, V. P. Formation of thermo-sensitive and cross-linkable micelles by self-assembly of poly (pentafluorophenyl acrylate)-containing block copolymer. *J. Polym. Sci. A Polym. Chem.* **2015**, *53* (9), 1103-1113.
(11) Shamout, F.; Monaco, A.; Yilmaz, G.; Becer, C. R.; Hartmann, L. Synthesis of Brush-Like Glycopolymers with Monodisperse, Sequence-Defined Side Chains and Their Interactions with Plant and Animal Lectins. *Macromol. Rapid. Commun.* **2020**, *41* (1), 1900459.
(12) Haynes, W. M. *CRC Handbook of Chemistry and Physics*; CRC press, 2016.
(13) Kolberg, A.; Wenzel, C.; Hackenstrass, K.; Schwarzl, R.; Rüttiger, C.; Hugel, T.; Gallei, M.; Netz, R. R.; Balzer, B. N. Opposing temperature dependence of the stretching response of single PEG and PNiPAM polymers. *J. Am. Chem. Soc.* **2019**, *141* (29), 11603-11613.
(14) Trewby, W.; Livesey, D.; Voïtchovsky, K. Buffering agents modify the hydration landscape at charged interfaces. *Soft Matter* **2016**, *12* (9), 2642-2651.
